# Autologous skin-derived neural precursor cell therapy reverses canine Alzheimer dementia-like syndrome in a proof of concept veterinary trial

**DOI:** 10.1186/s13287-022-02933-w

**Published:** 2022-06-17

**Authors:** Michael Valenzuela, T. Duncan, A. Abey, A. Johnson, C. Boulamatsis, M. A. Dalton, E. Jacobson, L. Brunel, G. Child, D. Simpson, M. Buckland, A. Lowe, J. Siette, F. Westbrook, P. McGreevy

**Affiliations:** 1 Skin2Neuron Pty Ltd, Sydney, Australia; 2 University of New South Wales, Sydney, Australia; 3 University of Sydney, Sydney, Australia; 4 Sydney Children’s Hospital, Sydney, Australia; 5 Western Sydney University, Sydney, Australia; 6 Royal Prince Alfred Hospital, Sydney, Australia; 7 Animal Referral Hospital Homebush, Sydney, Australia; 8 University of New England, Armidale, Australia

**Keywords:** Alzheimer’s disease, Dementia, Hippocampus, Cell therapy, Stem cells, Neural precursors, Canine, Rodent

## Abstract

**Background:**

Older companion dogs naturally develop a dementia-like syndrome with biological, clinical and therapeutic similarities to Alzheimer disease (AD). Given there has been no new safe, clinically effective and widely accessible treatment for AD for almost 20 years, an all-new cell therapeutic approach was trialled in canine veterinary patients, and further modelled in aged rats for more detailed neurobiological analysis.

**Methods:**

A Phase 1/2A veterinary trial was conducted in *N* = 6 older companion dogs with definitive diagnosis of Canine Cognitive Dysfunction (CCD). Treatment comprised direct microinjection of 250,000 autologous skin-derived neuroprecursors (SKNs) into the bilateral hippocampus using MRI-guided stereotaxis. Safety was assessed clinically and efficacy using the validated Canine Cognitive Dysfunction Rating Scale (CCDR) at baseline and 3-month post treatment. Intention to treat analysis imputed a single patient that had a surgical adverse event requiring euthanasia. Three dog brains were donated following natural death and histology carried out to quantify Alzheimer pathology as well as immature neurons and synapses; these were compared to a brain bank (*N* = 12) of untreated aged dogs with and without CCD. Further, an age-related memory dysfunction rat model (*N* = 16) was used to more closely evaluate intrahippocampal engraftment of canine SKN cells, focusing on mnemonic and synaptic effects as well as donor cell survival, neurodifferentation and electrophysiologic circuit integration in a live hippocampal slice preparation.

**Results:**

Four out-of-five dogs improved on the primary clinical CCDR endpoint, three fell below diagnostic threshold, and remarkably, two underwent full syndromal reversal lasting up to 2 years. At post mortem, synaptic density in the hippocampus specifically was nine standard deviations above non-treated dogs, and intensity of new neurons also several fold higher. There was no impact on AD pathology or long-term safety signals. Modelling in aged rats replicated the main canine trial findings: hippocampally-dependent place memory deficits were reversed and synaptic depletion rescued. In addition, this model confirmed donor cell survival and migration throughout the hippocampus, neuronal differentiation in situ*,* and physiologically-correct integration into pyramidal layer circuits.

**Conclusions:**

With further development, SKN cell therapy may have potential for treating carefully chosen AD patients based on neurosynaptic restoration in the hippocampus.

**Supplementary Information:**

The online version contains supplementary material available at 10.1186/s13287-022-02933-w.

## Background

Dementia is a devastating clinical syndrome that typically begins with amnesia and progresses to cognitive, behavioural and neuropsychiatric impairment, and in final stages, dysphagia and incontinence. Worldwide, 48 million people are affected, with advanced age the strongest risk factor followed by APOE4 genotype [1]. The proximate biological cause of Alzheimer’s disease (AD)-related dementia is a mass loss of neurons and synapses [2, 3], initially in the medial temporal lobe and ultimately severe enough to destroy network function [4]. Multiple upstream pathologies are implicated beyond the classic AD hallmarks of extracellular amyloid plaques and intra-neuronal tauopathic inclusions, these commonly co-occurring alongside other neurodegenerative diseases [5, 6].

Despite recent FDA approval of the first disease-modifying drug for AD (aducanumab) there has been no new clinically effective and widely accessible dementia treatment since 2004 [7], and so innovative therapeutic approaches are urgently needed. Cell therapy aims to restore and regenerate neurons and synapses by donor cellular integration into functional circuits and paracrine mechanisms [8], and more recently by application of extracellular vesicles such as cell-derived exosomes [12]. Rodent studies have provided proof-of-concept using different cell types and models (reviewed by Duncan & Valenzuela [8]), and a single Phase I clinical trial of direct brain injection of mesenchymal stromal cells (MSCs) found the procedure was safe but of no therapeutic consequence [9].

Several issues have limited clinical development. Induced pluripotential stem (IPS) cell technologies lack manufacture control in terms of genomic instability and variability between clones [10]_,_ donor-related lineage bias [11], and low donor success rates. MSCs do not survive in the mammalian brain for more than a few weeks [13], and glial differentiation is common in vivo [14]. More generally, progress has been impeded by lack of a clinically predictive and biologically relevant large-animal model [15].

In response, we developed a suite of technologies for diagnosing and treating Canine Cognitive Dysfunction (CCD) that naturally displays many of the same clinical signs as human AD dementia [16, 17]. Prevalence of CCD rises exponentially with age [18] and CCD is associated with brain atrophy [19, 20], beta-amyloid pathology [21–23], hyperphosphorylated tau pathology [24] distributed in the human Braak-like pattern [25], and loss of midbrain neurons that innervate the hippocampus [26]. Notably, CCD occurs spontaneously on a diverse genetic background and dogs are universally homozygous for AD risk gene APOE4 [27]. For these reasons, there is increasing interest in CCD as a large-animal translational model of AD that exhibits a dementia-like syndrome [28–31].

At the same time, we developed a new process for the culture of unipotent canine *skin-derived neural precursors* (SKNs) [32]. SKNs are defined by a CD271^+^Nestin^+^CD133^+^ phenotype and for a number of reasons are attractive for cell therapy. These cells are free of genetic modification and in a detailed characterization study [33] we have shown: SKNs do not express pluripotential markers; can undergo reliable and uniform manufacture from a small sample of donor skin; are capable of in vitro differentiation into functional neurons; and do not express glial protein or genes.

Here, we therefore report on the first veterinary trial of SKN cell therapy for the treatment of CCD. Treatment comprised a dose of 250,000 autologous SKN cells microinjected stereotaxically into both hippocampi using patient-specific MRI-based co-ordinates. In order to address some open questions from the trial, we further modelled the therapeutic process in aged rodents with hippocampally selective memory and synaptic dyfunction.

## Methods

### Animal models

#### Canine trial

This trial was approved by the University of Sydney Animal Ethics Committee, and because it spanned 8 years (2012–2020) the trial encompassed several protocol updates. These protocol changes are summarised in Additional file 1: Supplementary Materials and full versions of the therapeutic protocols are also included to aid in replication.

The experimental model was Canine Cognitive Dysfunction, diagnosed in aged companion dogs (*N* = 6, 2 female, 4 male, aged 10–16 years see Additional file 1: Table ST1) in the following manner: First, a provisional CCD diagnosis was provided by an initial CCDR score >  = 50 that requires evidence of both neurobehavioural dysfunction and a history of decline over the last 6-months. CCDR-based diagnoses has been validated against clinical diagnosis of canine dementia with an accuracy of > 98% [16, 18], overall item reliability was high (Chronbach’s alpha = 0.86), and test–retest reliability established with an intraclass coefficient of *r* = 0.73 and absolute difference between iterations of 0.2 points (SD 2.3), translating to a Reliable Change Index of ± 5-points. Improvement on the CCDR by more than 5 points was therefore used to classify therapeutic responders.

Next, a definitive diagnosis of CCD was made after exclusion of potential biological causes of abnormal canine behaviour apart from AD, such as concurrent illness, systemic infection, pain, intracranial mass, renal failure, polypharmacy (and some specific medications), diabetes or endocrine disorder, and confirmed by a consultant veterinary neurologist.

The primary endpoint of the trial was the standard published CCDR at baseline and a modified CCDR at follow-ups, where change-over-time items were compared to “before treatment” as opposed to “6-months ago” as in the standard CCDR.

The trial was designed to PREPARE guidelines, including protocol registration PCTE0000169 on www.preclinicaltrial.eu (see Additional file 1: Table ST1 for individual patient details and Additional file 2: Supplementary Materials for protocol) and reported here to ARRIVE guidelines.

## Rodent study

### Main study

Aged animals were 16 experimentally naïve 18–19 month old female ex-breeder Fischer 344 rats. Rats were randomly assigned to a cell transplant or sham media surgery condition and were tested before and after treatment [i.e. 2 (× 2) repeated measures design] (see main manuscript Fig. 4A).

All procedures were approved by the Animal Care and Ethics Committee at the University of New South Wales and conducted in accordance with the National Institutes of Health (NIH) *Guide for the Care and Use of Laboratory Animals* (NIH Publications No. 80-23) and the National Health and Medical Research Council Guidelines. None of the aged animals developed brain tumours.

### Extension study

We transplanted a further *N* = 2 aged (21-month old) female Fischer rats with GFP-labelled SKN cells (lentivirus [34] gift of H. Praag) for the purpose of confirming hippocampal engraftment and investigating co-expression with mature neuronal markers, neurofilament (NF) and NeuN.

## Experimental procedures

### Canine trial

#### General overview

Clinical trial patients were sourced either through referral from veterinary clinics in the Greater Sydney region, through an online CCDR Register or through direct online owner enquiry to the Regenerative Neuroscience Group, the University of Sydney. All participants in the clinical trial were privately owned pets, and all owners/carers provided informed consent.

Dogs were required to satisfy selection criteria to be eligible for trial enrolment: aged 8 years or older and score ≥ 50 on the CCDR scale; must not suffer from severe separation anxiety; must have a normal blood panel (including T4) and a normal urine panel; must have a reasonable level of mobility, a reasonable level of vision and a body condition score ≤ 4; must not be on high dose oral corticosteroids and must pass a veterinary ‘fitness for trial’ checklist. Dogs must not suffer from any behavioural, medical or neurological condition which may present with similar clinical signs to CCD.

Over the course of weeks 0–2, baseline physical activity was recorded using a wearable device, attached to the dog’s collar. Baseline activity was recorded for one week. The dog continued to wear the device throughout its involvement in the trial. During this period, patients underwent neurological assessment by a veterinary neurologist to provide confirmation of the provisional CCD diagnosis.

During weeks 2–4, the patient underwent three procedures under a single general anaesthetic, referred to as the “first procedure”: skin biopsy, MR imaging of the brain and the implantation of fixed fiduciary screws into the skull. The skin sample was transferred to the RNG lab and processed for the production of autologous SKNs (see Methods below). MRI was acquired to produce patient-specific stereotaxic targets for cell delivery into the hippocampus (see Methods below) as well as exclude structural brain abnormalities that may contribute to behavioural change unrelated to Alzheimer’s (e.g. cranial neoplasm, adenoma). Fixed screws were implanted to aid in skull/brain co-registration between MRI images and head position on the stereotaxic frame during surgery (see Methods below).

Two weeks prior to cell transplant, and for 4 weeks after transplant, the owner was instructed to double their dog’s level of exercise. The dog’s activity level was monitored during this time using a wearable device.

Skin sutures were removed 10–14 days following skin biopsy and screw placement. At this time, the dog’s primary carer repeated the CCDR. This score was considered to be the pre-therapeutic reference baseline score. At this time point, the dogs’ spatial memory was assessed in the Canine Sand Maze.

Approximately 4–5 weeks post the first procedure, during which the SKN cells were manufactured, each patient underwent hippocampal microinjection of their autologous skin-derived neural precursor cells (SKNs) under general anaesthesia (“second procedure”). In preparation for cell transplant, the patient was administered oral levetiracetam for 48 h pre-operatively to reduce the risk of post-operative seizure activity. Post operatively, patients remained under the care of the veterinary specialist team until suitable for discharge and levetiracetam was continued for one week.

Follow-up veterinary assessments were carried out at 1-month, 3-months (CCDR change primary endpoint) and 6-month post-operative time points, and then annually for life. Repeat spatial memory assessment was repeated in the Canine Sand Maze at 3-month and 6-month follow-ups.

With the owner’s permission, the participant’s brain was donated at the time of elective euthanasia to the research group’s Canine Brain Bank for histological analysis of Alzheimer’s disease pathology, synaptic markers and assessment of anomalous cell growth.

### Skin-derived neuroprecursor (SKN) culture and cell preparation

Subject’s abdominal skin tissue (≈12 cm^2^) was taken under general anaesthetic following owner consent. Neural precursor cell lines were established using our published protocol. [33]

Briefly, skin biopsies were cut into 1–2 mm pieces and enzymatically digested in 0.1% Trypsin (Thermo Fisher Scientific Australia Pty Ltd, Scorseby, VIC, Australia) at 37 °C for 40 min, followed by 0.1% DNAse (Roche Applied Science, Castle Hill, NSW, Australia) at room temperature for 1 min. The was then dissociated by mechanical maceration and passed through a 40 µm cell strainer (BD Bioscience, Sydney, NSW, Australia). The resultant cell suspension was centrifuged at 180G for 5 min. The cell pellet was re-suspended in serum free growth medium consisting of 3:1 DMEM/F12 medium (Thermo Fisher Scientific Australia Pty Ltd, Scorseby, VIC, Australia), 1% Penicillin Streptomycin (Thermo Fisher Scientific Australia Pty Ltd, Scorseby, VIC, Australia), 20 ng/mL EGF (BD Bioscience, Sydney, NSW, Australia), 40 ng/mL bFGF (Thermo Fisher Scientific Australia Pty Ltd, Scorseby, VIC, Australia), and 2% B-27 (Thermo Fisher Scientific Australia Pty Ltd, Scorseby, VIC, Australia). The cells were then seeded into non-tissue culture treated dishes at a density of 100,000 cells/cm and incubated at 37 °C. After 5 days, when the resulting neurospheres have reached ~ 50 µm in diameter, they were removed from the media by centrifugation and dissociated in TrypLE Select (Thermo Fisher Scientific Australia Pty Ltd, Scorseby, VIC, Australia) at 37 °C for 5 min followed by agitation of the suspension by manual pipetting. The resulting single cells were re-suspended in serum free growth medium, seeded at a density of 10,000 cells/cm on 0.1% gelatin coated flasks, and passaged weekly or when at 80% confluency, for 3 weeks.

In preparation for transplantation, cells were enzymatically released from the culture surface using TrypLE Select at 37 °C for 5 min. The cells were then washed in three changes of PBS (Thermo Fisher Scientific Australia Pty Ltd, Scorseby, VIC, Australia), centrifuged at 180G for 10 min, and transferred into a microvial in PBS.

### Brain imaging

All MRI brain imaging (except Sasha) was conducted on the Animal Referral Hospital’s Siemens Magneton Avanto 1.5 Tesla system. Sasha’s MRI was acquired at University Veterinary Teaching Hospital, Sydney using a Esaote Vet Grande 0.25 Tesla System. As both systems are clinical scanners, image acquisition protocols varied over time and image quality was impacted by hardware and software changes. However, for the purpose of stereotaxic hippocampal targeting, these changes were not relevant.

Before acquisition of the 3D T1 weighted MR image, a series of scout scans are performed to ensure the alignment of the head within the scanner. Dogs are scanned in sternal recumbency with the head extended. The coronal, sagittal and transverse planes are set in the MRI System software, but several scout scans are performed to ensure the head is aligned in the following way: that the ventral aspect of the tympanic bullae are horizontal (X-axis); that the line transecting the external sagittal crest and the midline of the brain between the two cerebral hemispheres is vertical (Z-axis), and that the line transects the insertion site of the two titanium screws at the level of the skull surface is horizontal (Y-axis).

### Image parameters

Sasha: 3-dimensional gradient-echo T1-weighted sequence (512 × 512 matrix; slice thickness = 1.2 mm; voxel size = 0.37 × 0.37 × 1.2; TR = 38 ms; TE = 16 ms; flip angle = 75°).

Timmy and Leo: 3-dimensional gradient-echo T1-weighted sequence (256 × 256 matrix; slice thickness = 1 mm; voxel size = 1.02 × 1.02 × 1; TR = 38 ms; TE = 16 ms; flip angle = 75°).

Gracie: 3-dimensional gradient-echo T1-weighted sequence (192 × 192 matrix; slice thickness = 0.9 mm; voxel size = 0.52 × 0.52x0.9; TR = 1900 ms; TE = 3.43 ms; flip angle = 15°).

Grover and Gus: 3-dimensional gradient-echo T1-weighted sequence (384 × 384 matrix; slice thickness = 0.8 mm; voxel size = 0.52 × 0.52x0.8; TR = 1690 ms; TE = 3.78 ms; flip angle = 9°).

### Neurosurgery and patient-specific stereotaxis

Hippocampal targeting was challenging in a veterinary hospital setting because of a tolerance of ± 2 mm in xyz directions, and the absence of a frameless stereotaxic system as often used in human neurosurgery.

We therefore first developed and validated a procedure on canine and sheep cadavers that achieved hippocampal targeting verified by injection of dye and immediate PM inspection of the hippocampus. This protocol involved implantation of two fixed fiduciary markers onto the canine skull in the form of small 2 mm titanium screws. Two screws were implanted into the skull longitudinally along the midline: one anteriorly at the symphysis of the temporal line and sagittal crest, and the second 20 mm posteriorly also along the sagittal crest.

The purpose of these fiduciary markers was to allow co-registration between the 3D brain images captured by MRI, and the skull and brain whilst within the stereotaxic apparatus (KOPF, Model 1530 frame using Model 1460 XYZ Electrode manipulator and Model 1772 Universal Holder) on the surgical table. MRI images were rotated by rigid-body transformation such that the two screws (visible as image artefacts) were in the horizontal plane to control pitch rotation, in-line antero-posteriorly when viewed axially (using human brain imaging conventions) to control yaw rotation, and the auditory canals in the horizontal plane on coronal slice to control roll rotation. Hippocampal targets were then calculated as XYZ displacements from the middle of the posterior screw.

These ideal target conditions were replicated on the surgical table by using the needle tip attached to the stereotaxic frame to ensure the two screws were positioned horizontal and longitudinally in alignment, and by the ear bars fitting into the same peri-auricular fossa (or submandibular fossa depending on size of skull). Hippocampal targets were subsequently achieved by reference to the 0,0,0 point being the middle of the posterior screwhead.

Sasha, Timmy, Leo and Gracie underwent this stereotaxic protocol (Additional File 3: neurosurgical procedures). Because of difficulties in achieving the desired skull position in Gracie (the largest dog physically), we improved the process by allowing for measurement of deviation from the idealized skull position, using the needle tip to measure xyz offsets. These offset measurements were imported into a calculator in real time such that revised hippocampal XYZ targets were used that accounted for the *achieved* dog head position on the surgical table. Grover was treated using this method.

Finally, a major refinement was carried out for Gus whereby surgery was moved to the Sydney University Hybrid Theatre, which allows for co-registration between the patient’s CT and MRI, and then real time interactive CT imaging on the surgical table, allowing the surgeon to visualise the needle in the brain and progressing the needle tip to the hippocampal target.

Neurosurgery proceeded by positioning the anaesthetised and intubated dog onto the stereotaxic frame, preparing the surgical field, and exposing the skull by longitudinal incision of the overlying soft tissues and retraction of masseter muscle fibres. Two burr holes (approximately 3 mm wide) were drilled bilaterally using a neurosurgical drill. A 25G needle (Hamilton syringe) loaded with therapeutic dose (250,000 SKN cells in 20 µL) was then lowered using the stereotaxic apparatus through the burr hole and into the brain to the pre-determined z-plane to deliver cells to the dorsal hippocampus. Cells were discharged over 3 min and the needle left in place another 3-min to allow cell diffusion. This procedure was carried out for both left and right hippocampi. Previously inserted titanium screws were removed.

### Canine brain donation

All procedures were performed in accordance with New South Wales and Australian law. The brains were acquired through donation with owner’s consent for research use at the time of elective euthanasia. All CCD dogs underwent brain extraction within 4 h post-mortem. Brains were bisected into hemispheres. One hemisphere was cut into 4-mm thick coronal sections, snap frozen in isopentane at − 80 °C then stored at − 80 °C. The other hemisphere was immersion fixed in 4% paraformaldehyde for 14 days before being cut into 4-mm thick coronal sections. Regions of interest were then dissected out from these thick sections, embedded into paraffin blocks.

### Immunohistochemistry

10 µm thick sections were cut from the paraffin blocks with a microtome (Thermo Electron Corporation) and mounted onto slides before being dried in an oven at 40 °C for 2 days. All sections were cleared in two 10-min rounds of xylene, and then rehydrated in successive ethanol dilutions (100%, 100%, 95%, 70%, 50%) for 3 min each. All sections were then washed in distilled water for 5 min.

For H&E staining sections were then stained in hematoxylin solution for 8 min at room temperature, followed by washing in running tap water for 5 min and then in distilled water for 2 min. Sections were then placed in 70% ethanol for 2 min, followed by 90% ethanol for 2 min. Sections were then stained in eosin solution for 1 min before being dehydrated in two changes of 95% ethanol and 2 changes of 100% ethanol for 2 min each. Finally, sections were cleared in xylene for two 5-min washes before being coverslipped in DePeX (Thermo Fisher Scientific Australia Pty Ltd, Scorseby, VIC, Australia).

For amyloid staining sections underwent antigen retrieval in 90% Formic acid for 25 min at 90 °C. Sections were then washed in distilled water for 5 min before being placed in 2% H2O2 in 50% methanol for 30 min at room temperature. Sections were then washed in Tris buffered saline with 0.05% Tween (TBST). Blocking was carried out in 10% Donkey Serum in TBST for 30 min at room temperature, before incubating sections with a bAmyloid 42 primary antibody (1:50 dilution; ab10148; Abcam, Cambridge, MA) diluted in 1% Donkey serum in TBST for 4 h at room temperature. Sections then underwent three washes in TBST for 5 min each, followed by incubation with an anti-rabbit HRP conjugated secondary antibody (1:100; ab6721; Abcam, Cambridge, MA) diluted in 1% Donkey serum in TBST for 45 min at 37 °C. Sections were then washed in TBST 3 times for 5 min each, before incubating with a DAB reagent for 30 min at room temperature, made up according to the manufacturer’s instructions (Ab64238; Abcam, Cambridge, MA). Sections were lightly counterstained with cresyl violet for 8 min and washed in tap water for 3 min before being dehydrated in successive ethanol dilutions (50%, 75%, 95%, 100%, 100%) for 2 min each, followed by two 5 min washes in xylene. Sections were finally coverslipped in DePeX (Thermo Fisher Scientific Australia Pty Ltd, Scorseby, VIC, Australia).

For tau staining, we followed the protocol in our recent publication [25]. Briefly, sections underwent antigen retrieval in 0.01 M pH 6.0 citrate buffer (Sigma-Aldrich, Castle Hill, NSW, Australia) at 95 °C for 10 min with 0.05% Tween (Sigma), followed by washing in 0.05% TBST for 5 min. Blocking was then carried out in 10% Donkey Serum in TBST for 45 min at room temperature, before incubation with an anti-p-Tau S396 antibody (Abcam, Ab109390), diluted 1:500 in 1% donkey serum in TBST. Sections were washed in TBST, and the EnVision™ G2 System/AP, Rabbit/Mouse (Permanent Red) Kit (Dako) was applied, as per manufacturer instructions. Briefly, sections were incubated in each of tubes 1 and 2 for 30 min with subsequent TBST washes, before a 10 min incubation in a 100:1 mixture of tubes 3 + 4. Following this, cells were washed in distilled water, lightly counterstained in hematoxylin, and then dehydrated in graded ethanols followed by xylene, and mounted in DPX.

Brightfield immunohistochemistry was imaged using an Olympus VS-120 Slide Scanner under a 40 × objective.

### Synaptophysin and Beta-3-Tubulin histology

Sections were cut, dried, cleared, and rehydrated as above. After washing in TBST, antigen retrieval was performed in pH 9.0 Tris–EDTA buffer for 5 min at 95 °C, before further washing in TBST. Blocking was performed with 10% donkey serum at room temperature, before overnight incubation at 4 °C with primary antibodies against synaptophysin (Abcam, ab32594), diluted at 1:300, and Beta-3-Tubulin (TUJ1) diluted to 1:1000 (BioLegend, 801201). The next day, sections were washed in TBST, then incubated at room temperature with Alexa fluorophore conjugated antibodies (1:400) for 1 h. Following further washing in TBST, cell nuclei were stained for 15 min using NucBlue for Fixed Cells (Thermo Fisher), washed in TBST, and mounted using Faramount mounting media (Dako). Antibody negative control sections were also examined by swapping synaptophysin for a rabbit IgG isotype control (Ab37415, Abcam), with all other aspects of the labelling, imaging, and analysis performed identically, to confirm binding specificity.

Sections were imaged using a Nikon C2 Confocal microscope, under 4× and 40× objectives to identify and capture regions of interest.

### Canine sand maze

We used a modified version of our published protocol [35] to carry out this appetitive, dry-land test of spatial learning and memory. Briefly, the dog is familiarised with the sand arena (6 m radius with 4 large orientating signs) and then must show food-driven motivation, assessed by voluntarily walking towards and eating a sign-posted food treat. Then the dog enters the learning trial phase of 8 trials (instead of 2 × 8 = 16 trials as originally designed because of fatigue in our older dogs): these comprise a signposted food treat trial in a specific arena location alternating with an unflagged trial where the treat is hidden in the same location below the sand. On each trial the dog enters the arena from a new door. The four unflagged trials comprise the four learning trials. The dog then leaves the arena and room for 90 min to rest, and returns for a single 90-s probe trial to determine whether the dog navigates to the learnt spatial location where there is no longer a food treat (to control for potential olfactory-based navigation a dummy or foil food reward is buried one-quarter arena rotation away). Time to reward quadrant, reward zone and action (digging up food) is captured by video and analysed automatically using customised TrackMate Pro (TrackMate, Australia) software. CSM was performed prior to treatment and then at the 3-month and 6-month follow up timepoints.

### Fitbark™ wearable technology

Fitbark 2 accelerometer device (FitBark Inc, USA) was attached to the dog’s collar before treatment and continued to be worn at least until the 3-month follow (primary endpoint). The dog’s carer was instructed to download the FitBark App in order to sync device data, that was then accessible by the research team through the FitBark Beta web-based platform. Fitbark provides a number of proprietary activity-based measures, of which Play Time (Hours) per day was used, defined by the supplier as high energy activity such as when the dog is walking fast or running.

### Function cloud wearable technology

Function Cloud was developed and validated internally for the tracking of human movement around the home and deployed here for the assessment of canine home-based movement. In human validation studies, accuracy was assessed compared to a gold-standard laser positioning grid system and spatiotemporal error found to be less than 50 cm/s.

In our canine patients, dogs wore a beacon signal device on their collar, whilst wireless anchor stations were mounted onto the walls of the home (positioning optimised based on house layout). After spatial calibration, spatial temporal co-ordinates relative to a reference point in the home were collected continuously before treatment (baseline) and for at least 3-months post treatment until the primary endpoint.

Function Cloud’s IT architecture comprised a wireless beacon worn by the subject (dog), 4–6 fixed wireless anchor stations positioned around the home and a wireless access point connected to the anchors to relay readings of each anchor back to a central in-home server. Connectivity from the in-home server to the web-based Function Cloud platform is managed through a gateway (modem/router). Access to the Function Cloud platform by the study team is through a secure authentication layer that requires a user name and secure password.

On demand analysis proceeded by extraction of spatiotemporal data, creation of spatiotemporal colour-coded density maps of subject movement over a particular time window, and image processing based on colour thresholding and percent areal fraction using ImageJ.

### Rodent study

#### SKN donor cells

The same manufacture methods as for the canine trial were used (see above). For this study, all transplants were from the same cell line derived from a domestic dog. This was a community-based dog that underwent routine surgery at Struggletown Veterinary Hospital (Randwick, NSW, Australia) and who had excess waste tissue opportunistically harvested and donated by consenting owners. The use of canine tissue was approved by the Animal Care and Ethics Committee of the University of New South Wales.

#### Preparation of SKN donor cells

SKNs were cryopreserved using a standardized slow freezing procedure [36]. Four to seven days prior to surgery, a frozen SKN sample was thawed, plated onto a T25 flask and subjected to standard propagation conditions consisting of DMEM-F12 (3:1 Invitrogen, Carlsbad, CA), which contained 20 ng/mL EGF (BD Bioscience), 40 ng/mL bFGF (Invitrogen), and 2% B27 (Gibco) at 37 °C/5% CO2. On the day of the surgery, a single cell suspension was prepared for bilateral transplantation. Approximately 250,000 SKNs were suspended in 7 µL of vehicle solution consisting of DMEM-F12 media. Cells were transferred to the surgical facilities immediately. In this experiment, the SKNs used were from Passages 2 and 3.

### Behavioural apparatus/testing procedure

The object and place memory recognition apparatus were as described previously [37]. On Days 1–5, animals underwent the object and place recognition memory tasks. Briefly, all animals were familiarised with the chamber before exposure to the place (a change in location) or the object (a change in object) memory task. Both tasks and objects were counterbalanced amongst subjects. Following this, cell or sham transplantation surgery was conducted. Animals were allowed to recover for 5 days. Running wheels were placed in their home cages for 2 weeks following surgery. Running wheels were then removed at the end of 2 weeks, and all animals were kept in standard housing conditions for an additional 6 weeks. Animals then underwent a follow-up behavioural test of object and place recognition memory as previously described. All locations and objects were counterbalanced. At the end of the behavioural paradigms, all animals were sacrificed, brains extracted and stored in the same manner as described in [37].

### Surgery and transplant

After the first behavioural testing, animals received a cell transplant or sham transplant directed towards the CA1 of the hippocampus. Before surgery, rats were anesthetized with isoflurane (Laser Animal Health) mixed with oxygen gas inhalation for 5 min. Anesthetized rats were then mounted on a stereotaxic apparatus (Model 900, Kopf Instrument, Tujunga, CA, USA), and the incisor bar was maintained at approximately 3.3 mm below horizontal to achieve a flat skull position. Prior to incision, 0.05–0.1 mL of local anaesthetic bupivacaine (100 mg/20 mL) was injected along the incision line. Immunosuppression was not employed as previous allogenic or xenogenic stem cell therapy experiments did not reveal graft rejection in rodents [38–41]. The operative site was prepared by shaving the overlying fur and cleaning the area with betadine. A small incision was made in the scalp, and the skull exposed and cleaned before a small hole was drilled directly above the desired injection site, taking care not to puncture the dura.

A 25µL Hamilton gastight syringe (Hamilton, Reno, NV, USA) containing 250,000 SKN cells in 7uL of DMEM-F12 media was mounted and secured on the stereotaxic apparatus attached to a 26-gauge needle. Bilateral intrahippocampal injection proceeded at the stereotaxic co-ordinates of A/P − 3.72 mm, M/L ± 3.00 mm, D/V 2.8 mm (Fig. 1.5). The needle tip was inserted into the site and left in place for three minutes. The cell load was slowly injected over five minutes and the needle was left in place for an additional three minutes before removal to allow for diffusion and to reduce spread up the injection tract. The process was repeated on the contralateral side. The drill hole in the skull was sealed with bone wax, and the incision site sutured closed.

Rodents receiving sham surgery received an equivalent volume of acellular vehicle solution, consisting of DMEM-F12 media.

Immediately after surgery, rats received intramuscular injections of 0.15 mL of a 300 mg/mL solution of procaine penicillin and 0.1 mL of 100 mg/mL cephazolin sodium, and subcutaneous injections of 5 mg/kg carprofen. In addition, the non-steroidal anti-inflammatory analgesic carprofen (Rimadyl, 5 mg/kg) was injected subcutaneously to provide post-operative analgesia. Rats were moved to a heated-mat recovery box for 24 h, and then transferred to their normal cage when mobile and voluntarily drinking. They were allowed 5 days to recover from surgery, during which time they were weighed and observed daily.

At the conclusion of this behavioural experimental protocol, animals were sacrificed.

### Histology

For sacrifice rats were deeply anesthetized with sodium pentobarbital (100 mg/kg, i.p.) and perfused transcardially with 100 mL of 0.9% saline, containing 1% sodium nitrite and heparin (5000 i.u./mL), followed by 400 mL of 4% paraformaldehyde in 0.1 M phosphate buffer (PB), pH 7.4. Brains were postfixed for 1 h in the same fixative and placed in 20% sucrose solution overnight. Brains were blocked using a matrix aligned to the atlas of Paxinos and Watson (1997), and 40 μm coronal sections were cut using a cryostat (Microm HM560, Microm International). Seven serially adjacent sets of sections were obtained from each brain and stored in 0.1% sodium azide in 0.1 M PBS, pH 7.2 and prepared for the following brightfield, fluorescent and confocal analyses: *Lamin* (donor SKN cell marker), *Lamin* + *ßIII-tubulin* (neuronal differentiation), and *synaptophysin* (presynaptic density marker).

*Lamin* One series of sections through the hippocampus was selected from each rat to reveal donor lamin-positive cells using peroxidase immunohistochemistry. Free- floating sections were washed repeatedly in 0.1 M PB, pH 7.4, followed by two 30 min washes in 50% ethanol, the second of which contained 3% H2O2, and were then incubated in 5% normal horse serum (NHS) in PB, pH 7.4, for 30 min. Sections were then incubated in rabbit antiserum against Lamin (1:500; Santa Cruz Biotechnologies) diluted in 0.1 M PB, pH 7.4, containing 2% NHS and 0.2% Triton X-100 (PBT-X) for 48 h at 4 °C, with gentle agitation. After washing off unbound primary antibodies, sections were incubated overnight at room temperature in biotinylated donkey anti-rabbit IgG (1:1000; Jackson Immunoresearch Laboratories) diluted in 2% NHS PBT-X. After washing off unbound secondary antibody, sections were incubated for 2 h at room temperature in ABC reagent (Vector Elite kit: 6 μl/mL avidin and 6 μl/mL biotin; Vector Laboratories). Immunoreactive (IR) nuclei labelled for lamin were generated by glucose oxidase to reveal a brown reaction product. To do this, sections were washed in PB, followed by 0.1 M acetate buffer, pH 6.0, and then incubated for 15 min in 0.1 M acetate buffer, pH 6.0, containing 2% nickel sulfate, 0.025% 3,3-diaminobenzidine, 0.004% ammonium chloride, and 0.02% D-glucose. The peroxidase reaction was started by adding 0.2 μl/mL glucose oxidase and stopped using acetate buffer, pH 6.0. Brain sections were then washed in PB, mounted onto gelatin-treated slides, dehydrated, cleared in histolene, and coverslipped with DePeX.

*Synaptophysin* One set of section series was used to assess synaptophysin density. Free-floating sections were washed repeatedly in 0.1 M PB, pH 7.4, followed by a 30 min wash in sodium borohydryde at room temperature and 30 min in citrate buffer, pH 6 (Sodium citrate, 10 mM) at 80 °C. Sections were then incubated in a blocking buffer 3% BSA and 0.25% Triton-X in PB, pH 7.4, for 30 min at 80 °C. Following this, sections were incubated in mouse anti-*synaptophysin* (1:1000; Millipore). This primary antibody was diluted in 0.1 M PB, pH 7.4, containing 3% BSA and 0.25% Triton X-100, and incubations were for 30 min at 37 °C. After washing off unbound primary antibodies, sections were incubated for 30 min at 37 °C in donkey anti-mouse IgG (1:500; Invitrogen) diluted in 0.1 M PB, pH 7.4, containing 3% BSA and 0.25% Triton X-100 (PBT-X). Sections were washed and mounted.

*ßIII-tubulin/Lamin* A third series of sections through the hippocampus was stained for the neuronal marker *ßIII-tubulin*. Free-floating sections were washed repeatedly in 0.1 M PBS, pH 7.2, followed by 4 h incubation in PBS, pH 7.2, containing 10% NHS and 0.5% Triton X-100. Sections were then incubated for 48 h at room temperature in mouse anti-neuronal nuclei (1:5000; *ßIII-tubulin*; Chemicon) and rabbit anti-lamin nuclei (1:1000; lamin; Santa Cruz Technologies), diluted in 0.1 M PBS, pH 7.2, containing 0.1% sodium azide, 2% NHS and 0.2% Triton X-100. Sections were then incubated for 4 h at room temperature in anti-fluorescent sheep anti-mouse and donkey anti-rabbit (1:500; Jackson Immunoresearch Laboratories) diluted in 2% NHS and 0.2% Triton X-100. Sections were washed and mounted.

### Hippocampal slice electrophysiology

*Viable brain slice preparation* One rat that had baseline behavioural testing at 20 months of age and a hippocampal surgical transplant of genetically pZs-Green1-labelled (gift of Prof C. Weickert) SKNs was used. The rat was sacrificed under deep anaesthesia by isoflurane inhalation (4% in air) 10 weeks after transplant. The brain was rapidly removed and cut using a vibratome (Leica Microsystems VT1200S, Germany) in ice-cold oxygenated sucrose buffer containing (in mM): 241 sucrose, 28 NaHCO_3_, 11 glucose, 1.4 NaH_2_PO_4_, 3.3 KCl, 0.2 CaCl_2_, 7 MgCl_2_. Horizontal brain slices (300 µm thick) containing the hippocampus were sampled and maintained at 33ºC in a submerged chamber containing physiological saline with composition (in mM): 126 NaCl, 2.5 KCl, 1.4 NaH_2_PO_4_, 1.2 MgCl_2_, 2.4 CaCl_2_, 11 glucose and 25 NaHCO_3_, and equilibrated with 95% O_2_ and 5% CO_2_.

*Electrophysiological recording* After equilibration for 1 h, slices were transferred to a recording chamber and visualized under an upright microscope (BX50WI, Olympus, Shinjuku, Japan) using differential interference contrast (DIC) Dodt tube optics, and superfused continuously (2 mL /min) with oxygenated physiological saline at 33ºC. Whole-cell patch-clamp recordings were made using electrodes (2–5 MΩ) containing internal solution (in mM): 115 K gluconate, 20 NaCl, 1 MgCl_2_, 10 HEPES, 11 EGTA, 5 Mg-ATP, and 0.33 Na-GTP, pH 7.3, osmolarity 285–290 mOsm/L. Biocytin (0.1%) was added to the internal solution for marking the sampled neurons during recording. Data acquisition was performed with a Multiclamp 700B amplifier (Molecular Devices, Sunnyvale, CA), connected to a Macintosh computer and interface ITC-18 (Instrutech, Long Island, NY). Liquid junction potentials of − 10 mV were corrected. Whole-cell currents were sampled at 5 kHz (low pass filter 2 kHz, Axograph X, Axograph, Berkeley, CA). Stock solutions of all drugs were diluted to working concentrations in the extracellular solution immediately before use and applied by continuous superfusion.

*Histology and microscopy* Immediately after whole-cell physiological recording, brain slices containing biocytin-filled neurons were fixed overnight in 4% paraformaldehyde/0.16 M phosphate buffer (PB) solution followed by placing them in 0.3% triton X-100/PB for 2 days to permeabilize cell membrane. Slices were rinsed in 0.1 M PB and then incubated in Alexa 647-conjugated Streptavidin/PB solution (1:500, Life Technologies, Grand Island, NY) for 2 h. Stained slices were rinsed, mounted onto glass slides, dried and coverslipped with Vectashield mounting medium (Vector Laboratories, Burlingame, CA). Biocytin staining was visualised and scanned with an Olympus laser confocal microscope (BX61WI) and software (FluoView FV1000 version 3.1).

### Extension study with GFP-labelled SKNs

SKNs were generated following the aforementioned methods, using adult canine skin biopsies. GFP transduction was performed at SKN passage 2 by incubating the cells in a 1:2000 dilution of a pTK945 CMV-GFP viral vector (gift from Professor H. van Praag) in growth media at 37 °C, 5% CO_2_ for 48 h. On day 3 the viral media was removed, cells were washed in three changes of PBS (Thermo Fisher Scientific Australia Pty Ltd, Scorseby, VIC, Australia). Fresh growth media was then added followed by further incubation for 72 h at 37 °C, 5% CO_2_. Following removal of this media, cells were then enzymatically released from the culture surface using TrypLE Select at 37 °C for 5 min. The cells were then washed in three changes of PBS, centrifuged at 180G for 10 min, and transferred into a microvial in PBS.

Rodents were anesthetized with 4% isoflurane for 5 min and then mounted on a stereotaxic apparatus. A small incision was made in the scalp to reveal the skull and a hand drill was used to expose the brain surface. Animals received either bilateral transplantation of 250,000 GFP transfected SKNs in 7 µL PBS (N = 2) or an equal volume of acellular PBS for sham control (N = 1). A 25 µL syringe fixed with a 26-gauge needle was secured on the stereotaxic apparatus and centered in stereotaxic coordinates A/P − 3.72 mm, M/L ± 3.00 mm, D/V 2.8 mm from the bregma, targeting the *stratum radiatum* located midway between CA1 and CA3 of the hippocampus. Following injection of the volume over a 5-min period, the needle was left in place for a further 3 min before removal, and the procedure repeated contralaterally. Rats received intramuscular injections of 0.15 mL of a 300 mg/mL solution of procaine penicillin and 0.1 mL of 100 mg/mL cephazolin sodium, and subcutaneous injections of 5 mg/kg carprofen. They were moved to a recovery box for 24 h, then transferred to their normal cage. Animals were observed and weighed as a method for assessing their health for 5 days post-operation.

At 8 weeks post-transplantation, rodents were anesthetized using 4% isoflurane and euthanized by 100 mg/kg pentobarbital through an intraperitoneal injection with a 26-gauge needle. Following brain extraction, 300 μm thick coronal tissue sections (containing the hippocampus) were cut using a vibratome and the tissue immersion fixed in 4% paraformaldehyde (Sigma-Aldrich) for 30 min, followed by three washes in PBS. Blocking of non-specific antigen binding was performed by incubation free floating brain sections in 10% donkey serum, 0.1% Triton X-100, in PBS for 10 min. Incubation with primary antibodies was then performed using mouse anti-NeuN (1:200 dilution, EMD Millipore; MAB377), or mouse anti-neurofilament (1:200 dilution, Biolegend; SMI-311R), and rabbit anti-GFP (1:500 dilution, Abcam; ab290) diluted in 1% donkey serum in PBS for 2 h at room temperature. before 3 washes in PBS for 5 min each. Sections were then washed three times in PBS followed by incubation with anti-mouse Alexa Fluor 594 (1:500 dilution, Invitrogen A21203) and anti-rabbit Alexa Fluor 488 (1:500 dilution, Invitrogen; A21206) secondary antibodies in 1% donkey serum for 1 h. Sections were then washed in PBS three times and counterstained in DAPI (1 μg/mL) for 10 min. Finally, sections were mounted onto slides using Dako mounting medium, and visualized by fluorescence confocal microscopy (Nikon C2 Plus Confocal Microscope, Leica SPE-II Confocal Microscope).

## Quantification and statistical analysis

### Canine trial

*Histological image quantitation* Images obtained were analysed in an automated fashion using a custom macro in Fiji software (National Institute of Health). Briefly, images were split into component channels, and mean intensity for each channel measured. For quantitation, raw images as obtained from the microscope were used; for visualisation, images were automatically colour-balanced channel-by-channel in Fiji.

*For values presented in SD units* This method was used when all sections (dorsal hippocampus) were prepared, processed, acquired and analysed in the one histological run and hence images had maximal comparability. Raw image values were first adjusted to background by subtraction of WM synaptophysin intensity from within the same image (i.e. internal negative control). Adjusted values were then normalised by reference to the subfield regional SD and mean of non-treated older dogs (*N* = 10) to produce a z-value (in SD units).

*For values presented in AU* This method was used when the large number of sections required multiple histological runs (all regions except dorsal hippocampus). A method was applied to allow comparability between runs. First, a single dog’s regional sections were split across the runs to serve as a reference value. Second, as above, all raw image intensity values were adjusted by subtraction of image-specific WM background. Third, a runwise correction factor was applied, calculated as the WM-adjusted intensity value of the experimental tissue/WM-adjusted value of the reference value for that run. Accordingly, all AU units are relative to the same reference dog’s values after WM correction.

*Statistical analysis* A modified intention-to-treat (mITT) analysis was pre-specified, excluding any treated patient that did not recover after surgery. This excluded one dog, Sasha, who had a serious adverse event (SAE) during surgery and subsequent euthanasia (see Additional file 1: Table ST1 for more details). Statistical analysis was by a paired T-test of the mITT sample based on change on the CCDR score. We carried out sensitivity analysis on the full ITT sample by imputation of the single excluded patient by the Last Observation Carried Forward method. For individual level analyses, we carried out T-test comparisons between before and after data.

### Rodent study

*Stereological cell counts* All unbiased stereological assessments were performed using StereoInvestigator software (MicroBrightField, Williston, VT). For the stereological evaluation of transplanted *lamin* positive cells, an optical fractionator probe was used to estimate mean cell numbers and the Cavalieri principle was used to determine volume of each brain region [42]. Guard zones were set at 10% of measured thickness with a minimum 15um optical dissector height. Contour tracing to delineate brain regions was done using a 4X objective and counting was performed using a 60X oil objective. The counting was done in every 7th section (40 um coronal sections) using *n* = 8 animals per group. A 25 × 25 counting frame was used in all stereological evaluations and all counts were performed in both brain hemispheres. For a stereological evaluation of *lamin* and *ßIII-tubulin*, a 200 × 200 μm sampling grid was used to assess the entire hippocampus.

*Optical density quantification* Approximately 12–15 sections per animal were immunofluorescently labelled in parallel for the presynaptic vesicle protein *synaptophysin* following standard protocols. Slides were coded and images captured using a confocal microscope with identical scan settings. Grayscale images were inverted and optical density was quantified using ImageJ software. Briefly, regions of interest (ROIs) including the stratum radiatum of CA1 and the dentate gyrus were defined in each image. Average pixel intensity was measured for each ROI. Pixel intensity measurements from white matter within the same section (corpus callosum) were also taken to provided background levels and subtracted from ROI values to generate background-corrected values used in all analyses.

*Statistical analysis:* Data were analysed by *t* test or ANOVA for multiple comparisons unless otherwise indicated and considered significant at *p* < 0.05.

## Results

### Clinically meaningful response

**Fig. 1:**
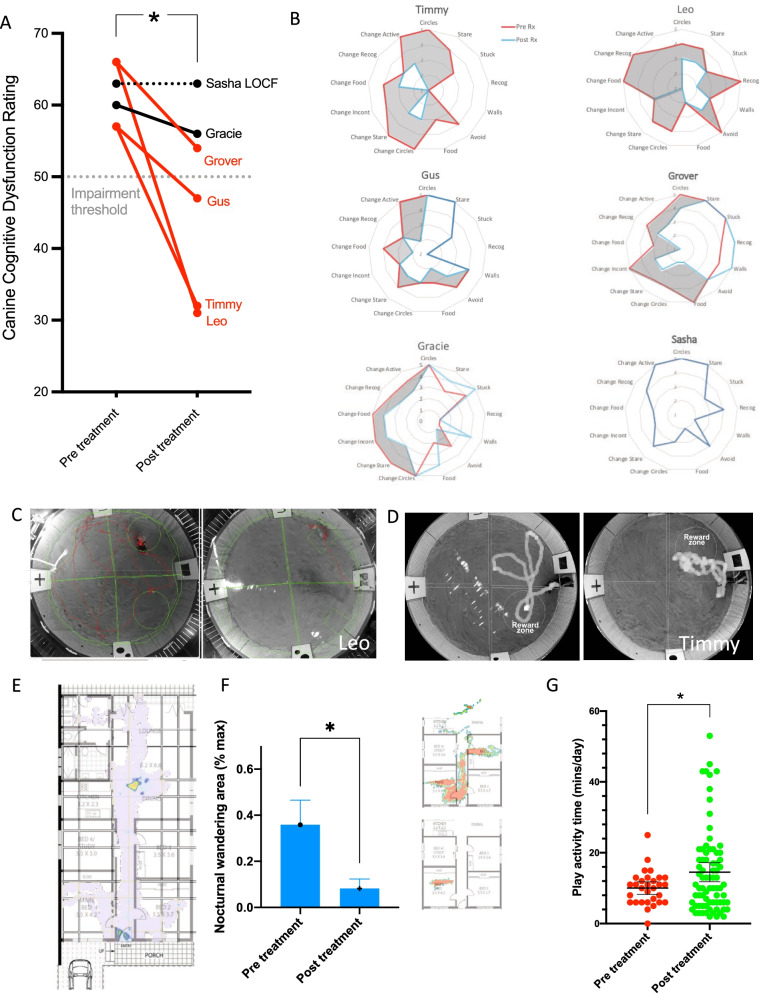
SKN cell therapy is effective at treating Canine Cognitive Dysfunction (CCD). **A** Primary endpoint CCDR score (global clinical severity) as rated for each patient before after treatment shows a significant decrease over time, where lower scores are better. Sasha follow-up score imputed by last observation carried forward (LOCF) because of a serious adverse event and euthanasia. Red lines show 4/5 patients categorically improved beyond RCI of CCDR (> 5 points). Modified ITT analysis: Paired *T* test = 3.06, *df* = 4, *p* = 0.038. **B** Radar graphs of individual CCDR items for each patient, where higher scores indicate greater severity of abnormal behaviour or higher rate of decline for change items. Grey zone represents extent of global clinical improvement. **C** Canine Sand Maze paths for Leo and **D** Timmy final learning trial, before (left) and after treatment (right). These show that after treatment more time was spent in reward zone and dogs traversed a shorter path length, suggestive of improved encoding of spatial memory. **E** Function Cloud spatial map of home-based movement for Grover over a daytime hour (pre-treatment). Hot spot in the dining room shows zone of frequent repetitive circling behaviour. **F** Grover’s spatial maps overnight (2–3 am) were used to calculate daily nocturnal wandering area, which showed a significant decrease pre-treatment (1-week) compared to post- treatment (final week 12 follow-up), consistent with carer reports (*T* test = 2.89, *df* = 6, *p* = 0.028, figure shows average ± SEM). Inserts show exemplar maps for one pre- (top) and post- treatment night (lower). **G** Fitbark™ wearable accelerometer technology was used to assess daily playful activity and showed a significant improvement over the treatment period, consistent with Gus’ carer reports (*T* test = 2.006, *df* = 105, *p* = 0.047, figure shows average ± 95%CI, pre-treatment 30 days vs. post treatment 77 days)

Over an 8-year period, we recruited *N* = 8 and treated *N* = 6 community-dwelling CCD dogs in the DOGS + CELLS trial (University of Sydney Animal Ethics Committee #2019/1612). Modified intention-to-treat ITT analysis (*N* = 5) found a significant improvement on the trial’s primary endpoint: 3-month change on our validated [16] and independently recommended [43] *Canine Cognitive Dysfunction Rating* scale (CCDR). Global clinical CCDR scores improved by 28% and an average 17-points (see Fig. 1A and B for item by item radar plots).

Four-out-of-five (80%) canine patients responded therapeutically, defined as improvement beyond the Reliable Change Index (RCI) of the CCDR (≥ 5 points), whilst three patients (60%) dropped below CCD diagnostic threshold (50 out of maximum 86), and two patients (33%) underwent a full syndromal remission, evident by scores improving by > 43% (see before-after videos in Additional file 4, 5). Sensitivity analysis of the full ITT sample (*N* = 6), by imputation, conserved the positive trial result (Paired *T* test = 2.65, *df* = 5, *p* = 0.045).

Whilst our sample was limited to a relatively small number and in keeping with other large animal cell therapy studies in parkinsonian models [44], clinical outcomes appear to have been decisive. Autologous SKN cell therapy can effectively treat and even reverse CCD in older dogs. No dog clinically deteriorated during the 3-month follow-up period despite a history of decline being required for initial diagnosis. Moreover, quality of life returned in a majority of patients, and in two cases this persisted for almost 2 years.

### Objective behavioural measures

An open-label design was used in this veterinary trial for the same reason it is commonly employed in first-in-human cell therapy trials [45]: CCD is a progressive and terminal disorder and surgical delivery encompasses some risk; it was considered ethically inappropriate to employ an inert control arm. To guard against expectancy bias, we developed and implemented a series of exploratory objective behavioural measures during the 8-year course of the trial.

Initially, we used the Canine Sand Maze (CSM) [35], an appetitive, dry-land spatial learning and memory test modelled on the standard Morris Water Maze used in rodents. In Leo, we found significant within-animal improvement between pre- and post- treatment tests (Additional file 1: Figure S10), including on the final learning trial (Fig. 1C), whilst for Timmy there was also better final learning trial performance (Fig. 1D) but this was non-significant across trials (Additional file 1: Figure S11).

A limitation of the CSM was lack of practical administration on all animals, and so we changed to use of wearable devices to capture high-fidelity data in a naturalistic and non-intrusive manner. Function Cloud was developed and validated for mapping movement around the home. For Grover, this was sensitive to his main clinical sign, repetitive circling (Fig. 1E), and verified the CCDR report of improved wandering (Fig. 1F). This pattern of reduced in-home wandering was replicated in Gus (Additional file 1: Figures S12, S13) and in addition, a commercial device (Fitbark™, USA) was used to measure playful physical activity outside the home; this also verified Gus’ main area of clinical improvement on the CCDR (increased activity level) (Fig. 1G). Whilst these wearable technologies were supportive of the primary clinical endpoint, their progressive implementation during the trial was a limitation because a complete dataset was not available for each treated veterinary patient.

### Cellular safety

**Fig. 2:**
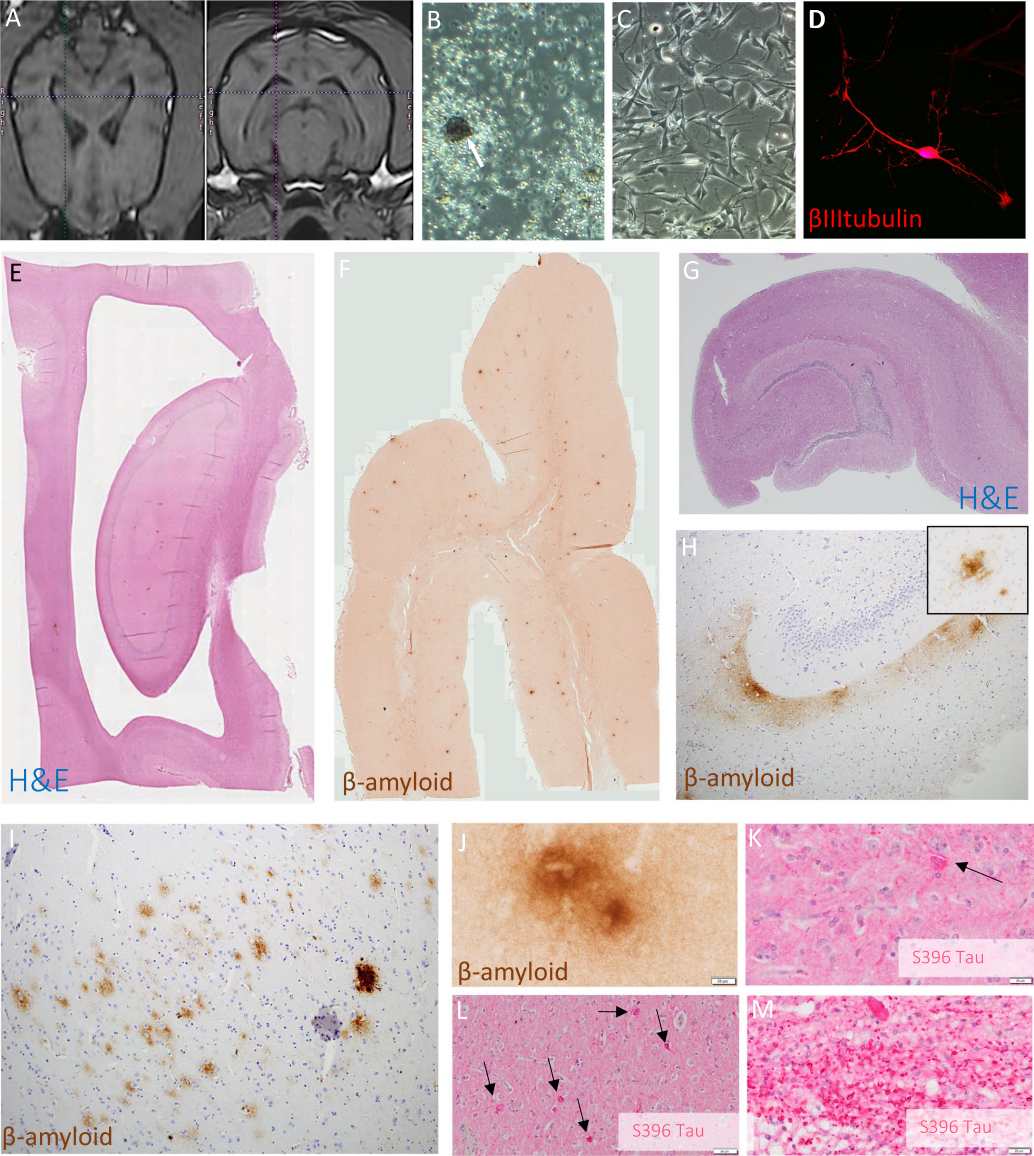
Patient-specific SKN cell therapy is safe over long term and does not impact AD pathology. **A** Orthogonal MRI slices show targeting of SKN microinjection in canine dorsal hippocampus (Timmy; see Additional file 1: Materials for panel with targeting for all canine patients). **B** Characteristic cell morphology of SKNs at neurosphere suspension phase (arrow identifies a neurosphere from Grover cell line), and **C** adherent expansion phase at which point cells are harvested and engrafted (Timmy cell line). **D** SKN cell following 21 days in vitro neuronal differentiation in Leo cell line showing typical neuronal morphology and marker expression (*βIII tubulin;* see Additional file 1: Fig. S14 for *neurofilament*)*.* **E** H&E image low magnification of hippocampus shows normal cytoarchitecture in Grover at *post mortem* (4-months follow up). **F** β-amyloid Alzheimer disease plaque pathology in Grover frontal lobe low magnification. **G** Normal hippocampal H&E cell histology in Timmy (20-months follow-up). H) β-amyloid pathology in Timmy hippocampus, with insert showing high magnification of a diffuse plaque, and in I) occipital cortex, a combination of diffuse and dense core plaques. **J** High magnification of β-amyloid diffuse plaque in Grover hippocampus (CA3). K) S396-hyperphosphorylated tau positive inclusions in Grover anterior cingulate and **L** frontal cortex. **M** S396-tau positive tauopathy in Grover thalamic white matter bundles cut in cross-section. Grover had the highest tauopathy burden of any dog in our Canine Brain Bank and fastest rate of clinical decline prior to treatment (see Additional file 1: Materials).

Long-term cellular safety was assessed since adverse effects from donor cells or tissue engraftment can take years to manifest [46]. Figure 2A shows an example of patient-specific anatomical targeting of the dorsal canine hippocampus using MRI (Additional file 1: Figure S2 for targeting for each patient)**,** whilst Fig. 2B–D displays patient-specific SKN cell production at different in vitro stages. All therapeutic cells passed release criteria prior to implantation (see Additional file 2: Supplementary Material Protocols).

There was a surgical SAE in our first treated patient (Sasha), who suffered an intraoperative bleed, post-operative complications and euthanasia. Pathologist review (MB) determined that the likely root cause was an undiagnosed bleeding disorder, and this triggered trial suspension, protocol review and tightening of recruitment criteria and screening. Following this, there were no safety concerns for any of the subsequent *n* = 5 treated dogs based on regular clinical reviews and laboratory tests.

Histology of the hippocampus in three canine patients showed that there was no evidence of abnormal cell proliferation, dysplasia or tumour (Fig. 2E, G), and canine SKNs are therefore likely to be safe for intracerebral therapeutic applications.

Classic human AD pathology measures were also examined. As shown in Fig. 2F, H, I, J, Timmy and Grover had β-amyloid deposition throughout the cortex and hippocampus, mainly in the form of diffuse plaques, as well as tauopathy in the Papez circuit as we have previously reported [25] (Fig. 2K–M). Indeed, Grover had the highest tau pathology of any CCD dog in the wider Canine Brain Bank of *untreated* aged animals (*N* = 12, Additional file 1: Figure S3), consistent with his precipitous rate of clinical decline prior to treatment. Therapeutic clinical outcomes observed here were therefore in the context of progressive and multifactorial AD pathology, suggestive that disease modification is not a likely mechanism.

### Synaptic regeneration

**Fig. 3:**
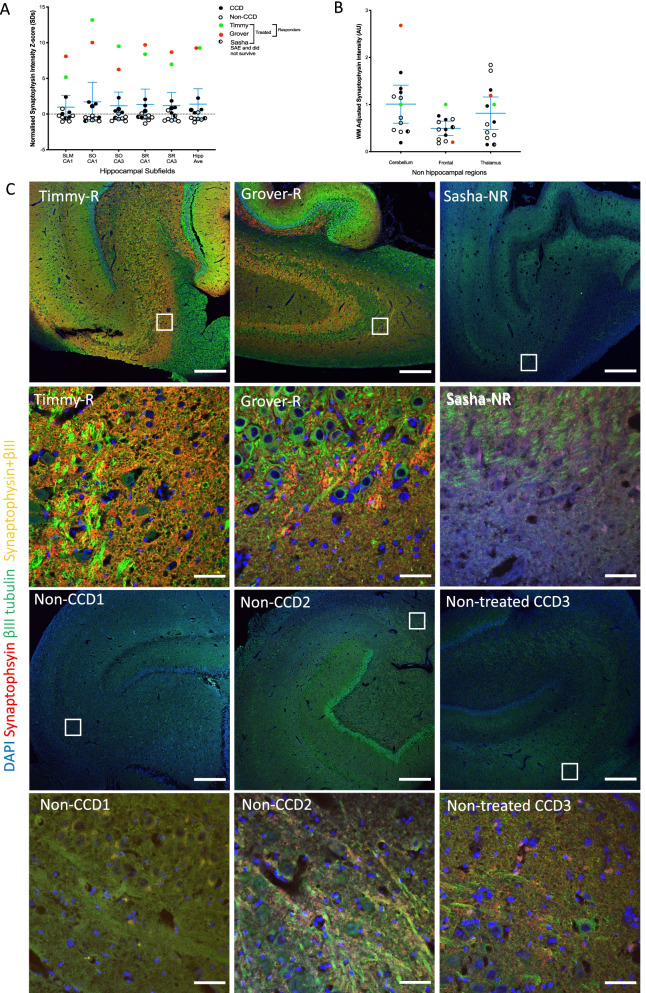
SKN cell therapy triggers supraphysiological hippocampal synaptogenesis. **A** Chart showing synaptic density in hippocampus in therapeutic responders is specifically and markedly increased, 5–13 SDs higher (average ~ 9SD) than in non-treated aged animals (*n* = 10). Charts shows average ± 95%CI for untreated dogs. **B** No significance difference was observed in non-hippocampal areas. **C** Exemplar images from the CA3 *stratum oriens* subfield (SO, high and low magnification). Responders showed a remarkably high density of neurons positive for immature marker *βIII tubulin (green)* that are absent in non-treated CCD older dogs, as well as intense positivity for presynaptic *synaptophysin (red,* and co-labelled immature neuronal presynaptic punctae (yellow). Panels for CA1, CA3 stratum radiatum and dentate gyrus in Additional file 1: Figures S7–9

Next, we analysed the intensity and distribution of presynaptic marker *synaptophysin* and early neuronal marker *ßIII tubulin* to evaluate potential for regeneration (see Additional file 1: Figure S6 for IgG controls). Strikingly, we found that synaptic markers in our two therapeutic responders (Timmy and Grover) were ~ 9 standard deviations (SDs) higher than in untreated CCD dogs or even non-CCD older dogs (average hippocampal intensity *T* = 12.6, *df* = 10, *p* < 0.0001; Fig. 3A,C). These effects were specific to the hippocampus, the therapeutic target, because we found no systematic difference in non-hippocampal areas (Fig. 3B). A similar patterns of results (4–5SD increase) was found for *ßIII tubulin*, a marker of immature neurons (Fig. 3C and Additional file 1: Figure S4–5).

### Rodent model of canine SKN cell engraftment

**Fig. 4:**
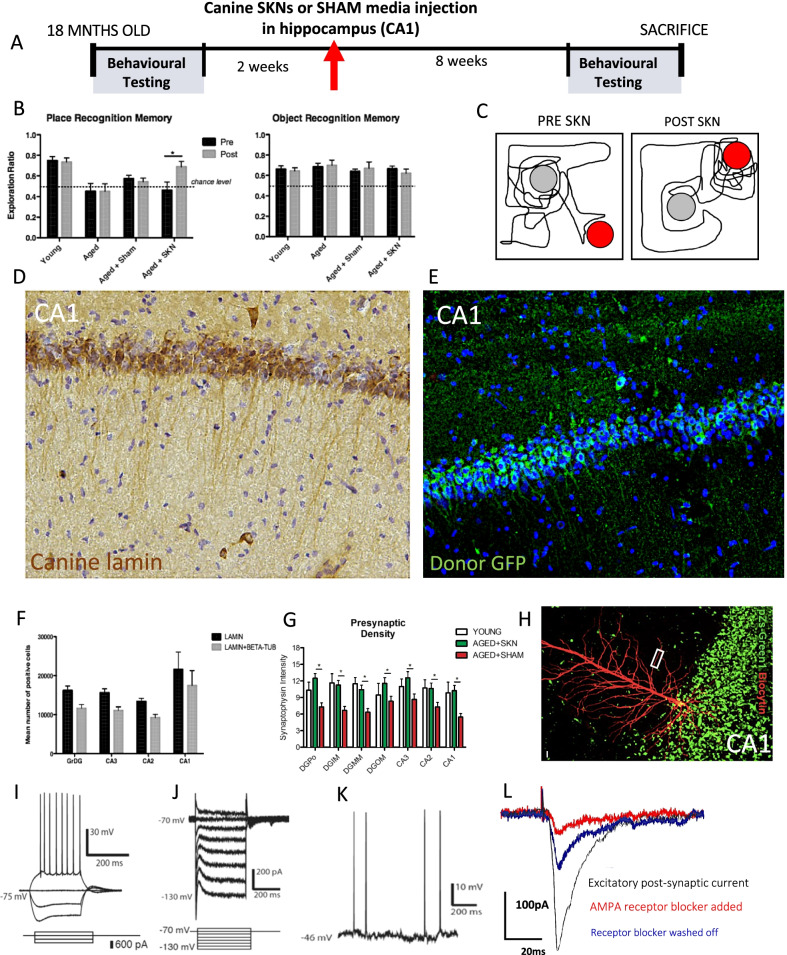
SKN neuronal engraftment and synaptic rescue in rodent model of age-related memory dysfunction. **A** Experimental paradigm. **B** Canine SKN cell transplantation rescues hippocampally-dependent Place Recognition Memory (PRM) back to young animal level (Time X Group F1,14 = 6.934, *p* = 0.02; left; see Additional file 1: Figure S24 for individual animal data) and has no effect on intact ORM (right). Untreated young and old animal data from our prior report [37]. **C** Exemplar trace of rodent exploration of familiar object in a novel position during PRM before and after treatment. **D** Canine-specific *lamin* marker of donor cells (see Additional file 1: Figures S15–17 for controls) shows extensive survival and engraftment of canine SKN cells in CA1 in main experiment (*N* = 16; see Additional file 1: Figure S18 for engraftment in other hippocampal areas). **E** Extension study (*N* = 2) with GFP-genetically labelled donor cells shows engraftment in CA1 with anatomically-correct cell bodies and penetrating dendrites; see Additional file 1: Figure S19 for co-expression with mature neuronal *NeuN* and *neurofilament*. **F** Stereological counts of donor cells throughout hippocampus marked by canine *lamin,* and *Lamin* + *βIII tubulin* co-expression indicative of in vivo neuronal differentiation (*N* = 16; see Additional file 1: Figure S19 for confocal fluorescent images). **G** *Synaptophysin* was restored to young animal levels throughout hippocampus compared to media vehicle (*N* = 16). **H** Donor cells genetically labelled with pZs-Green1 (green), allowing cell identification in a fresh hippocampal slice preparation, here filled with biocytin (red) to reveal pyramidal morphology including dendritic spines on primary dendrites (z-stack image, see Additional file 1: Figures S21–23 for orthogonal confocal images and high magnification of secondary dendrite with intense decoration by synaptic spines). **I** In a hippocampal slice preparation, SKN donor cells (*k* = 3) displayed elicited action potentials in CA1 under current clamp and **J** voltage clamp conditions. **K** Spontaneous action potentials in SKN donor cell in the dentate gyrus. **L** Excitatory post-synaptic currents in CA1 (same cell as in **H**) following stimulation of distal Schaffer collateral fibres, abolishable by AMPA-receptor blockade, indicating that canine SKN cells can integrate into physiological neuronal circuits. *D*, *E* and *H* are from independent animals

Use of genetically non-modified autologous cells meant there was no definitive donor cell marker for assessment of biodistribution. Due to this limitation we could also not rule out that increases in synaptic and neuronal markers may have been due to paracrine effects alone [47], despite their magnitude and durability over 1–2 years making this unlikely. To directly address this, we modelled our treatment in *N* = 16 aged rats where place recognition memory, but not object recognition memory, is severely impaired, linked to synaptic depletion in hippocampal subfields [37]. We randomly treated animals with 250,000 canine SKNs microinjected into each hippocampus (*n* = 8) in comparison to media-only sham controls (*n* = 8; Fig. 4A). Choice of cellular dose for both this rodent study and the canine trial was based on precedent in preclinical animal studies of neurodegeneration that have used different types of therapeutic cells [48].

As in our canine trial, SKN treatment significantly improved place memory, normalising performance to the level of young rats (Fig. 4B and C; Additional file 1: Figure S24). This was accompanied by a high degree of donor cell survival at 10-weeks (27% based on canine-specific lamin *marker*; see Additional file 1: Figures S15–17 for control studies), cells migrating throughout the hippocampus and maximal engraftment seen in the CA1 subfield (Fig. 4D, E and Additional file 1: Figure S19). A high proportion of donor cells differentiated into neurons (73% co-expressed neuronal *βIII tubulin*; Fig. 4F) and, in an extension study with GFP-labelled donor cells (*n* = 2), co-expression of mature neuronal markers *NeuN* and *Neurofilament* was ubiquitous (Additional file 1: Figure S20). There was however no assessment of biodistribution outside of the brain, an issue that needs to be addressed in future studies.

Age-related decline in synaptic density was also completely rescued (Fig. 4G), consistent with results from our canine trial. Electrophysiological recordings from *k* = 3 donor cells in a hippocampal slice preparation produced elicited (Fig. 4I, J) and spontaneous (Fig. 4K) action potentials, evidence of neuronal function in situ. Further, upon stimulation of distal Schaffer collateral afferents, we recorded excitatory post synaptic currents from a donor cell in CA1, abolished by treatment with AMPA-receptor blocker cyanquixaline (CNQX) (Fig. 4L). This cell showed adoption of classical pyramidal cell cytoarchitecture (Fig. 4H and Additional file 1: Fig. 23; Additional File 6) and numerous synaptic spines (Additional file 1: Figures S21, 22).

Rodent modelling of age-related synaptic loss and memory dysfunction therefore provided strong evidence that canine SKN donor cells can survive and neuronally differentiate in vivo, are effective at rescuing age-related memory dysfunction, and, in-principle, are capable of integration into host mnemonic circuits.

## Discussion

Altogether, these findings signal an initial step towards an all new therapeutic approach to AD. To date, there has been no attempt to restore the lost neurons and synapses that is the biological basis of Alzheimer dementia symptoms. Congruent findings of neuronal and synaptic restoration, and cognitive recovery, in dogs and rodents suggest such an approach is rational and the basis for further research.

Caution is however required because of the noted limitations in the canine study. This was a small, open-label, non-controlled veterinary trial and secondary outcomes using objective behavioural measures, whilst convergent, were not collected using the same technology across all animals. In addition, we found only indicative evidence of neurorestoration in those canine patients who came to *post mortem,* in the form of strikingly higher numbers of new hippocampal neurons and synapses, because a definitive donor cell marker was not available. This was addressed by modelling of the therapeutic process, using the same canine SKNs in aged rodents that develop similar synaptic and mnemonic deficits, but do not develop AD pathology. As in the canine trial, we found profound increases in synaptic density, normalization of impaired hippocampal-dependent memory, and importantly, high levels of donor cell survival, in situ neurodifferentiation, adoption of correct CA1 anatomy, and hippocamapal circuit integration. The positive clinical and neurobiological results of the canine trial are therefore consistent with observations from a xenograft rodent model using the same donor cells.

Our results add to prior studies of cell-based treatment of AD in rodent models. MSCs transplants in AD mice models have generally found no cell survival beyond 4–6 weeks, and a complex pattern of paracrine mechanisms converging on modulation of inflammation and microglial responses [13, 53]. By contrast, hippocampal injection of 2 × 10^5^ human IPS cells induced down a neuronal lineage found donor cells persisted for at least 45 days, differentiated into cholinergic and GABAergic phenotypes, and improved spatial memory deficits [54]. However, our study is the first to trial an autologous cell therapy in a large-animal model of AD with a dementia-like syndrome, a model with clinical, pathological, neuroanatomical and genetic similarities to the human condition. Given dogs can also predict human pharmacological response to new AD drugs, whilst rodents can not [49, 50], and we found persistence of neuroregenerative and clinical improvements for up to 2-years post treatment, there is more confidence about potential for treating human AD than on the basis of rodent data alone. That said, CCD is best considered a model of a *mild* AD dementia-like syndrome because phosphorylated tauopathy rarely expresses the human late-stage neurofibrillary tangle marker (AT8) [25], and hippocampal atrophy [51] does not appear to be due to neuronal loss above and beyond advanced age [52].

Human trial will therefore need to carefully select correspondent early stage AD patients. Starting dose will also need careful consideration, because in this veterinary trial we did not compare different therapeutic doses. Given SKN cell therapy also had no observable impact on upstream AD pathology, the net clinical effect may therefore be to ‘buy time’; a period during which cognitive circuits can resume operation and support higher-order function. In some of our canine patients this amounted to years of regained quality of life, of major personal significance to our dogs’ carers and of major clinical significance if translated to humans.

## Conclusions

With further research, it may be possible to advance a new class of restorative cell therapy for AD targeting neuronal and synaptic loss in the hippocampus founded upon neuroprecursors derived from a patient’s own skin.

## Supplementary Information

**Additional file 1.** Supplementary Materials.

**Additional file 2.** Supplementary Protocols.

**Additional file 3.** Canine neurosurgery movie [Timmy surgery.mov].

**Additional file 4.** Leo before and after movie [Leo-Before+After.mov].

**Additional file 5.** Timmy before and after movie [Timmy-Before+After.mov].

**Additional file 6.** Canine donor SKN cell in rat CA1 (z-stack fly through) [Canine SKN engrafted in rat CA1 flythrough.mov].

## Data Availability

The datasets used and/or analysed during the current study are available from the corresponding author on reasonable request.
