# Supplementary material for: Autologous skin-derived neural precursor cell therapy reverses canine Alzheimer dementia-like syndrome in a proof of concept veterinary trial
Source: Stem Cell Res Ther. 2022 Jun 17;13:261. doi: 10.1186/s13287-022-02933-w (PMC9205057; doi:10.1186/s13287-022-02933-w)

Autologous skin-derived neural precursor cell therapy reverses canine Alzheimer dementia-like syndrome in a proof of concept veterinary trial

SUPPLEMENTARY MATERIALS

CANINE METHODS

Protocol History

As this veterinary trial spanned an extended period (2012-2020), several protocol refinements and amendments were carried out during the course of the study. However, three essential features were unchanged: the diagnosis (CCD diagnosis based on CCDR threshold >=50 after exclusion of biological causes), the cell therapy (patient-specific SKN cell dose 250,000 cells/hippocampus) and the primary outcome (CCDR change at 3-months).

For the benefit of clarity and replication, we have included as a separate document our Animal Ethics Committee-approved main protocol, specifically the version used for most dogs, as well as our final protocol version. Also, further below is a summary of the main protocol changes over the course of the trial.

Major protocol revisions

2013 - Following a surgical SAE and euthanasia in the first patient, Sasha, the trial was halted and recommenced with the following changes: general practice vet to hand over control of post-op care of patient to research team for 2-weeks following treatment; Timing of recommencement of NSAID for management of arthritic pain to be closely reviewed in setting of mild renal impairment because of risk of further renal impairment; Change in staffing to provide single clinician to case-manage patient throughout hospital stay; Introduction of coagulation studies at screening (normal for entry); Introduction of anti-epileptic prophylaxis protocol as well as seizure management plan in the event of post-operative seizures.

2014/5 – Reduction in entry minimum age to 8 years and introduction of Fitbark wearable technology.

2018 – Discontinuation of Canine Sand Maze memory paradigm and introduction of a pilot memory paradigm; trial of EDU labelling of donor cells; discontinuation of NSAID analgesia postoperatively; addition of long term behavioural and clinical assessments.

2019 July – Introduction of Function Cloud wearable device; Introduction of CT skull scan at time of MRI to check safety of fiduciary screw insertion.

2019 August – Major refinement by way of discontinuing fixed skull fiduciary screws and use of University of Sydney Hybrid theatre with intra-operative brain imaging that allows for real-time confirmation of needle tip inside hippocampus.

RODENT METHODS

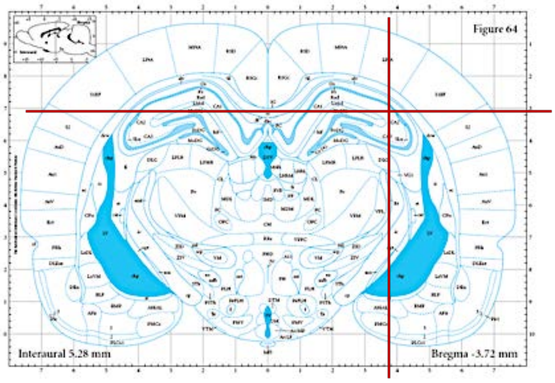

Supplementary Figure SF1. Brain slice from the rat brain atlas (Paxinos & Watson, 1997) showing transplantation coordinates.

RESULTS

Supplementary Table ST1. The DOGS+CELLS Trial individual patients enrolled chronologically 2012-2020.

Patient	Breed	Age at trial entry (years)	Sex	Baseline CCDR	Major Presenting issue	Therapeutic outcome	Final outcome
**Sasha**	Kelpie X Cattle	16	Female spayed	63	Decreased interaction with family members and recognition of family members. Inappropriate urination, getting 'lost', getting 'stuck' and increased vocalisation.	Intraoperative SAE (brain bleed), post-operative seizures and renal failure leading to euthanasia.	Brain donation and likely bleeding disorder on neuropath report.
**Timmy**	Cocker Spaniel	11	Male neutered	57	Sleep/wake cycle disturbance<br>Altered toileting habits<br>Altered interactions with other household dogs	Complete syndromal remission far below diagnostic threshold, durable for approximately 20-months. See **Supplementary Material Video 1** for before and after narrative.	Euthanasia due to complete vision loss and glaucoma pain and brain donation.
**Leo**	Pomeranian X	10	Male neutered	66	Uncharacteristic and unpredictable aggression. Disorientation – staring into space, aimless wandering.<br>Sleep/wake cycle disturbance.	Complete syndromal remission far below diagnostic threshold, durable for approximately 2.5 years. **Supplementary Material Video 2** for before and after narrative.	Euthanasia due to sensory loss and brain donation (September 2020).
**Foxie**	Fox Terrier	16	Female spayed	61	Nocturnal disturbance	**Not treated** because of gastric obstruction in lead up to treatment and owner-elected euthanasia.	Brain donation.
**Gracie**	Siberian Husky	13	Female spayed	60	Disorientation – pacing, staring into space and getting 'stuck'	No therapeutic response and owner elected euthanasia.	No brain donation.
**Maisy**	Border collie	10	Female spayed	64	Disorientation in home and neighbourhood, aimless wandering, staring.	**Not treated** because of large pituitary mass on MRI. Owner-elected euthanasia	Brain donation.
**Grover**	Kelpie	12	Male neutered	66 (11-point decline in 3-months leading up to baseline)	Disorientation – aimless wandering, pacing, circling<br>Sleep/wake cycle disturbance	Therapeutic responder at 3-month primary endpoint but deteriorated by 5-months post treatment. Owner elected euthanasia.	Brain donation.
**Gus**	Labrador X	13	Male neutered	57	Anxiety – manifesting primarily in night-time 'sun downer' episodes. Reduced interaction with his owners.<br>Decreased physical activity.	Therapeutic responder at 3-month primary endpoint (September 2020) to below CCD diagnostic threshold.	Maintains therapeutic response as of November 2020

Patient-specific hippocampal MRI targeting

Supplementary Figure SF2. Each of our canine patients' dorsal hippocampus had customised hippocampal targets calculated because of the diversity of the canine skull, and presence of varying degrees of hippocampal atrophy.

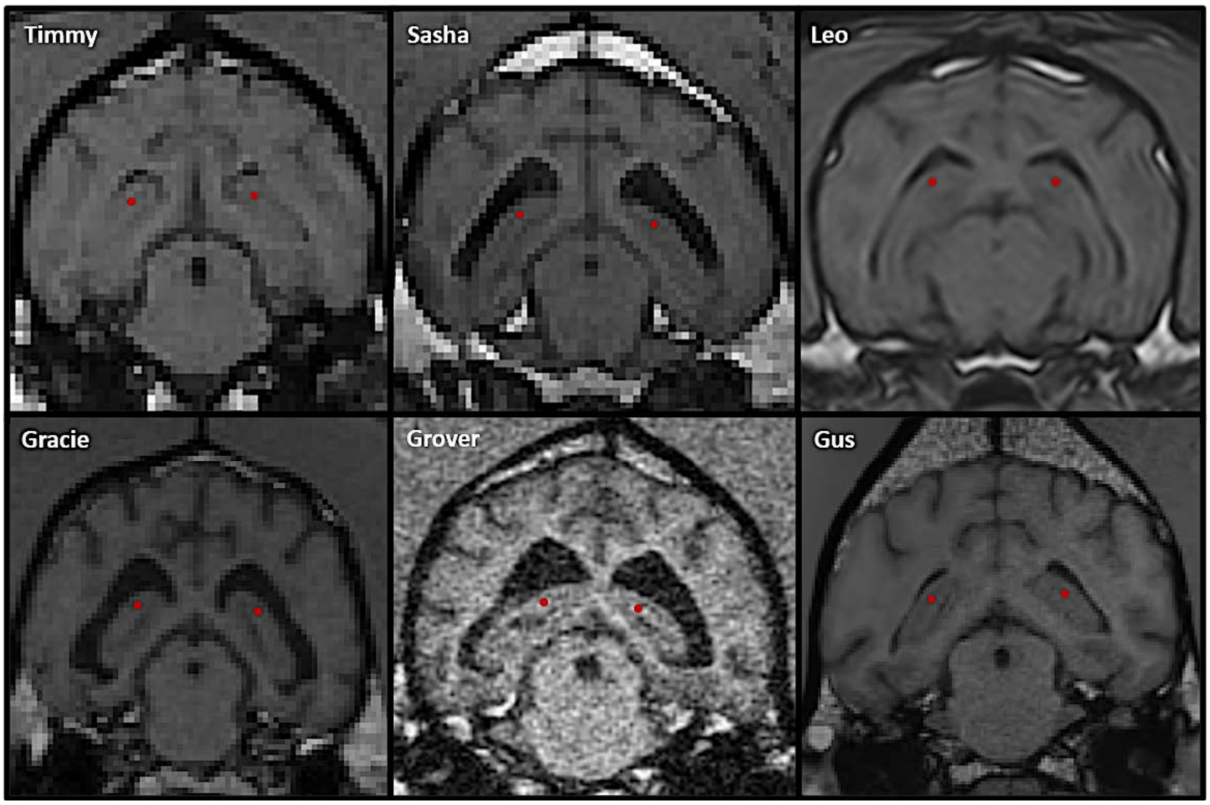

Tauopathy burden quantitative comparisons

S396 total hyperphosphorylated tau pathology was quantified (corrected for corpus callosum CC artefact signal) as per Abey et al (in press) in Grover therapeutic responder and plotted in comparison to N=12 dogs in our Canine Brain Bank.

This shows Grover's tau pathology burden was generally higher than any other dog, potentially explaining his fast rate of clinical decline pre-treatment (worsened by 11 points over 3-months in lead up to treatment), and possibly also explaining why his therapeutic response was limited to 4-5 months duration. Figure show average ± 95%CI.

Supplementary Figure SF3. S396 tau pathology across brain regions in CCD and aged non-CCD dogs in comparison to therapeutic responder, Grover.

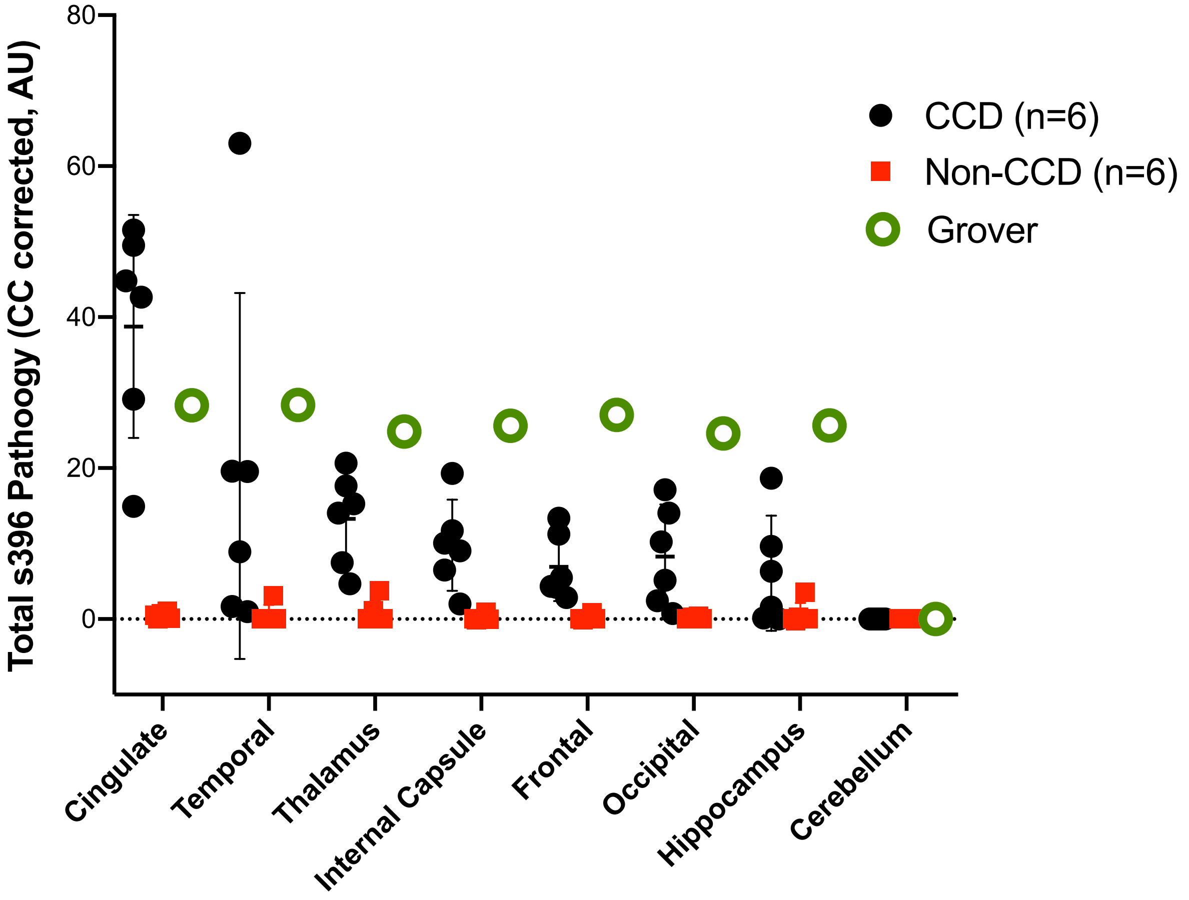

Quantitative co-expression of early neuronal and synaptic markers

Supplementary Figure SF4. Chart showing early neuronal *βIII tubulin* in dorsal hippocampus (region of cell delivery) in therapeutic responders is specifically and markedly increased, 4-5 SDs higher than in non-treated aged animals (n=10).

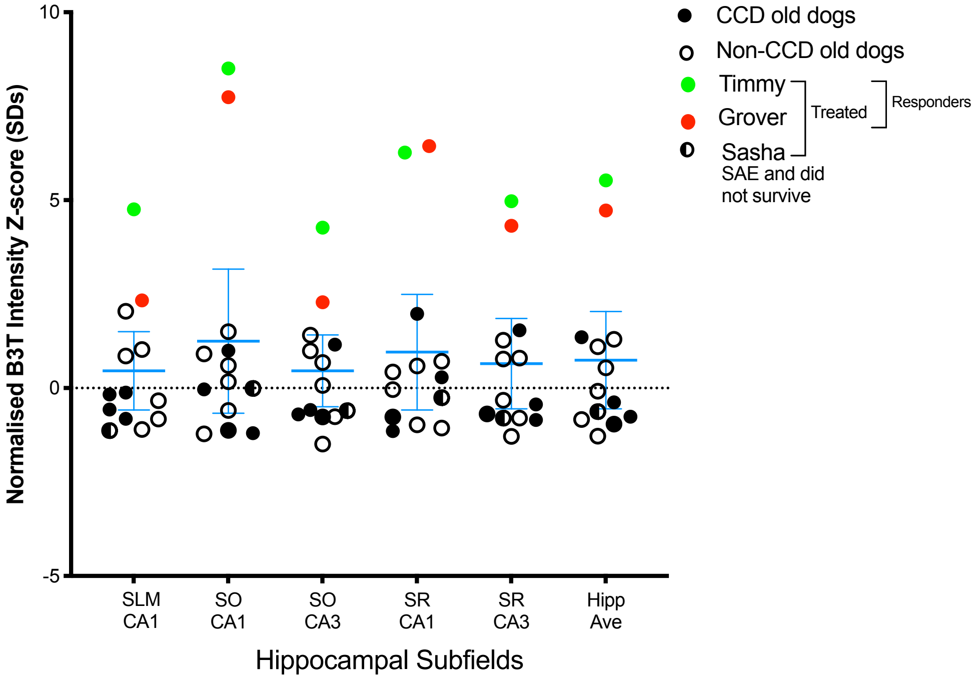

Supplementary Figure SF5. Chart showing early neuronal *βIII tubulin* in ventral hippocampus (remote from region of cell delivery) in therapeutic responders is unchanged compared non-treated aged animals (n=10).

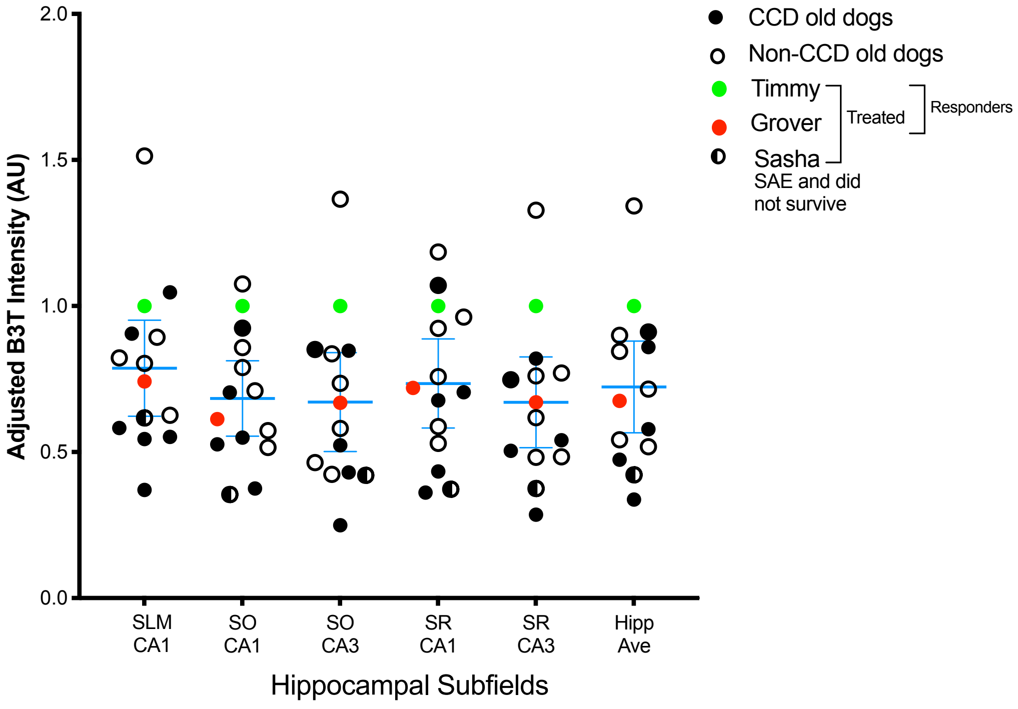

Supplementary Figure SF6. A) Rabbit IgG histological image (in red channel) that served as antibody control for synaptophysin from a treated dog patient (Timmy) in hippocampal area CA3 stratum oriens, co-stained with *βIII tubulin* (green). B) Comparison with staining for *synaptophysin* (red) and *βIII tubulin* (green) in adjacent section from same region. Nuclear *dapi* (blue) in all images which are as taken from the microscope. These confirm that synaptophysin antibody and histology protocol produced specific reactivity to this presynaptic protein.

A

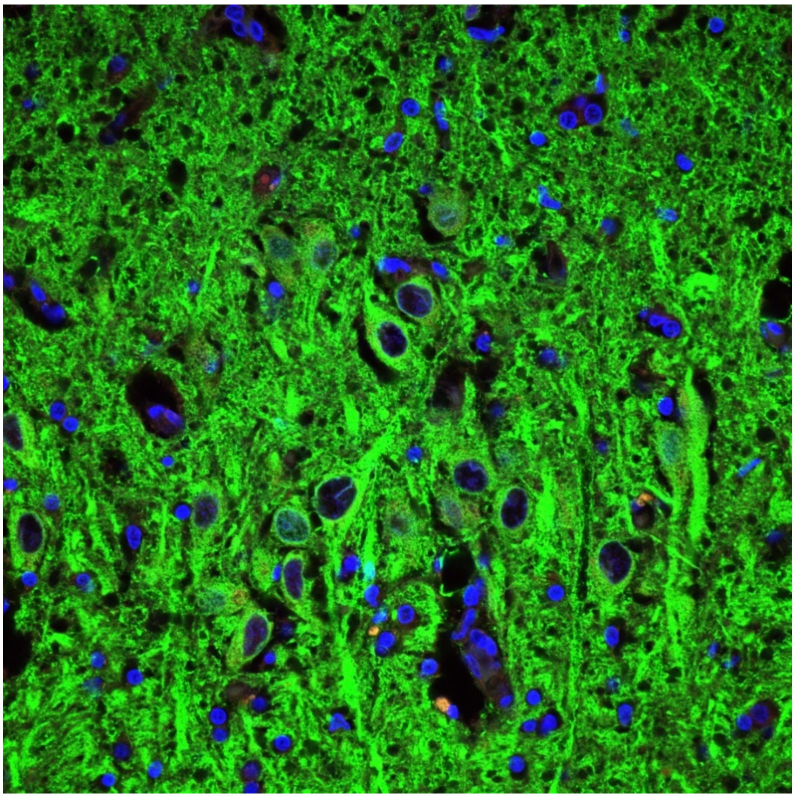

B

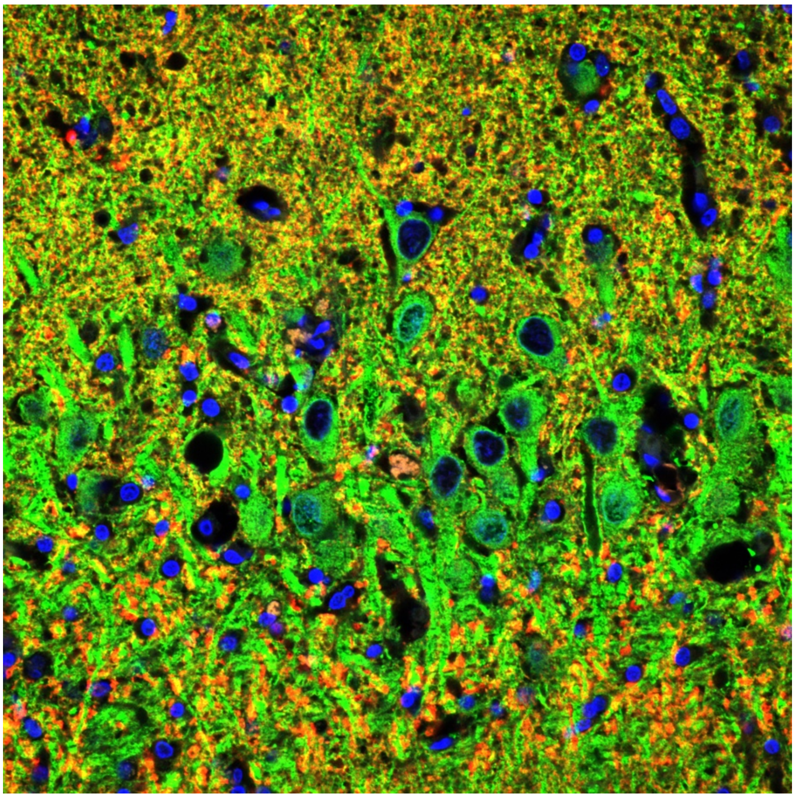

Supplementary Figure SF7. Representative hippocampal dentate gyrus (stratum lacunosum-moleculare) histological images from treated and untreated canine patients with staining for early neuronal marker *βIII tubulin* (green), synaptic marker *synaptophysin* (red), nuclear *dapi* (blue) and co-expression in yellow of immature neuronal presynaptic punctae. Scale bar 50microns.

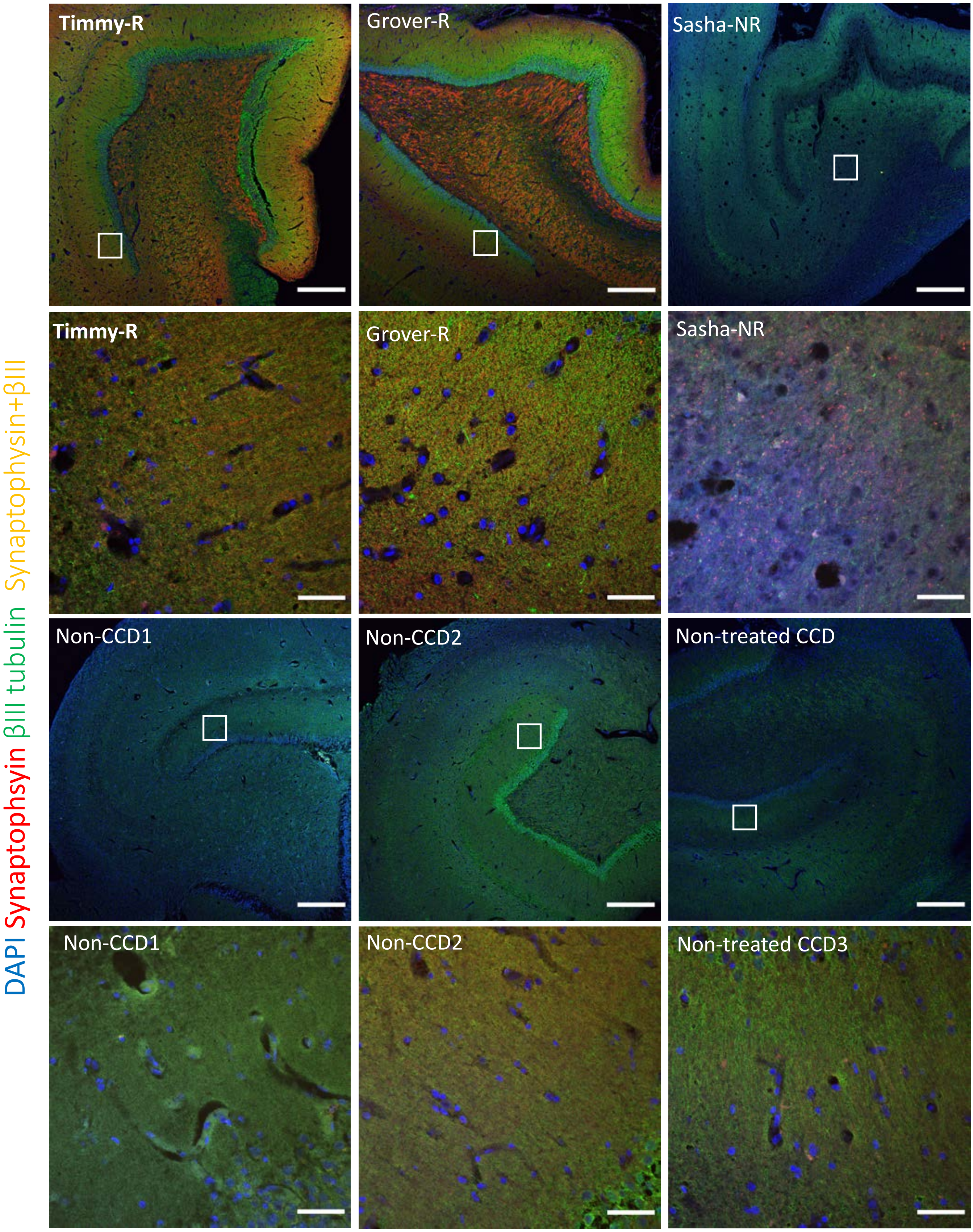

Supplementary Figure SF8. Representative hippocampal CA1 (stratum oriens) histological images from treated and untreated canine patients with staining for early neuronal marker *βIII tubulin* (green), synaptic marker *synaptophysin* (red), nuclear *dapi* (blue) and co-expression in yellow of immature neuronal presynaptic punctae. Scale bar 50microns.

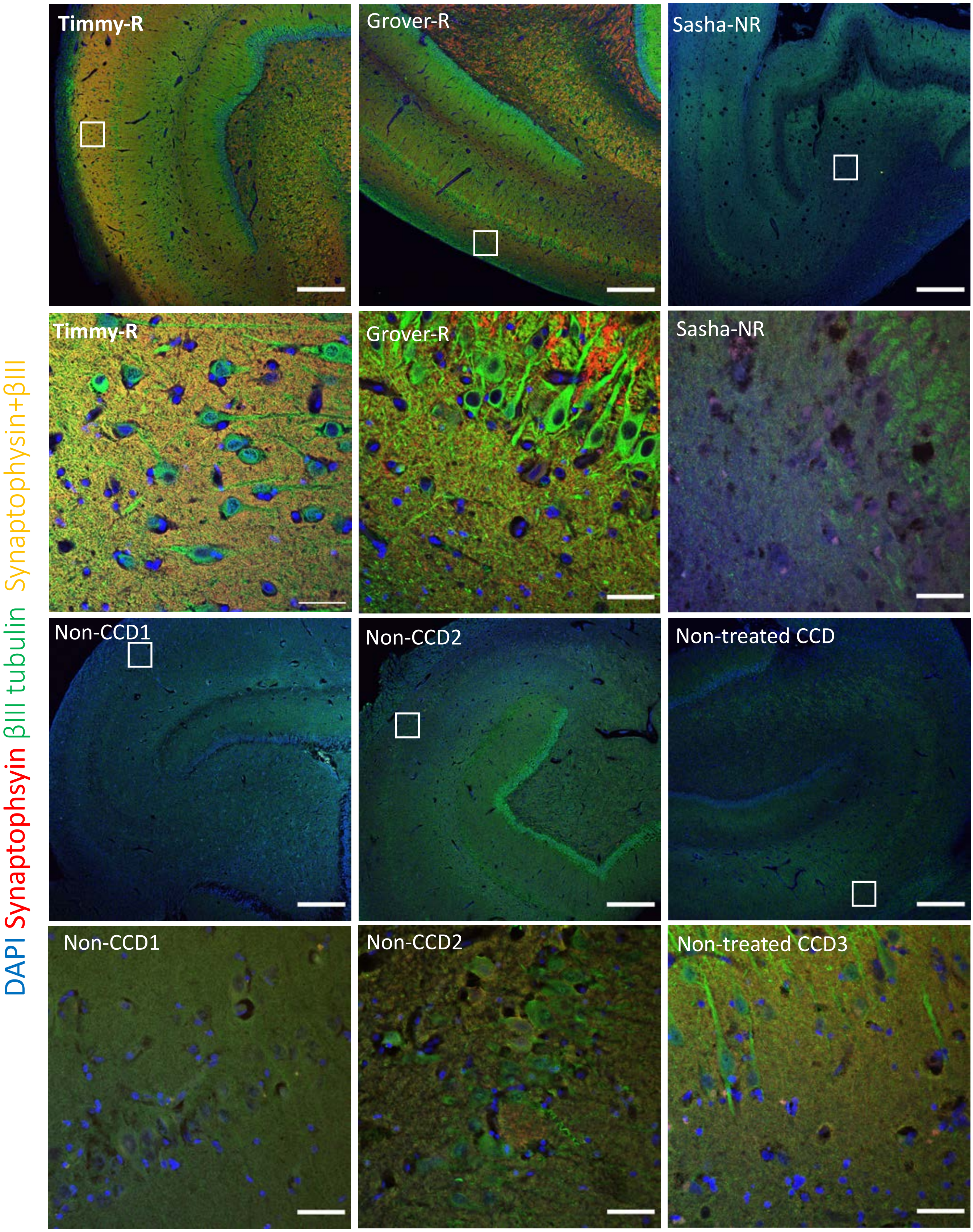

Supplementary Figure SF9. Representative hippocampal CA3 (stratum radiatum) histological images from treated and untreated canine patients with staining for early neuronal marker *βIII tubulin* (green), synaptic marker *synaptophysin* (red), nuclear *dapi* (blue) and co-expression in yellow of immature neuronal presynaptic punctae. Scale bar 50microns.

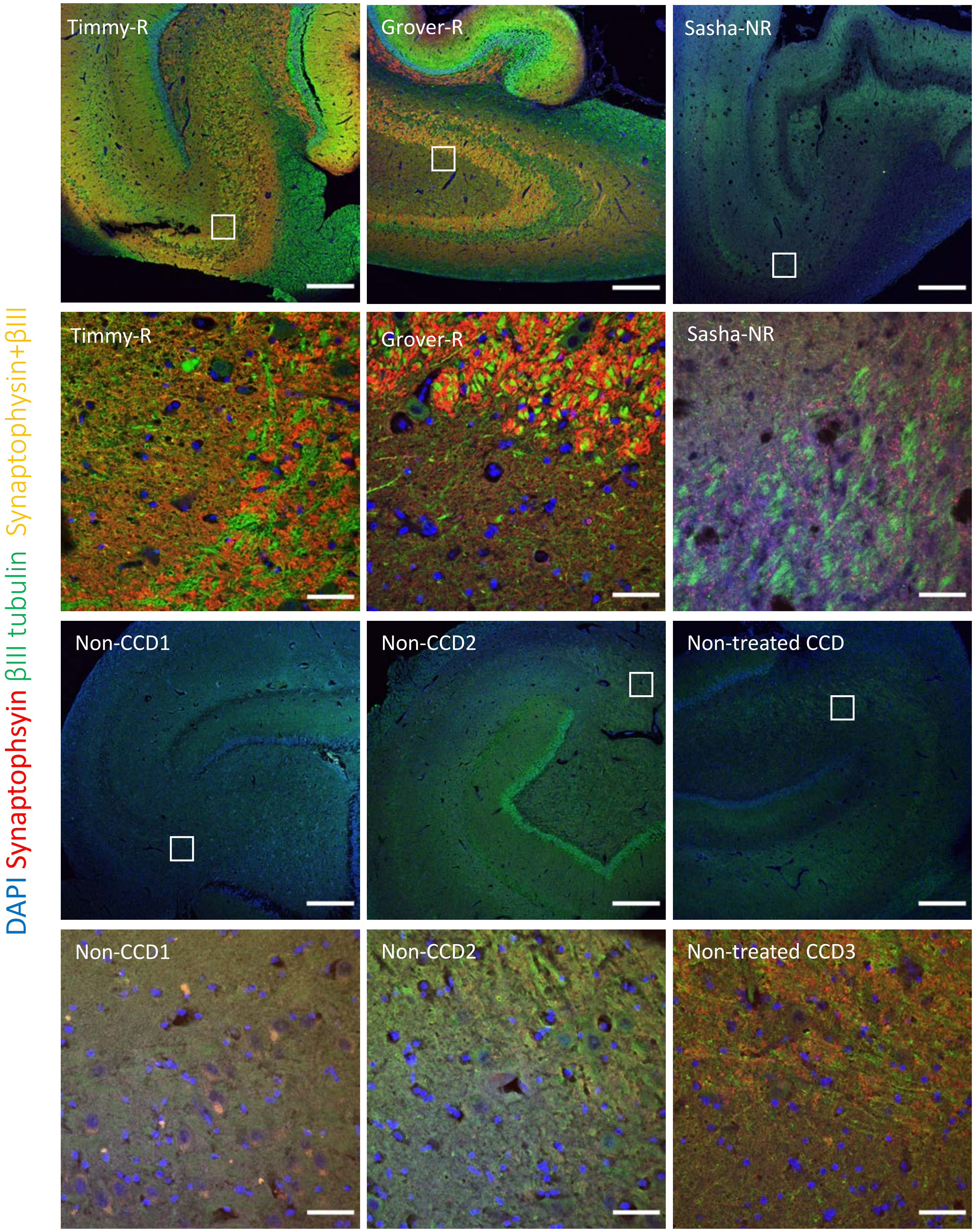

Canine Sand Maze (CSM)

Leo's learning trial results show significant within-subject improvement between pre- and post- treatment. At pre-treatment Leo failed the probe trial (did not enter the reward zone up within 90sec when there was no food reward present, following a 90-minute delay after the end of learning), and travelled directly to the reward zone within 5sec on probe trial at 3-month after treatment.

Supplementary Figure SF10. CSM results for Leo learning trials.

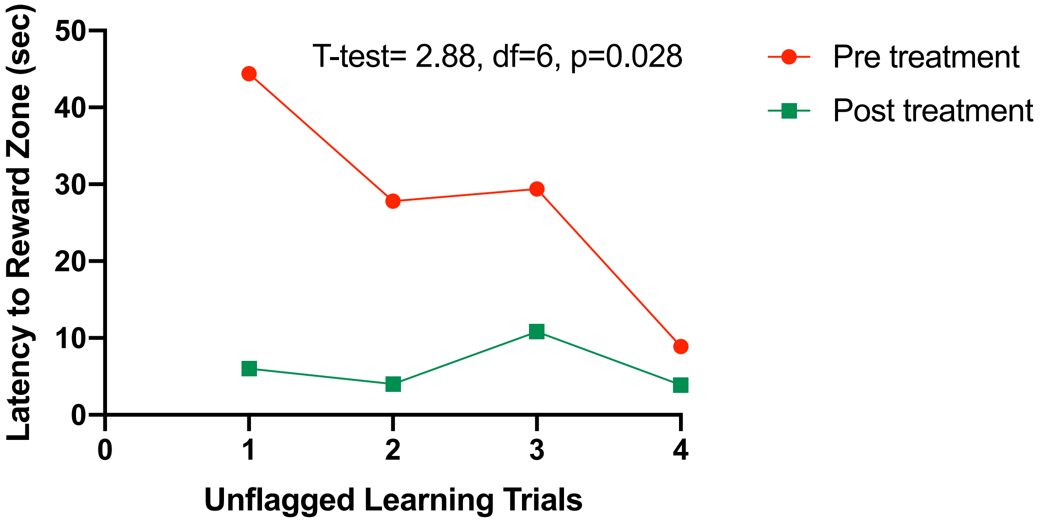

Timmy's CSM learning trials did not show significant within-animal improvement between pre- and post-treatment, however following treatment he immediately orientated towards the correct reward quadrant on trials 2,3 and 4. In the final learning trial he also immediately approached and explored the reward zone (**Figure 1D** main manuscript). There were no differences in probe trial performance before and after treatment (failed both attempts).

Supplementary Figure SF11. CSM results for Timmy learning trials.

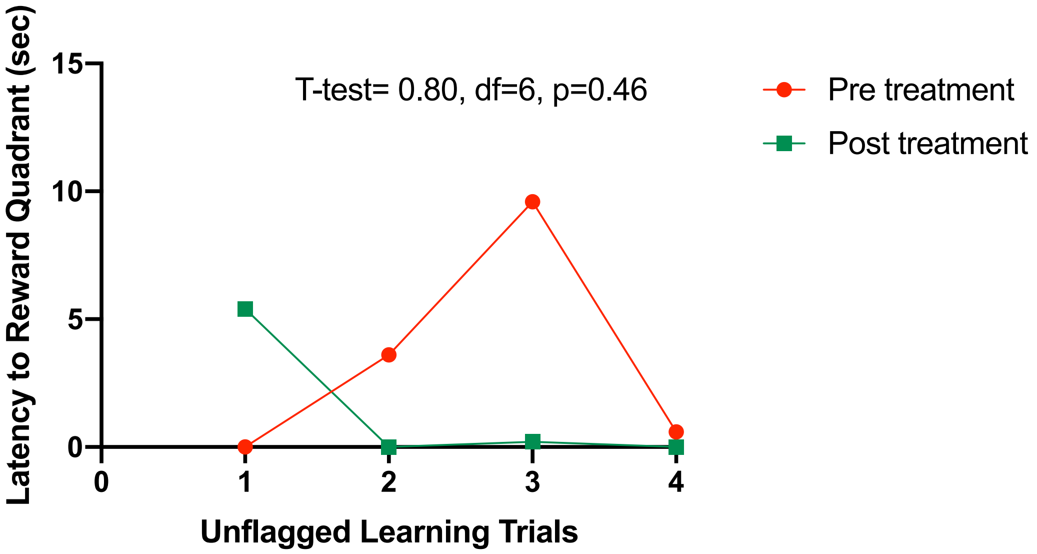

Function Cloud Analysis: Gus homebound movement

Daily spatial heat maps were generated for Gus (next page) for a week prior to transplant and for the last week of follow up (week 12 post-treatment), illustrative of total movement in the home. Images were calibrated, colour-thresholded and area fraction calculated in Image J.

The graph clearly shows that Gus' in-home spatial wandering area significantly reduced by 50% following treatment (T-test = 5.047, df=10, p=0.00005). Figure shows average ±95%CI.

Supplementary Figure SF12. Function Cloud results for Gus before and after treatment.

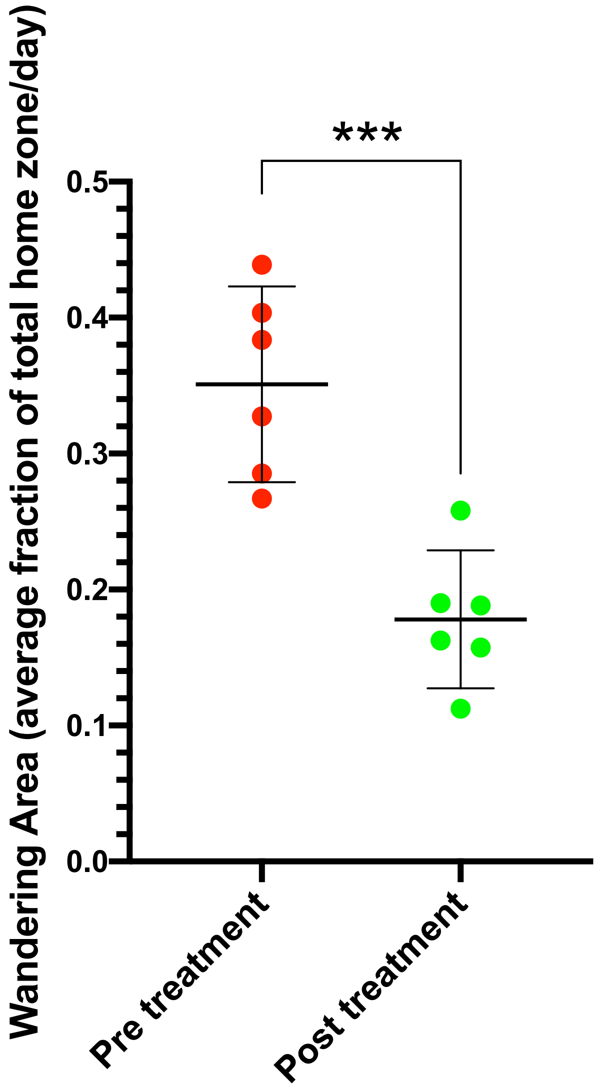

Supplementary Figure SF13. Function Cloud spatial maps for Gus over 5 days pre and post transplant.

Pre-transplant

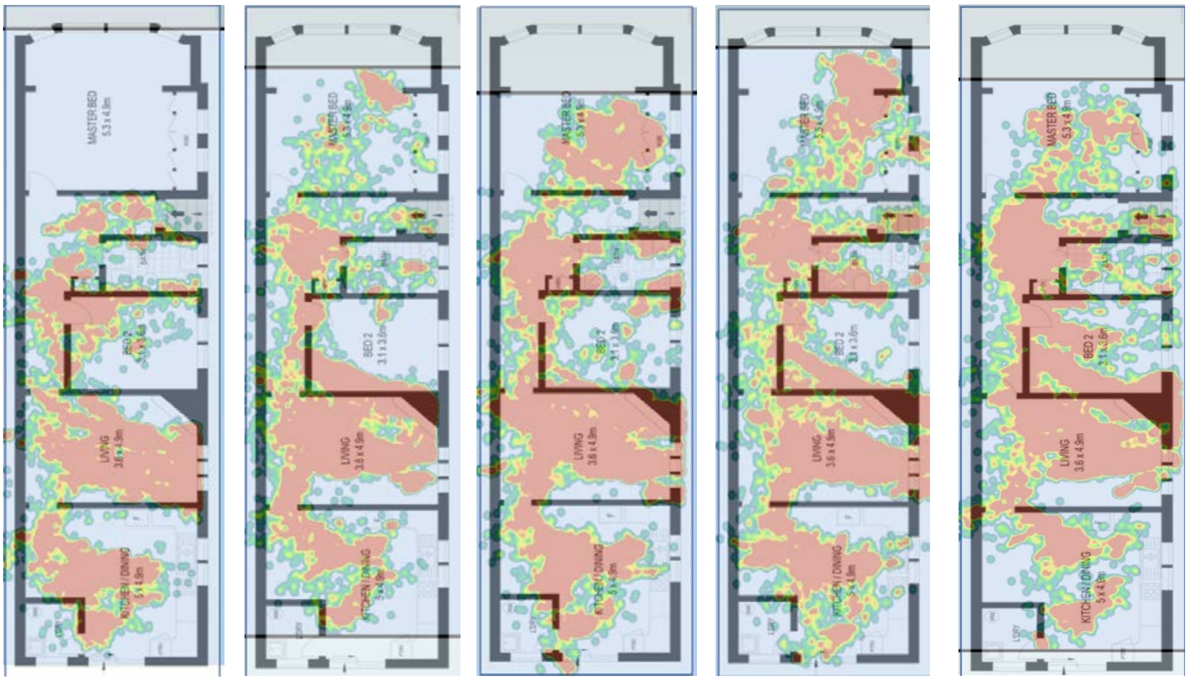

Post-transplant

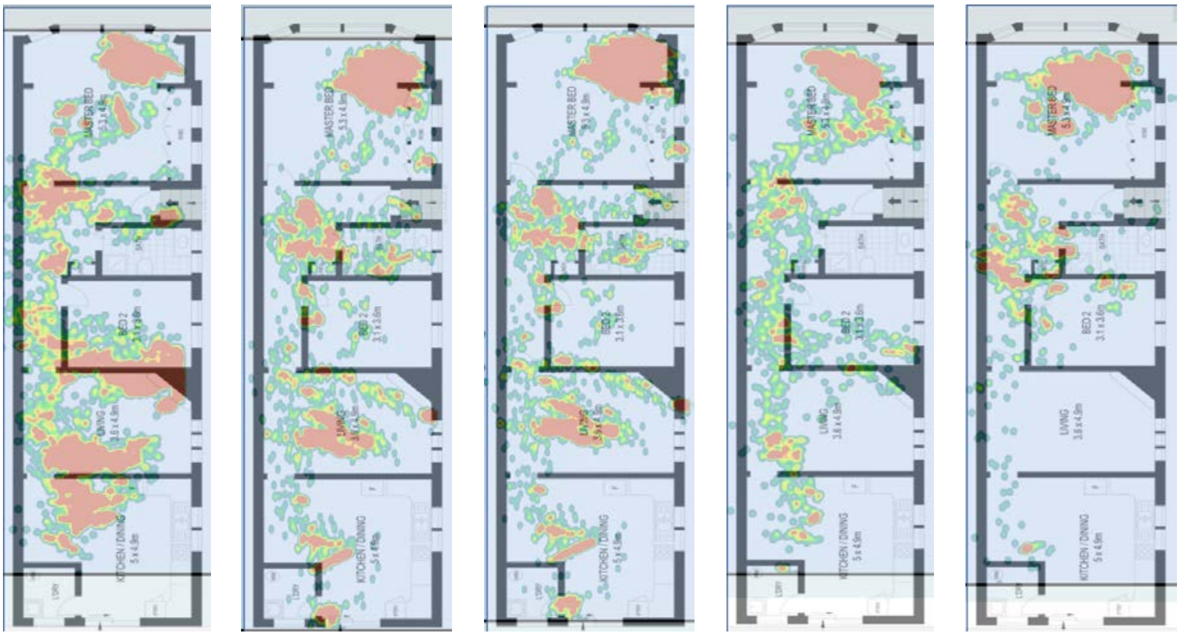

Supplementary Figure SF14. A) Leo SKN cell line following 21 days *in vitro* neuronal differentiation shows typical neuronal morphology and mature marker expression (*Neurofilament,* green*)*. B) Negative control canine fibroblast line shows no expression of *neurofilament* (green channel). C) Positive control cells (NSC34) shows robust expression of *neurofilament* (green) but without as extensive neuritic morphology. All images captured under identical microscope settings.

A

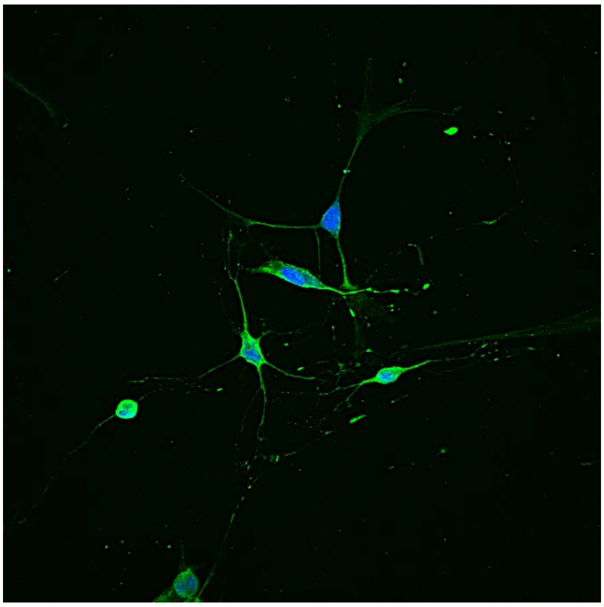

B

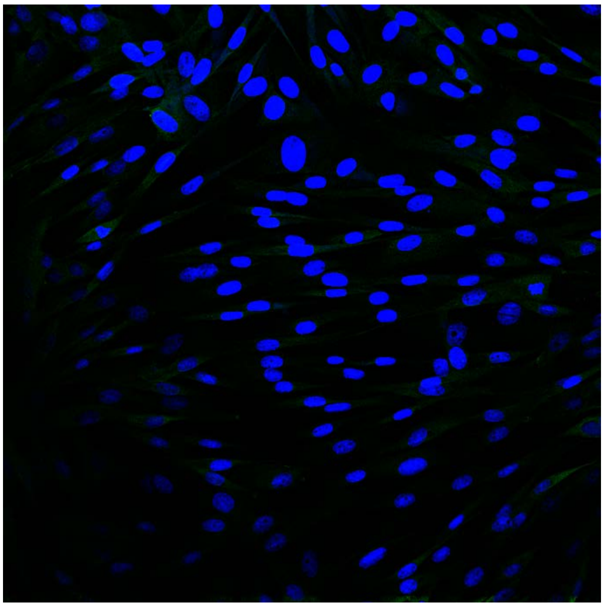

C

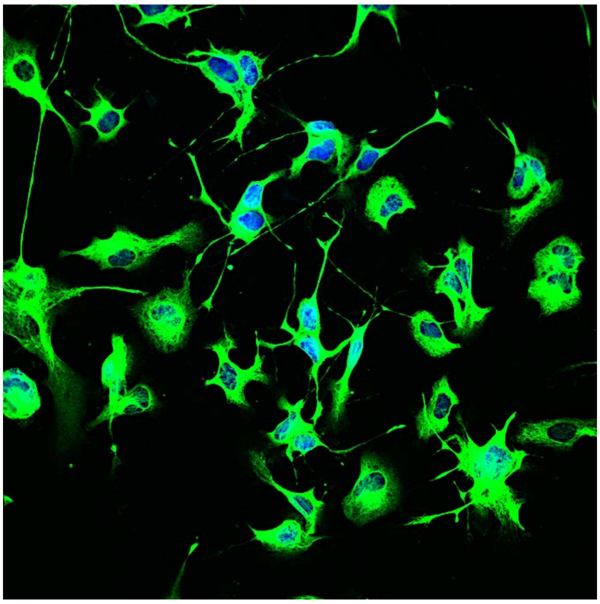

RODENT STUDY

Donor cell canine-specific lamin expression

We used a Santa Cruz anti-lamin antibody (Lamin A/C 636 sc-7292) that is documented to detect porcine and human nuclear lamin. We first validated positive expression in canine SKNs *in vitro* (below) and no expression in the untreated rat brain (next page).

Supplementary Figure SF15. Immunofluorescence image showing lamin expression: Canine SKNs *in vitro* prior to transplantation double stained with lamin (red) and DAPI (blue). Scale bars = 50 μm.

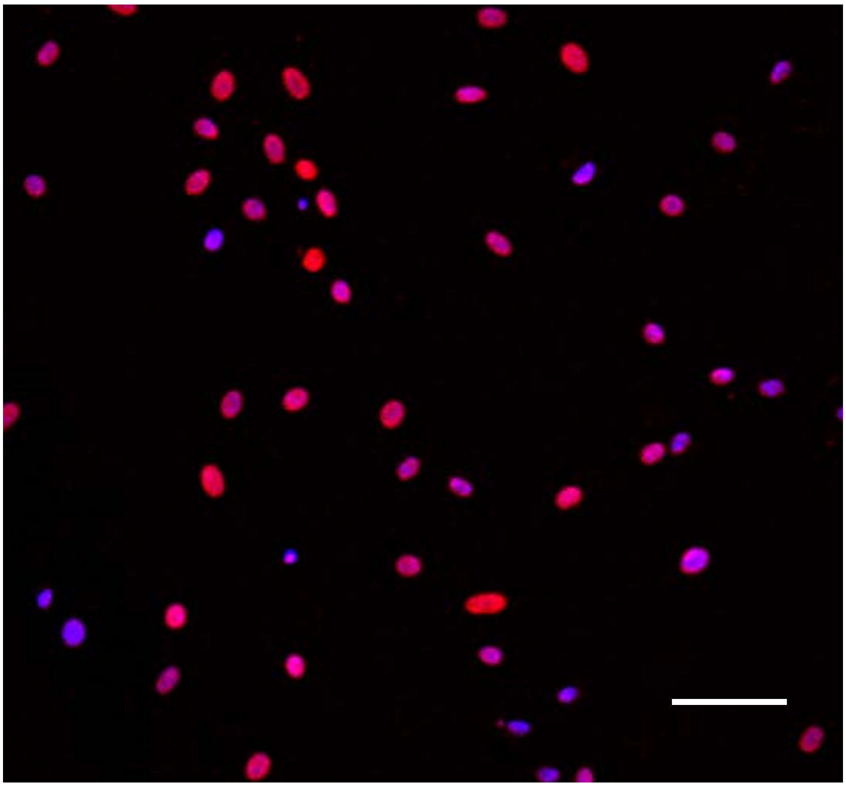

Sham transplant Lamin-DAB brightfield histology negative control

Supplementary Figure SF16. Hippocampal photomicrographs of SHAM transplanted rodent hippocampus. Non-cell transplanted SHAM animals exhibited no signal following lamin canine-specific antibody staining (brown). Counterstained for cresyl violet (blue). A) CA1 pyramidal layer, B) dentate gyrus and C) hippocampus.

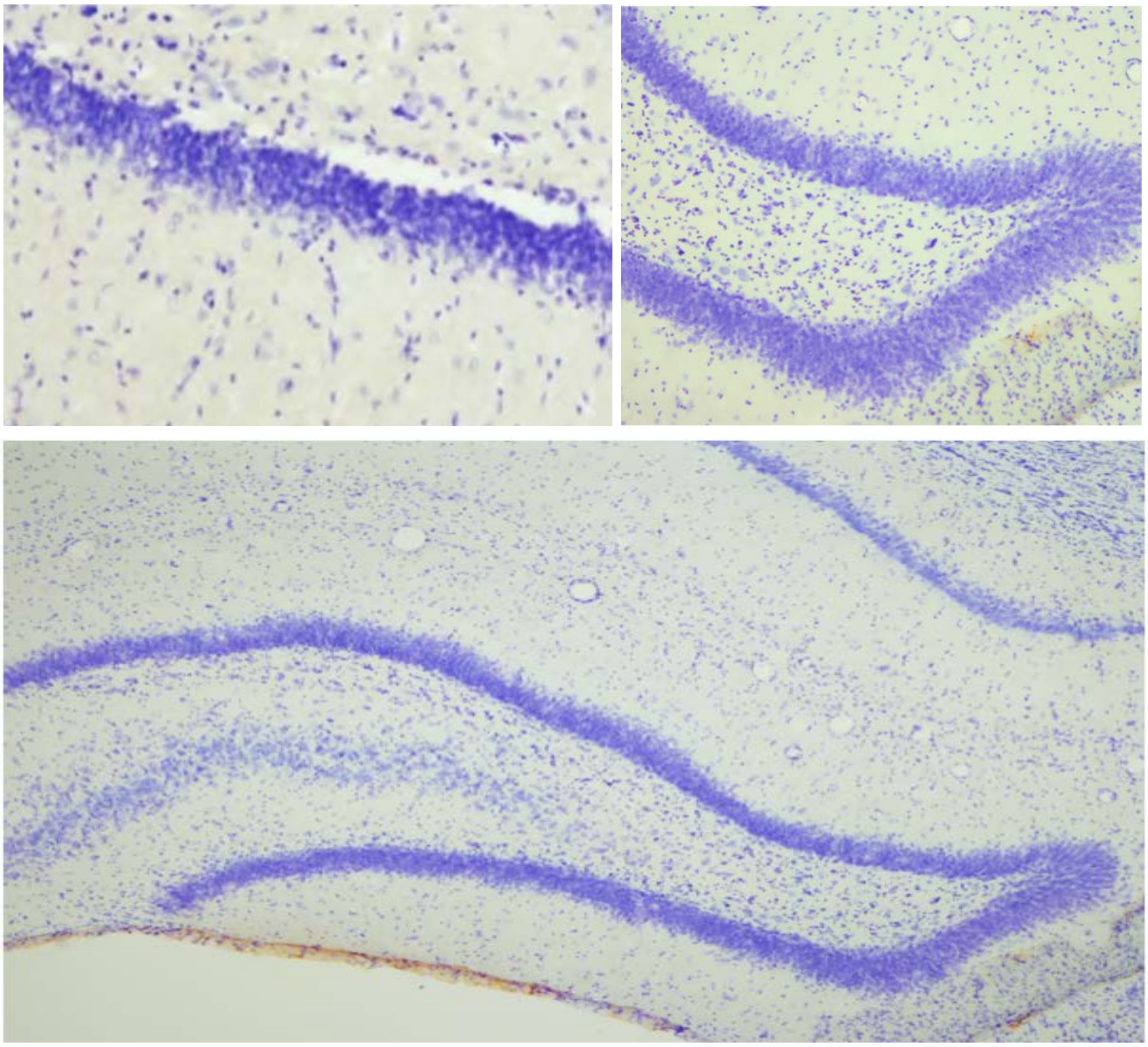

Sham transplant immunofluorescence histology negative control

Supplementary Figure SF17. CA1 rodent hippocampus imaged under same conditions as used for GFP-labelled cell transplants (extension study) show no artefactual green signal.

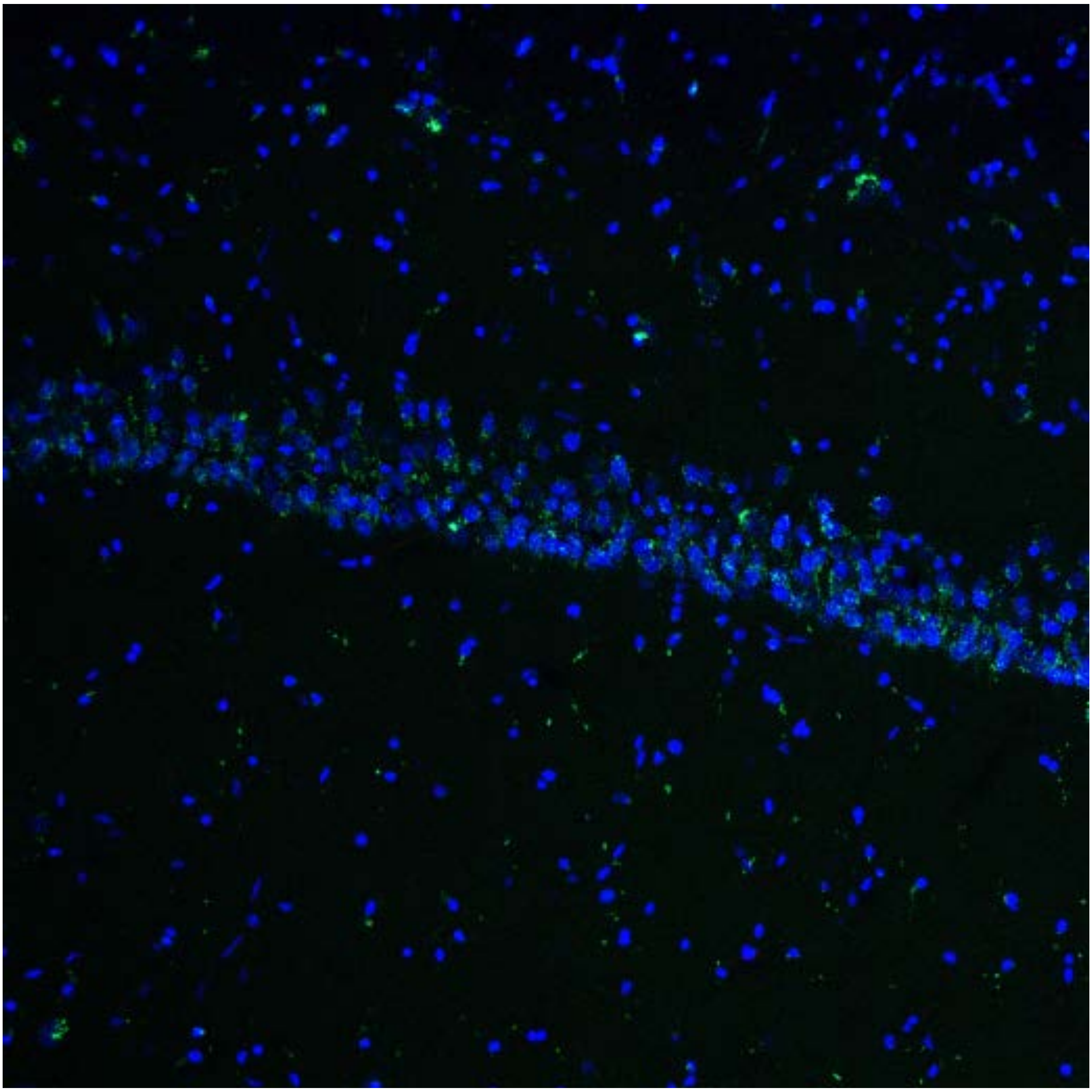

SKN Lamin-DAB histology (main experiment, N=16)

Supplementary Figure SF18. Panel below shows exemplar images of Lamin-DAB+ (brown) donor SKN cells distributed throughout different subfields of hippocampus counterstained with cresyl violet: dentate gyrus, CA2, and CA3. Scale bar 50microns.

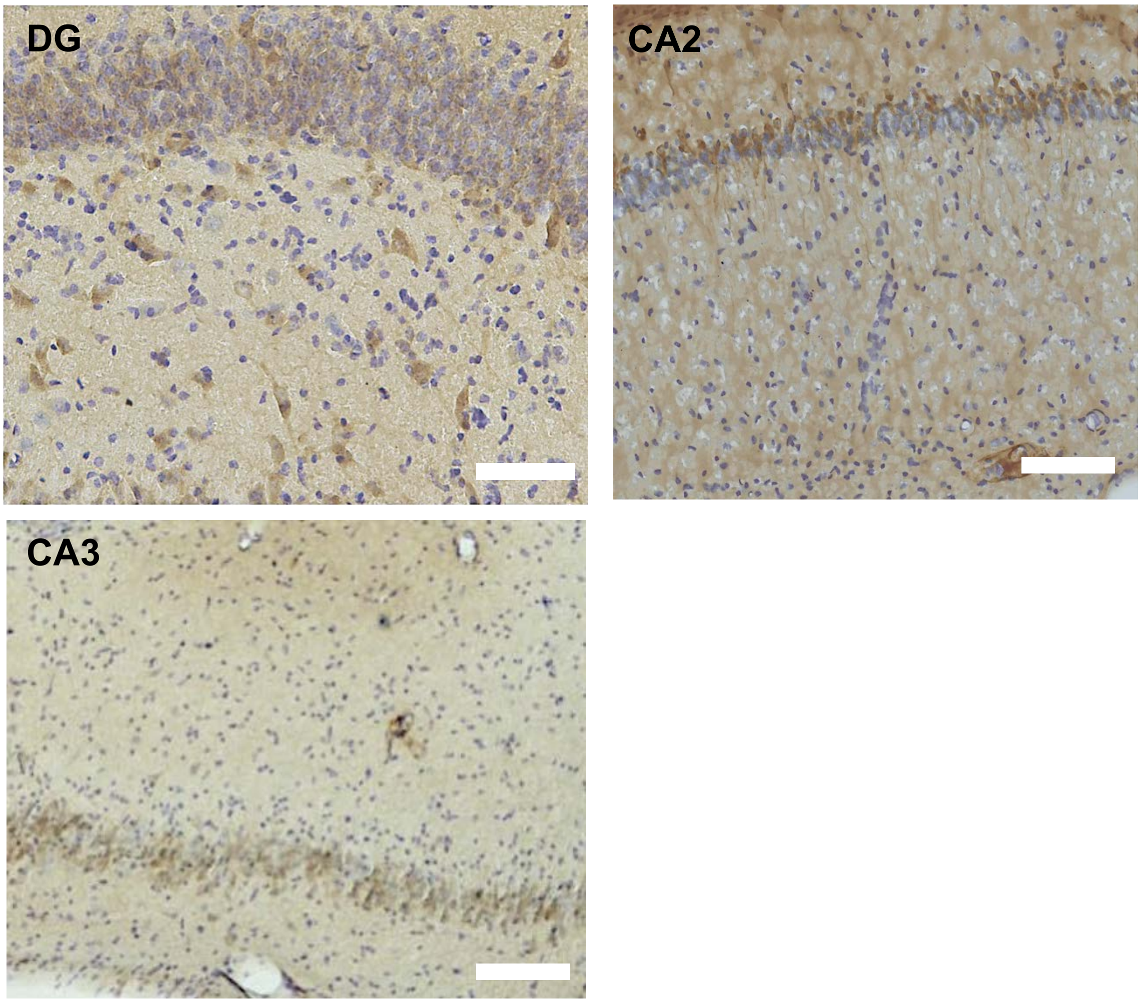

SKN lamin confocal immunofluorescent images (main experiment)

Supplementary Figure SF19. Panel below shows exemplar images of extensive Lamin-positive donor SKN cell (green) engraftment in CA1 (left column) and dentate gyrus (right). Images in bottom row show co-expression with ßIII-tubulin (red). Donor cell bodies appropriately aligned along pyramidal or granular layer with anatomically-correct dendrites. Some artefactual green signal in blood vessels also visible.

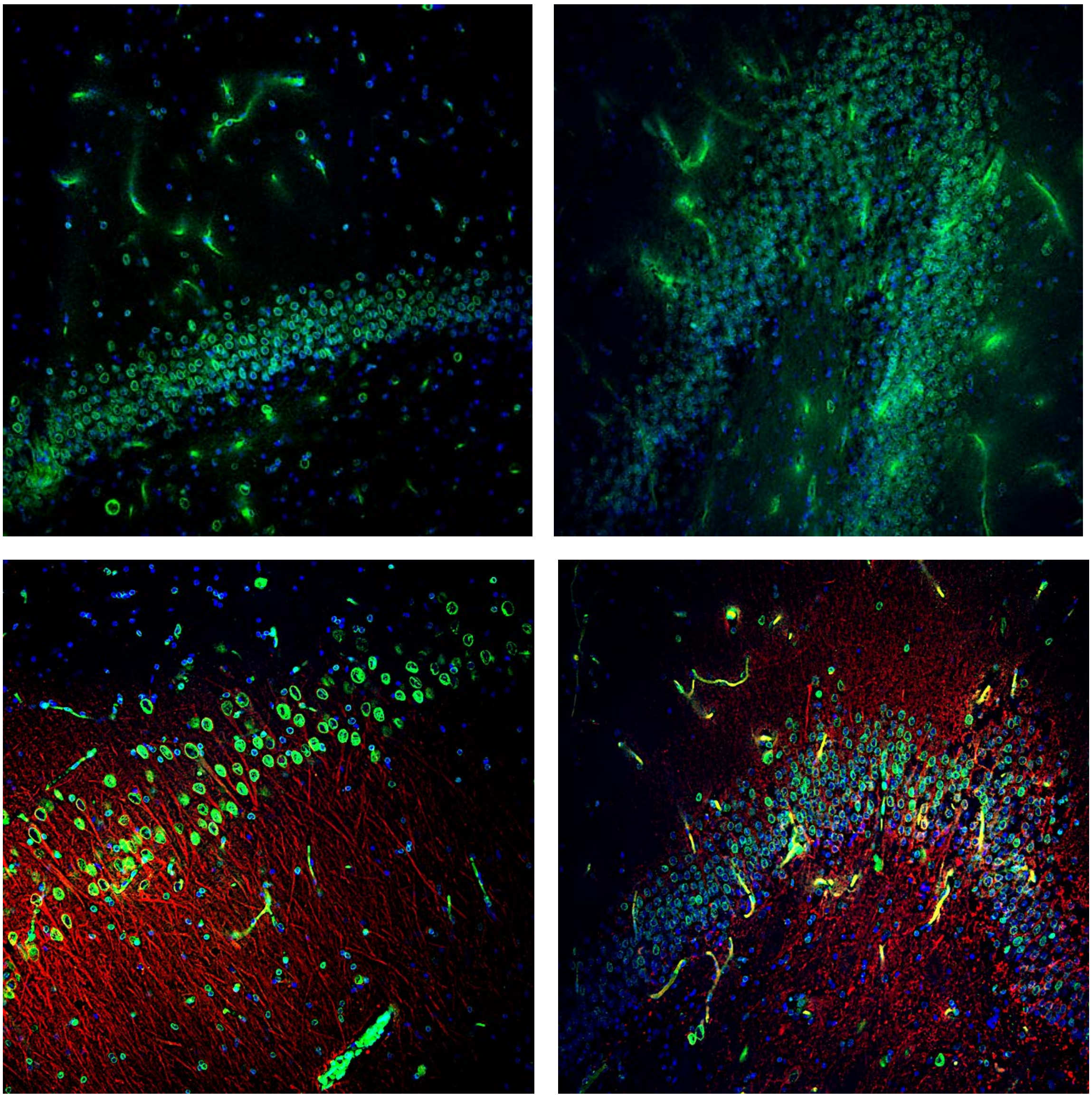

SKN-GFP histology (extension study N=2)

Supplementary Figure SF20. Panel below shows exemplar immunofluorescent images in CA1 of both rats, showing co-expression of donor cell GFP and two different mature neuronal markers, NeuN and Neurofilament (NF). All images, as per **Figure 4D,E** of main manuscript show extensive engraftment in CA1 including cell bodies aligned along pyramidal layer and dendrites extending down into stratum radiatum.

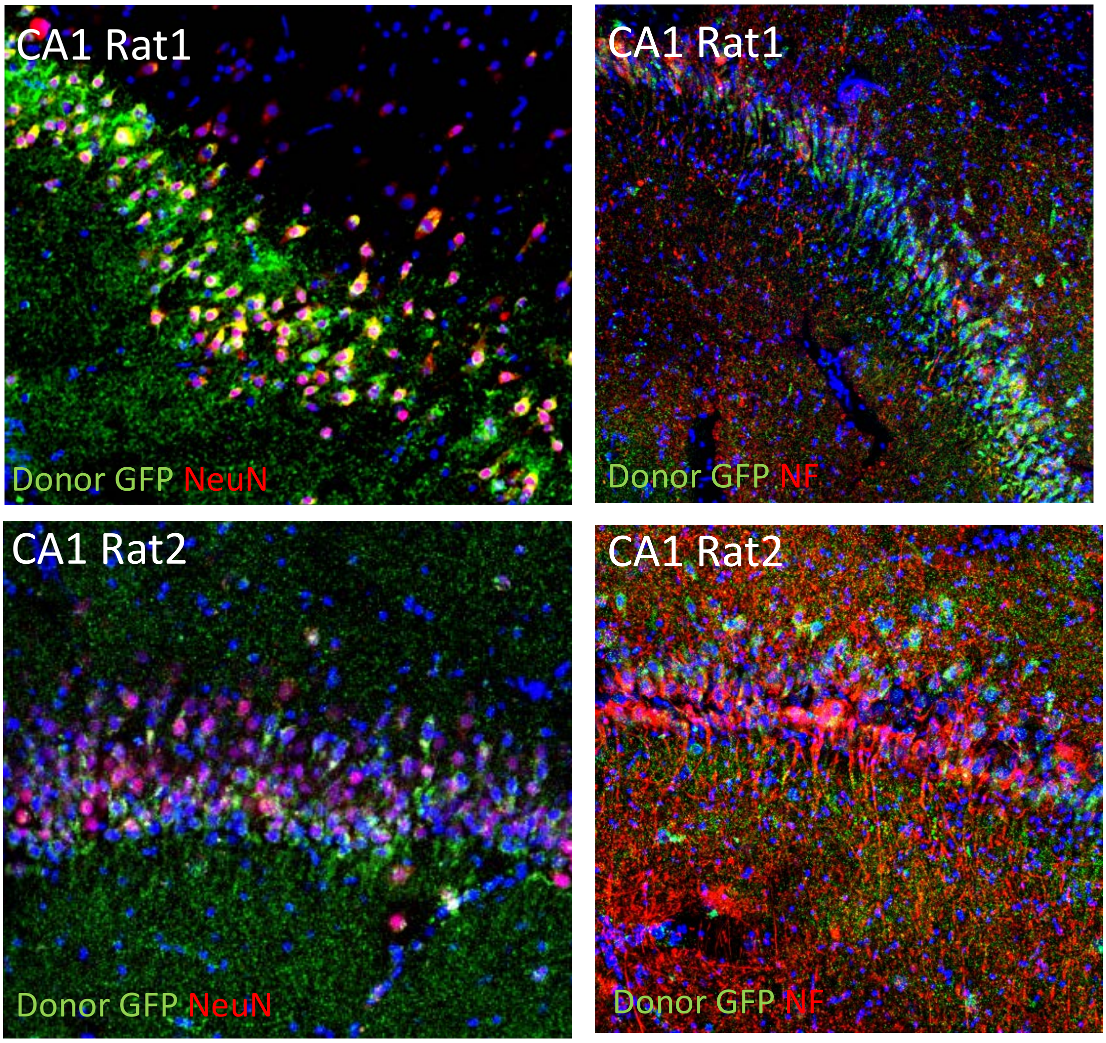

High magnification of secondary dendrite on cSKN donor cell *in situ* showing intense decoration with synaptic spines

Supplementary Figure SF21. Figure shows zoom into zone marked in main manuscript **Figure 4H** (biocytin-filled donor cell that underwent *in situ* electrophysiology).

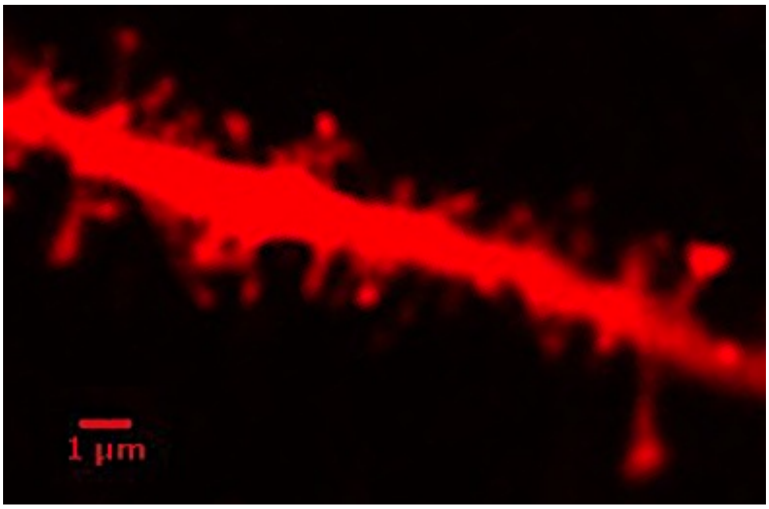

Supplementary Figure SF22. Orthogonal view of a CA1 Pzs-Green1 donor SKN cell (green) co-labelled with biocytin (red). XYZ views confirm green donor signal is wholly within biocytin-filled cell. Same cell examined with live slice electrophysiology and z-stack image in main text **Figure 4H-L**.

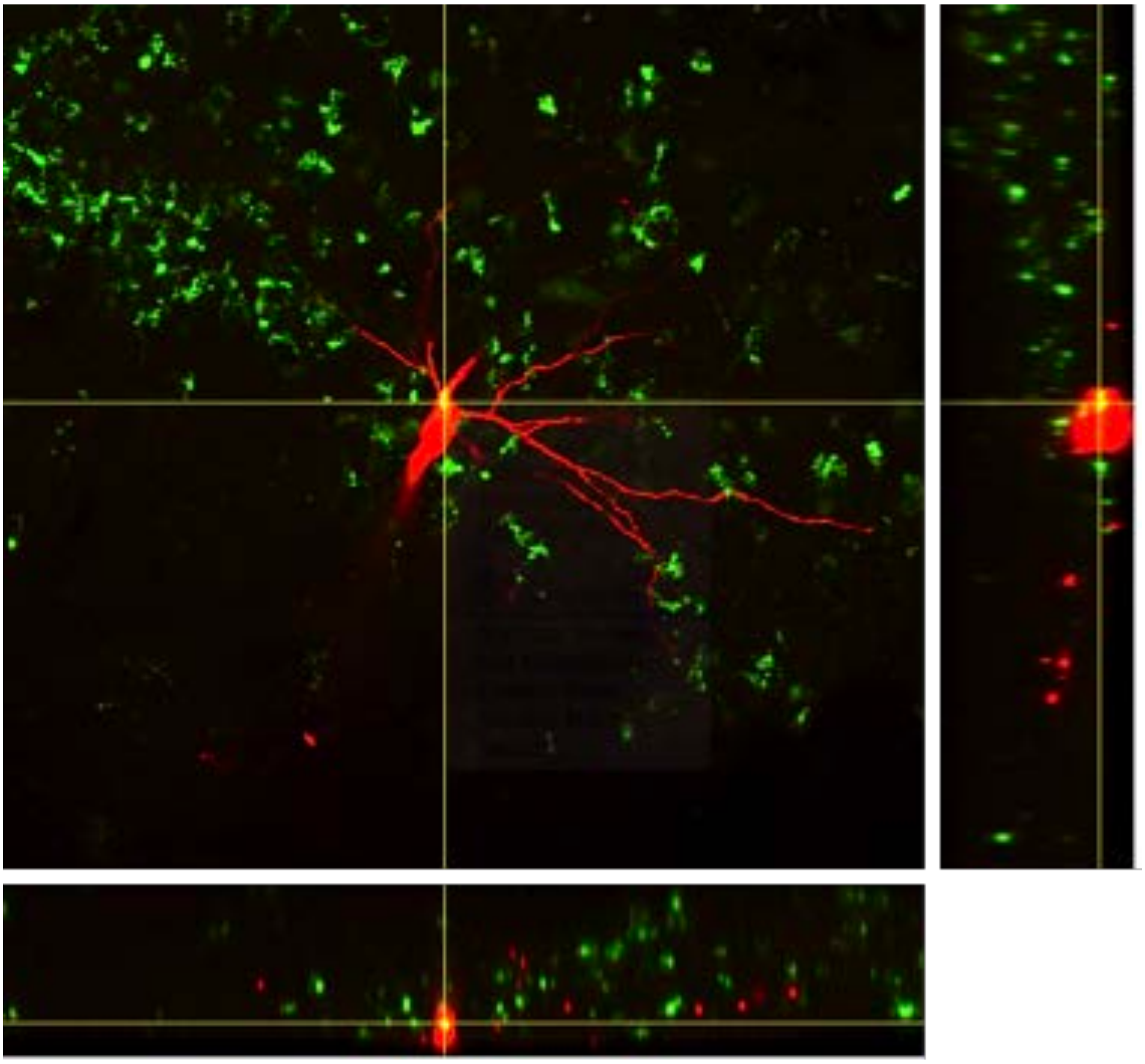

Supplementary Figure SF23. Large format view of **Figure 4H**: Pzs-Green1 donor SKN cell (green) co-labelled with biocytin (red) engrafted in CA1 pyramidal layer. Z-stack image (maximum projection). Despeckled in FIJI. Scalebar 50micron. Flythrough movie available as **Supplementary Movie SM3**.

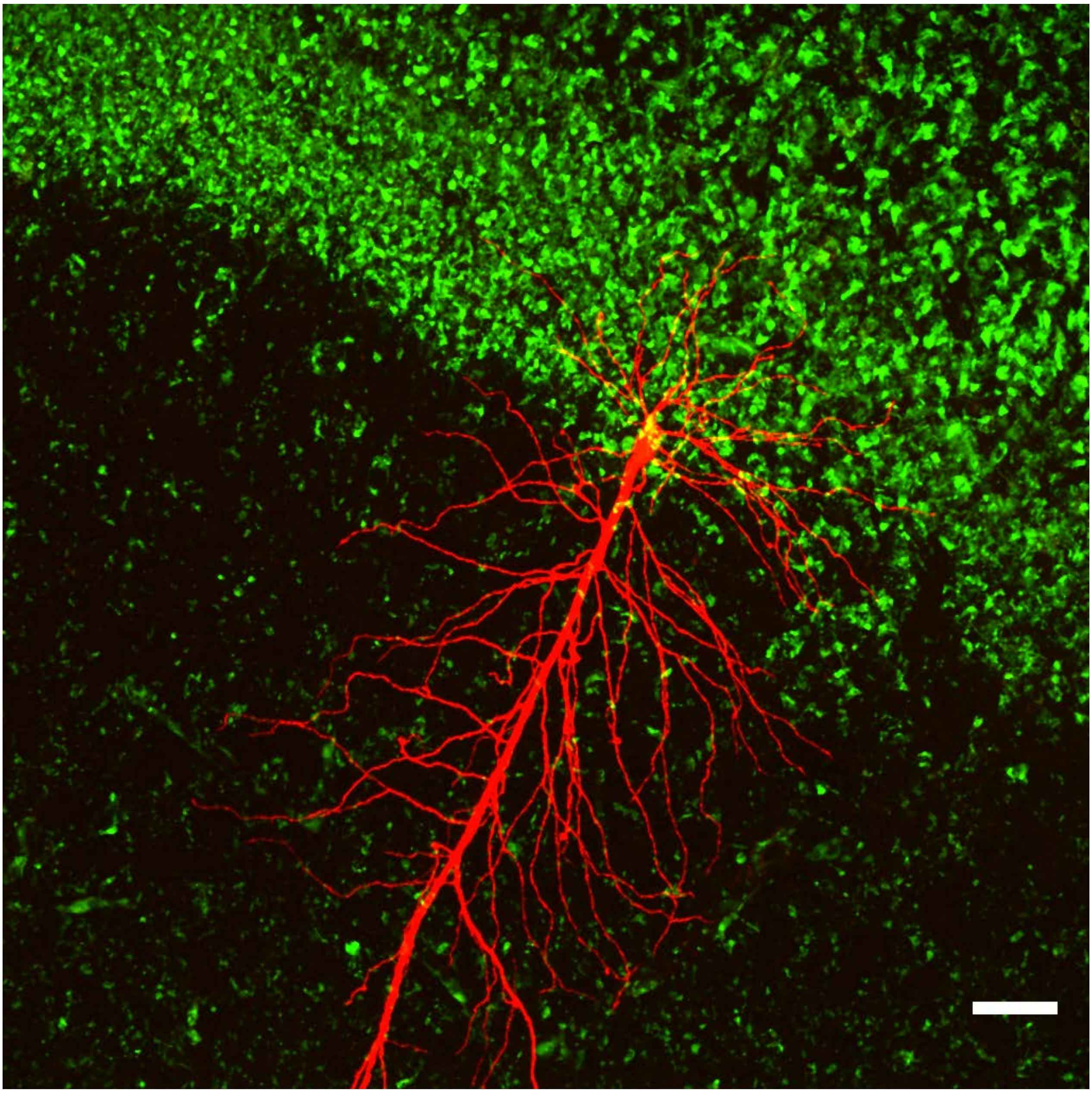

Place recognition memory individual rodent changes pre- and post- transplant.

Supplementary Figure SF24. Pre-transplant, all groups demonstrate PRM impairments. A) In the Sham treated group there was only one spontaneous recovery that was near threshold at baseline. B) 4/6 animals below threshold in the SKN cell treatment group were rescued.

A

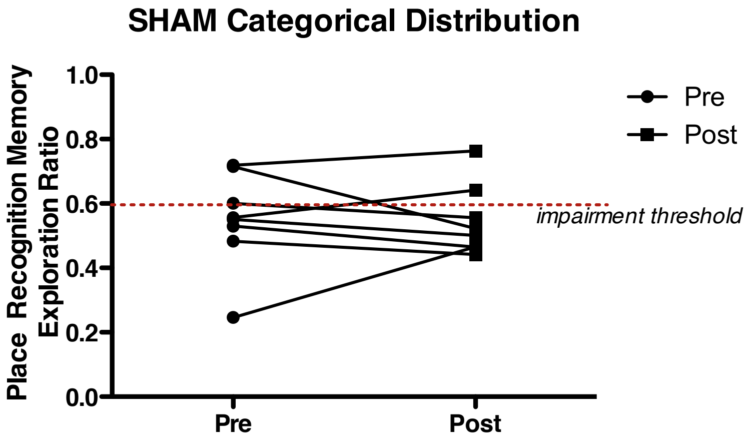

B

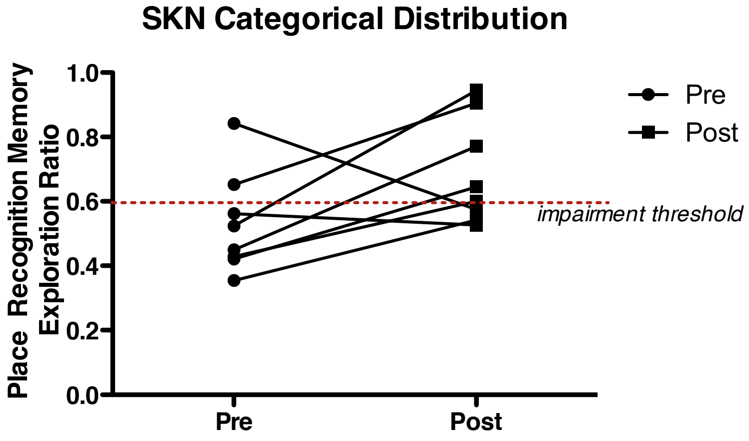

Supplement: Supplementary file 1 — Additional file 1. Supplementary Materials. [file 13287_2022_2933_MOESM1_ESM.pdf]
