# Supplementary material for: Autologous skin-derived neural precursor cell therapy reverses canine Alzheimer dementia-like syndrome in a proof of concept veterinary trial
Source: Stem Cell Res Ther. 2022 Jun 17;13:261. doi: 10.1186/s13287-022-02933-w (PMC9205057; doi:10.1186/s13287-022-02933-w)
Supplement: Supplementary file 2 — Additional file 2. Supplementary Protocols. [file 13287_2022_2933_MOESM2_ESM.pdf]

Reversal of Canine Cognitive Dysfunction by Hippocampal Transplantation of Dog-specific Skin-derived Neural Precursors

Protocol: Version 8

28 August, 2013

Short title: The Dogs + Cells Trial

Index

**Index**	**1**
Team	2
Independent Veterinary Safety Review Committee	2
Significance	3
Aims	4
Background	4
**Study 1: Dogs + Cells Safety Study**	**7**
Aim	7
Overview of Procedures & Timeline	7
Recruitment	8
Selection criteria	8
Veterinary “fitness for trial’ checklist	9
MRI	11
Intrahippocampal stereotaxic co-ordinates	11
Skin Biopsy	12
SKN culture	12
SKN Cell Quality Control	15
Baseline physical activity	17
Period of increased physical activity	17
Definitive baseline CCDR	17
Baseline Canine Sand Maze	19
Surgery	21
Recovery	22
5 week follow up	22
3 month follow up	22
6 monthly follow ups	22
Brain Donation at Natural Death	22
Safety and Adverse Outcomes	23
Adverse Events and Trial Progression Decisions	24
**Study 2: Open Label Therapeutic Efficacy Study**	**25**
References	26

Reversal of Canine Cognitive Dysfunction by Hippocampal Transplantation of Dog-specific Skin-derived Neural Precursors

Protocol: Version 8

28 August, 2013

Short title: The Dogs + Cells Trial

Team

Chief investigator:	A/Prof Michael Valenzuela
Trial coordinator:	Ms Ellen Rasidi (BScVet student)
Veterinary clinicians and academics:	Prof Paul McGreevy, Dr Georgina Child, Dr Katja Voss, Dr Heide Kloeppel
Neurosurgeon:	Dr Erica Jacobsen
Neuroimaging:	Mr Harry Hallock (MPhil student)
Cell culture:	Ms Aileen Lowe (PhD student)

Independent Veterinary Safety Review Committee

1. Prof Rosanne Taylor (Dean, Faculty of Veterinary Science)
2. Dr Simon Roberts (Veterinarian, Struggletown Animal Hospital)
3. Dr Gail McDowell (Veterinarian, Cannon and Ball Veterinary Surgeons)

Significance

Dementia currently affects over 26 million individuals, with Alzheimer's Disease the main underlying pathology. It is an incurable, insidious and ultimately terminal condition that produces enormous personal, social and economic burden. The ageing of modern society is set to only further magnify this issue, with forecasts of 100 million individuals affected worldwide by 2050 (Brookmeyer, Johnson et al. 2007). New strategies for definitively treating Alzheimer's dementia are hence urgently required, as currently, cholinesterase inhibitor medication merely temporarily halts decline in about 30% of patients for a maximum of one year.

This proposal is based on the assumption that dementia is a disease of synaptic and neuronal loss (Scheff and Price 2003; Palop and Mucke 2010). Cell therapy aims to replace lost synaptic and neuronal populations through the introduction of neural stem cells and neural precursor cells into the diseased brain, and through both chaperone effects and synaptic integration, restore the information processing capabilities of distributed neuronal networks (Valenzuela, Sidhu et al. 2007).

A number of studies have indeed shown that NSC therapy can reverse cognitive deficits in different rodent models of Alzheimer's disease and neuronal loss (Qu, Brannen et al. 2001; Yamasaki, Blurton-Jones et al. 2007; Tang, Xu et al. 2008; Blurton-Jones, Kitazawa et al. 2009). However, none of these studies have used a clinically feasible cell type by deriving cells from a patient-specific tissue source, and hence leaves open the question of possible immune rejection and the need for immune suppression (itself toxic to transplanted cells). Also, studies of cell therapy have been limited by high rates of glial differentiation of cells *in vivo*, despite often showing high rates of neuronal differentiation *in vitro* (Roy, Cleren et al. 2006).

In response, we have developed a neural precursor technique that is patient-specific (being based on adult *canine* skin) and that also produces neuronally-restricted cells both *in vitro* and *in vivo* (as will be detailed below). At the same time, we have developed and validated a system for diagnosing and assessing **Canine Cognitive Dysfunction** (CCD) in older dogs, a syndrome with many important parallels with human dementia and hence of translational significance to human health (as also detailed below).

Aims

Our overall objectives are therefore to carry out two inter-connected studies in a group of older dogs with Canine Cognitive Dysfunction:

Study I: Test the safety of the intervention through serial veterinary assessment.

Study II: For the first time, test the efficacy of intra-hippocampal transplantation of skin-derived neural precursors for the reversal of CCD symptoms.

Background

Canine Cognitive Dysfunction (CCD) is a valuable model of human Alzheimer's dementia on the basis of clinical, epidemiologic, pathological and pharmacological similarities. Our group has developed the *Canine Cognitive Dysfunction Rating* scale (CCDR), a diagnostic and severity rating instrument that asks owners about the presence and longitudinal course of amnestic, disorientation, abnormal locomotor, agitation, apathetic and house-soiling behaviours (Salvin, McGreevy et al. 2010). These have clear parallels with human dementia symptoms (Hughes, Berg et al. 1982), and by using our CCDR definition

we have found that approximately 10% of dogs aged 8-10 years express the disorder, rising to 20% in dogs aged 14 years and over (Salvin, McGreevy et al. 2010). This age-related exponential increase in prevalence resembles findings from human epidemiological dementia studies (Jorm, Korten et al. 1987). Canine brain ageing is also accompanied by development of Alzheimer pathology such as amyloid plaques (Head, McCleary et al. 2000), and furthermore, the canine response to candidate dementia drugs is more predictive of human clinical outcomes in comparison to rodent studies (Studzinski, Araujo et al. 2005).

For these reasons, we are interested in testing the merits of cell therapy for the reversal of CCD as a necessary translational step prior to possible human clinical trial. Towards this goal, our group has developed a new protocol for generating neurons from adult canine skin (Valenzuela, Dean et al. 2008). Starting off with a small 8cm^3 skin sample, our procedure involves mechanical and enzymatic breakdown of the tissue, induction of primary neurospheres, and propagation in an adherent monolayer system supported by defined growth factors (**Figure 1**). Within three weeks of isolation, we can generate over 1million skin-derived neural precursors cells for transplantation purposes. Such adult canine *Skin-derived Neural precursors* (SKNs) are neuronally-restricted *in vitro*, maturing into a homogenous population of neurons as defined by a panel of neuronal protein markers (e.g., *MAP2*, *NF*, *Beta-tubulin*) (Valenzuela, et al. 2008).

Figure 1. A) *Canine-skin derived neurospheres (primary passage)* ***B)*** *Canine skin-derived neuroprecursors (SKNs) in adherent monolayer culture during propagation* ***C)*** *Canine SKNs following 28DIV differentiation showing multipolar neurite morphology. From Lowe et al (in prep).*

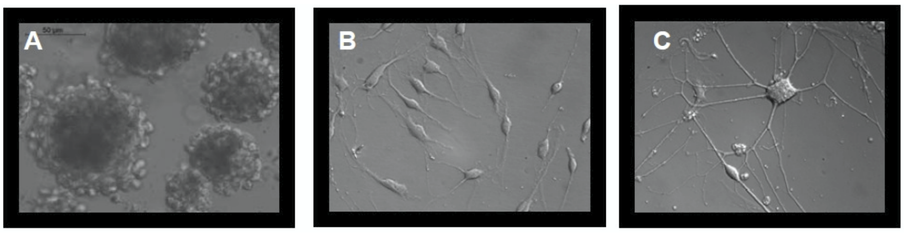

One of the major limitations of previous transplantation studies of stem cells into the rodent brain has been a bias to differentiate into glial cells *in vivo* (Roy, Cleren et al. 2006; Blurton-Jones 2009). By contrast, our adult canine SKN cells do not display glial (*GFAP*) mRNA or protein (**Figure 2**), and hence are theoretically incapable of differentiating into glial cells *in vivo*.

Figure 2. *Comparative results of canine skin-derived versus canine brain-derived neuroprecursors after 28DIV differentiation. SKNs express a range of neuronal proteins, but no glial GFAP; brain-derived cells express both neuronal proteins as well as glial GFAP. PCR replicated these cell line differences on gene expression (P: Proliferation, D: Differentiation, +: positive control canine brain homogenate, --: negative control (fibroblasts), -: negative control (water). From Lowe et al (in prep).*

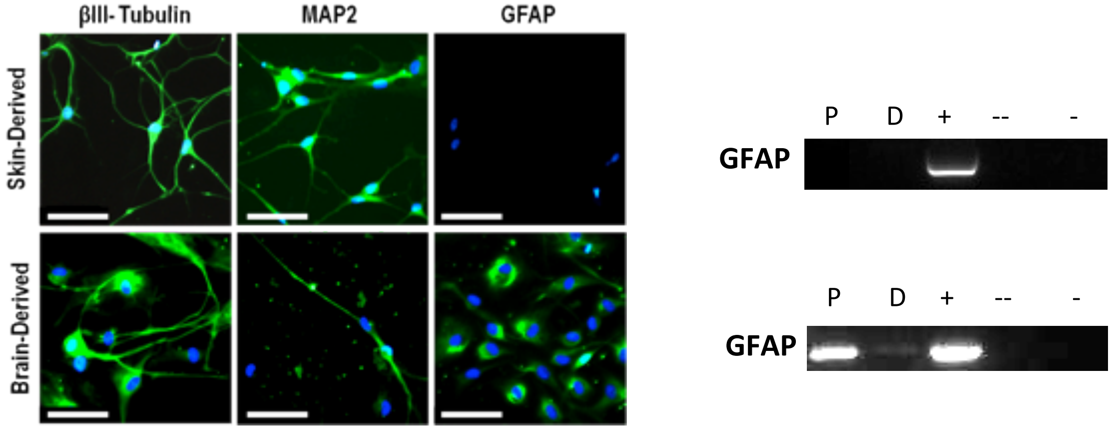

Most recently, we have carried out a proof-of-concept cerebral transplantation study of adult *canine* SKNs into an aged *rodent* model of memory dysfunction. Aged rats naturally exhibit dysfunction in place recognition memory, despite preserved object recognition memory, a phenotype highly dependent on synaptic integrity in the hippocampus. Transplantation of ~200,000 adult canine SKNs bilaterally into the aged rodent hippocampi restores place recognition memory back to performance levels seen in young adults (**Figure 3**). ***Using a clinically more meaningful outcome measure, 50% of initially memory-impaired older rats categorically change to a non-impaired state 7 weeks after transplantation*** (see **Appendix A** for individual animal results).

Figure 3. *A new object in a familiar location triggers spontaneous preferential exploration in both young and aged (20-month) wild-type Fischer rats equally in the Object Recognition Memory test. By contrast, aged rats are specifically impaired on the Place Recognition Memory test, where a familiar object has been placed in a new location. SKN hippocampal transplantation rescues place recognition memory in aged animals back to levels seen in young animals. Sham surgery (with acellular media) has no effect. From Siette et al (in prep).*

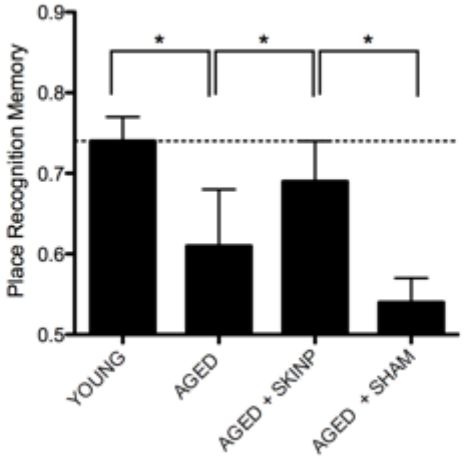

Furthermore, cells migrate within the hippocampus to engraft into the subgranular dentate gyrus and CA1 subfields (**Figure 4**). Confocal immunofluorescent double-staining confirms that transplanted cells almost universally differentiate into neurons *in vivo (***Figure 4***)*.

From these rodent studies we have primary evidence that the intervention may be safe: no transplanted rodents have died prematurely, and up until the 8-week post transplantation time point, we have not found evidence of teratoma or tumor formation. See **Appendix A** for individual rodent data.

This proposal therefore aims to translate our preclinical rodent findings to CCD in older dogs, a key bridging step between rodents and humans. Aged dogs with CCD as identified using our CCDR clinical scale will have a skin sample harvested, SKNs grown and amplified *in vitro*, and then transplanted into the bilateral hippocampi under anaesthesia and stereotaxtic MRI guidance. Efficacy would be assessed through repeat CCDR administration, as well as additional memory tests developed by our group.

If successful, this outcome would represent the first evidence for reversal of a dementia-like syndrome in a naturalistic animal model. Results from this study may therefore also stimulate translation to clinical trial of human SKN transplantation for the curative treatment of Alzheimer’s Dementia.

Figure 4. ***A)*** *Hippocampal dentate gyrus of transplanted 18-month rat, showing engraftment of canine SKN cells (canine-specific Lamin, brown) in subgranular zone,* ***B)*** *Engrafted cells (brown) in CA1 hippocampal subfield, showing organized pyramidal structure and development of dendritic projections* ***C)*** *Double immunofluorescent labeling shows CA1 engrafted cells (yellow) are uniformly positive for neuronal protein beta-tubulin (red, D) as well as canine-specific Lamin (green, D). Hence, our SKN cells exhibit near-universal neuronal differentiation in vivo as predicted by in vitro studies. From Siette et al (in prep).*

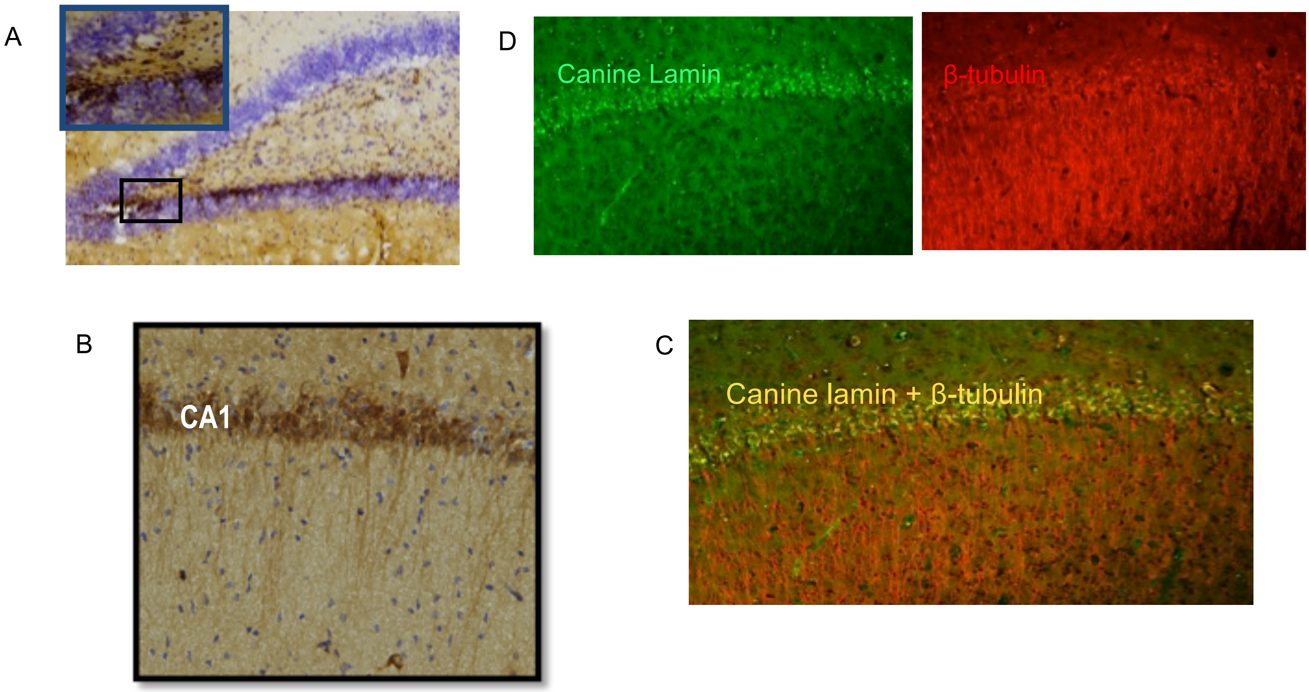

Study 1: Dogs + Cells Safety Study

Aim

To assess safety of protocol in N=3 older dogs with CCD and observe any potential clinical responses.

Overview of Procedures & Timeline

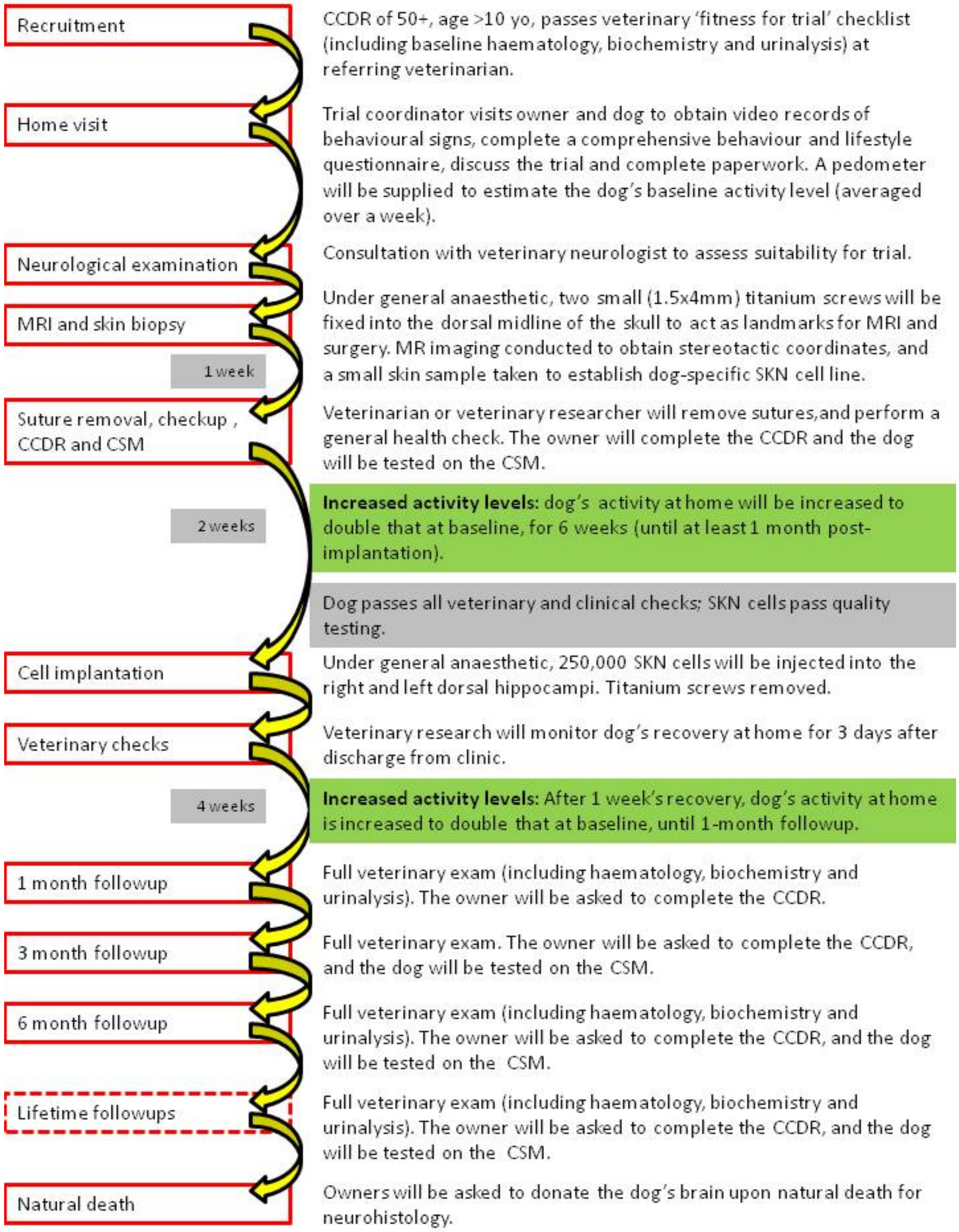

Recruitment

1. Use of the www.maturedogs.com *CCDR register*. At October, 2011 the register has the CCDR scores of 410 aged dogs with the average score being 45.14 out of 80. We will contact by email those owners of dogs with a CCDR scores in the range required (50-65).

2. Involvement of local veterinary clinics. Between March-April 2013, 20 veterinary clinics in the inner west and eastern suburbs of Sydney were visited by the trial coordinator, to discuss the syndrome of CCD, the trial, and the opportunity to refer eligible patients was offered. These clinics now form a mailing list of clinicians to whom updates about the trial are sent on a bi-monthly basis.

3. Promotion of the trial to veterinarians through professional publications and seminars. These have included short articles in the NSW Division of the Australian Veterinary Association (AVA) email newsletter, a full-page article in the Autumn 2013 issue of the Australian Small Animal Veterinary Association (ASAVA)'s publication *Companion*, presence at the Centre for Veterinary Education (CVE)'s seminar *Geriatric Moments* and national media associated with a special presentation at the Alzheimer's Australia national conference in Hobart, May 2013.

Selection criteria

1. Age ≥ 8 years
2. CCDR score over 50
3. No severe separation anxiety
4. Veterinary assessment
    a. Normal blood panel including T4
    b. Normal urinalysis
    c. Reasonable level of mobility
    d. Body condition score ≤4 (not obese)
    e. Reasonable level of vision
    f. No other likely causes of cognitive impairment
5. Not on high-dose oral corticosteroids.
6. Passes veterinary ‘fitness for trial’ checklist

Veterinary "fitness for trial' checklist

Owner's name:__________________________________

Dog's name:____________________________________

Dog's age:_____________________________________

Medical history

Has this dog had any diseases/illnesses that may preclude it from inclusion in this study?

No ☐ Yes ☐ Details:______________________________

Has this dog previously had any adverse reactions to anesthetics or surgery?

No ☐ Yes ☐ Details:______________________________

Does this dog have any diseases/illnesses that may increase the risk of a surgical procedure?

No ☐ Yes ☐ Details:______________________________

Current medical status

Does this dog have an abnormal T4 level?

No ☐ Yes ☐ Details:______________________________

Does this dog have any other abnormal blood results?

No ☐ Yes ☐ Details:______________________________

Does this dog have any abnormal urinalysis results?

No ☐ Yes ☐ Details:______________________________

If yes to any of the above, are these results indicative of any disease/illness that may cause cognitive impairment of CCD-like symptoms?

NA ☐ No ☐ Yes ☐ Details:______________________________

Please rate this dog's level of eyesight:

Very bad ☐ Bad ☐ Borderline ☐ Reasonably good ☐ Normal ☐

Please rate this dog's degree of movement/ability to get around:

Very bad ☐ Bad ☐ Borderline ☐ Reasonably good ☐ Normal ☐

Using the body condition score chart provided, what body condition score is this dog?

1. Very thin ☐ 2. Thin ☐ 3. Ideal ☐ 4. Overweight ☐ 5. Obese ☐

Suitability for inclusion in this study:

Do you believe this dog is physically fit enough to undertake anesthesia and brain surgery?

No ☐ Yes ☐

Details:____

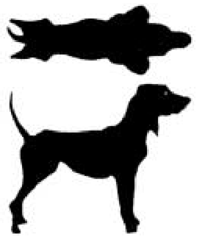

1 Very Thin

Ribs	Easily felt with no fat cover
Tail base	Bones are raised with no tissue between the skin and bone
Side view	Severe abdominal tuck
Overhead	Accentuated hourglass shape

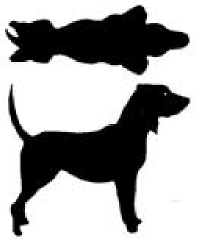

2 Thin

Ribs	Easily felt with no fat cover
Tail base	Bones are raised with minimal tissue between the skin and bone
Side view	Abdominal tuck
Overhead	Marked hourglass shape

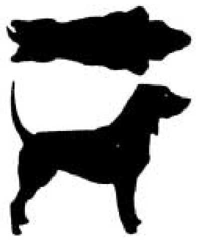

3 Ideal

Ribs	Easily felt with slight fat cover
Tail base	Smooth contour but bones can be felt under a thin layer of fat
Side view	Abdominal tuck
Overhead	Well proportioned lumbar waist

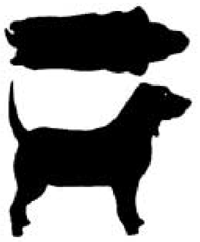

4 Overweight

Ribs	Difficult to feel with moderate fat cover
Tail base	Some thickening but bones can be felt under a moderate layer of fat
Side view	No abdominal tuck or waist
Overhead	Back is slightly broadened

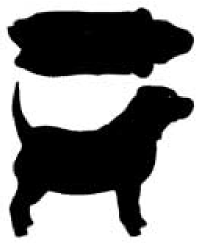

5 Obese

Ribs	Difficult to feel under thick fat cover
Tail base	Thickened and difficult to feel under a prominent layer of fat
Side view	Fat hangs from the abdomen and there is no waist
Overhead	Markedly broadened

Initial screening
The dog's primary caregiver will fill out our Canine Cognitive Dysfunction Rating scale (CCDR), either online or through a referring veterinarian, in order to determine if the dog may be suitable for the study. Participants referred to the trial by their veterinarian will have a veterinary examination, as well as routine blood tests and urinalysis performed. Participants coming to the trial via other channels will have these tests performed at the time of neurological exam at the UVTHS.

Home visit
Definitive pre-treatment baseline assessment. The trial coordinator will arrange to visit the primary caregiver and the dog in the home to discuss the trial protocol, answer questions and concerns and complete the required consent form. In addition, the coordinator will discuss the dog's CCDR results, to video any behavioural signs associated with CCD and to gather behavioural and lifestyle data via a standardised questionnaire. We will also provide you with a pedometer (attached to a collar) to measure your dog's baseline level of exercise. At this point we will inform you whether your dog is definitively suitable for the study.

Baseline physical activity
Obtain baseline levels of physical activity using a pedometer on the dog's collar for a 1 week period. Owners are asked not to change from their dogs normal exercise routine.

Neurological exam
The primary caregiver and dog will attend a consultation with the Dr Georgina Child, Veterinary Neurologist, at the UVTHS for a full neurological examination, to confirm the CCD diagnosis and to rule out other major neuropathologies.

MRI and skin biopsy
The dog will be admitted to the UVTHS and undergo three procedures under general anaesthetic:
i) a small (~1x3cm) skin sample will be taken from the periumbilical area of the ventral abdomen,
ii) the insertion of two very small (1x4mm) titanium screws into the sagittal (midline) ridge of the skull, which will serve as landmarks in MR imaging and during later surgery; and
iii) an MRI brain scan.
For these procedures the dog will be in the care of the specialist veterinary anaesthesia, surgical and imaging teams. The dog will remain under observation at the UVTHS for up to 24 hours, and will be discharged with analgesia and antibiotics if necessary.

Skin biopsy
A single biopsy per animal will be taken under sterile conditions at the UVTHS prior to MRI while under general anaesthesia. The procedure will be undertaken by a veterinary surgeon and requires the removal of approximately 1.5cm^2 full-thickness skin (~1x3x0.5cm) from the periumbilical area. The wound will closed and require a simple dressing. The skin sample will be immediately transferred into a specimen jar in DPBS solution and transported to A/Prof Valenzuela's Regenerative Neuroscience laboratory at the Brain and Mind Research Institute.

Screw fixation
The insertion of the screws require the dorsum of the dog's head to be shaved and surgically prepared,

with either one 4cm or two 2cm midline incisions dorsum of the head, depending on the size of the dog. The fascia covering the external sagittal crest will be incised and two 4mm deep holes will be drilled in the midline for the fixation of two 1.5 x 4mm titanium orthopaedic screws. The rostral screw will be located just caudal of the convergence of the two temporal lines at the sagittal crest, the other approximately 2-3cm caudal to this (depending on the size of the dog). The skin will be closed and the screws will remain in place until cell implantation surgery.

MRI and the calculation of intrahippocampal stereotaxic co-ordinates

Brain imaging will occur at the University Veterinary Teaching Hospital, Sydney. Before acquisition of the 3D T1 weighted MR image, a series of scout scans are performed to ensure the alignment of the head within the scanner. Dogs are scanned in sternal recumbency with the head extended. The coronal, sagittal and transverse planes are set in the MRI System software, but several scout scans are performed to ensure the head is aligned in the following way: that the ventral aspect of the tympanic bullae are horizontal (X-axis); that the line transecting the external sagittal crest and the midline of the brain between the two cerebral hemispheres is vertical (Z-axis), and that the line transects the insertion site of the two titanium screws at the level of the skull surface is horizontal (Y-axis).

Images are acquired by a 0.25 Tesla Esaote Vet Grande MRI System (Software release 9.2) with a gradient strength of 20mt/meter and a resonance frequency (RF) strength of 900 watts. All dogs will be positioned in sternal recumbency, using RF dual phase array C2 coil. Gradient Echo 3D T1W with TR/TE/Flip of 38/16/75, and FOV 26 x26, matrix 256 x 256, 3D FOV 130, 3D Phases= 128 with the resulting resolution Voxel of 1.02 x1.02x1.02mm with the brain positioned at the isocentre.

Images are obtained in coronal sequence and repositioned and resliced prior to implantation surgery using Analyze (Biomedical Imaging Resource) software. In this image processing, the auditory meatuses were aligned on the X-axis, as were the screw insertion sites at the level of the skull surface (Y-axis) and the image was resliced in the coronal plane.

The rostral of the two screw sites was used as the zero point. For each hippocampal target, the X- and Y-coordinates were calculated from the centre of the zero point, and the Z-coordinate calculated from the skull surface.

When the dog has fully recovered from anaesthesia, it will monitored at the UVTHS for up to 24 hours before being released back into the care of its owner with instructions for managing the dressing and the next part of the project.

SKN culture: Cell Culture Protocols

Isolation protocol (Modified from Valenzuela, 2008)

Upon arrival at the Regenerative neuroscience laboratory, tissue is washed with DPBS/PS, chopped into 1-2mm pieces, rinsed with DPBS/PS and finally incubated in 15ml of 0.1% Trypsin at 37C /5% CO2 for 40 minutes. At the end of the incubation period, solution is aspirated and 5mL of 0.1% DNAse 1 added for 1 minute at room temperature. Tissue is then washed with DPBS x 2, F-DMEM x 1 and DMEM-F12 3:1. Following the final aspiration, tissue is transferred to a glass petri dish and mechanically chopped until the pieces are tiny and form a paste-like consistency. Suspension is placed on mesh strainer over a collection vessels and tissue fragments forced through the mesh with a glass pestle. The suspension is poured through a 40um cell strainer, centrifuged at 1300rpm for 5 minutes, resuspend in fresh DMEM/F12 3:1 complete medium. A cell count is performed using a haemocytometer and 1x10^6 cells are added per well of a 6 well in 5mL of complete medium. Incubate at 37C / 5% CO2, spheres should start to form within 3-4 days. Culture is ready to be transferred to adherent culture when the majority of spheres are >50um in diameter.

Transferring to adherent culture

Coating of culture vessels with 0.1% Gelatin should occur at least 1 hour prior to passage time. Collect sphere suspension via centrifugation. Discard the supernatant, and add 1mL of pre-warmed TryPLE select. Manually titurate for 1 minute, incubate at 37C /5% CO2 for 5 minutes, followed by additional 1 minute tituration. Neutralise TryPLE using DMEM/F12 3:1, centrifuge at 1300rpm, count and seed cells at a density of 500 000 cells / T25 flask. Complete media change occurs every 3 days.

Passaging/ Continual culture

Flasks are ready for passage when 80% confluence is achieved. Coating of culture vessels with 0.1% Gelatin should occur at least 1 hour prior to passage time. Discard spent medium and add 2mL of prewarmed TryPLE select per T25 flask. Incubate for 2 minutes at 37C/5%CO2, gently tap sides of flask to dislodge cells, check under microscope to ensure majority of cells are detached. . Neutralise TryPLE using DMEM/F12 3:1, centrifuge at 1300rpm, discard supernatant, resuspend,count and seed cells at a density of 250 000 cells / T25 flask.

Loading into tube for transplant

As per passaging protocol above. Following centrifugation resuspend in 500ul of DMEM, perform a count and add 500 000 cells to a PCR tube. Keep a small aliquot aside for viability testing. Spin the PCR tube for 5 minutes at 1300rpm and resuspend in 2 x 25ul of PBS, transport immediately to transplantation location.

SKN culture: Timeline

Canine SKN EDU proliferation experiments conducted in 2009-2010 show decreased proliferation rates following the third passage (Figure 1). Passage data from 12 canine SKN isolations indicated an average time of approximately 22 days to reach the second passage (Figure 2). The optimal time for transplant should be post isolation day 28. This will maximise cell numbers and allow for appropriate quality control checks to occur.

Figure 1:

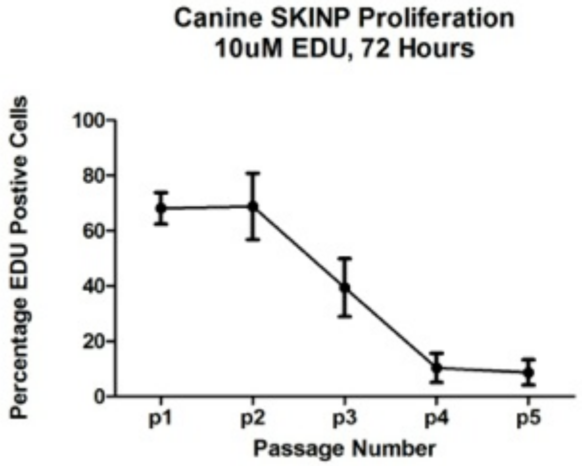

Figure 2:

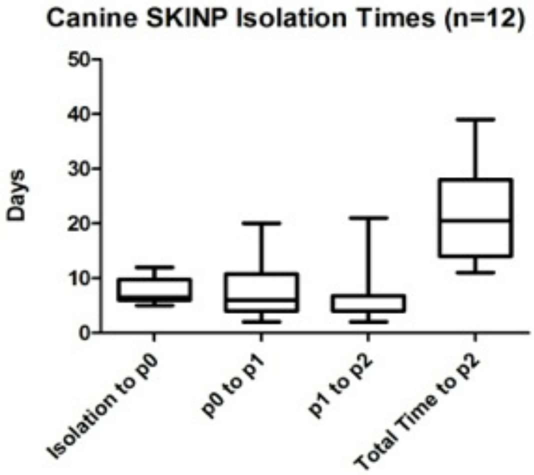

	Day 7	Day 14	Day 21	Day 28
	**P0**	**P1**	**P2**	**P3 / Transplant**
**Predicted Cell No**	-	1.25 x 10^6	2 x 10^6	1.5 x 10^6
**No of T25 Flasks**	2	4 ($1x10^6$)	3 ($7.5x10^5$)	
**Cryopreservation**		1 vial (2.5 $x10^5$)	1 vial (2.5 $x10^5$)	
**RNA**			2.4 $x10^5$ cells	
**Staining**			$1x10^5$	
**EDU Assay**			$6x10^4$	
**Trypan Blue Assay**				$2x10^3$

SKN culture: Quality Control Criteria / Summary Checklist

Criterion		
**Stage 1: Isolation**	**Yes**	**No**
Timing: Time between surgery and start SKN isolation < 2hrs?		
Size: Surface area (length x width) > 2 cm^2?		
**Stage 2: Neurosphere Culture (Day 5 Check)**	**Yes**	**No**
Contamination: All seeded wells contaminant free since isolation?		
Sphere Size 50-100um?		
**Stage 3: Adherent Culture (p0 pre-passage 1 check)**	**Yes**	**No**
Contamination: All seeded flasks contaminant free since isolation Day 5 Check ?		
Adherent culture >80% confluent?		
Absence of fibroblastic cells?		
Cell count < 500 000 / flask		
Time from P0 to P1 greater than 10 days?		
**Stage 4: Quantitative Gene Expression Profile (Following Passage 2) (Figure3)**	**Yes**	**No**
Relative expression of p75 gene greater than fibroblast control?		
Relative expression of Nestin gene greater than fibroblast control?		
**Stage 6: Protein Expression Immunocytochemistry (Following Passage 2) (Figure 4)**	**Yes**	**No**
Percentage of Nestin positive cells > 70%?		
Percentage of βIII-tubulin positive cells > 80%?		
**Stage 7: EDU Assay (Following Passage 2)**	**Yes**	**No**
Percentage EDU positive cells greater than 20%?		
**Stage 8: Trypan Blue Exclusion Assay (Transplant Day)**	**Yes**	**No**
Percentage of Live cells >85%?		

SKN culture: Quality Control Protocols

Trypan Blue Staining

In an eppendorf tube add 30ul of cell suspension to 30ul of Trypan blue dye. Using a haemocytometer count first the number of dead cells (blue stain) followed by the total number of cells. Record and calculate percentage viability. Ensure count is performed immediately following addition of Trypan blue.

EDU Staining

Adherent cultures are treated with 10um 5-ethynyl-2'-deoxyuridine (EDU, Invitrogen) for 72hours. Cells are fixed with 4% paraformaldehyde and visualised according to manufacturer's instructions. Counterstaining is performed with 1:5000 Hoecsht 33342 (Invitrogen) and coverslipped. Quantification is performed using CellProfiler cell analysis software.

Gene Expression Analysis

Total RNA is extracted from samples using illustra RNAspin Mini Isolation Kit (GE Healthecare) according to manufacturer's instructions. cDNA is synthesized from 10ng-1 µg of RNA using Superscript III RT First Strand Synthesis System (Invitrogen). PCR amplification was performed using Platinum *Taq* DNA Polymerase (Invitrogen). PCR products were analysed and visualised on 2% agarose gels (Invitrogen) containing SYBR green (Invitrogen).

Immunocytochemical Analysis (Adherent Cultures)

Cells for analysis are fixed with 4% paraformaldehyde (Sigma-Aldrich) in 0.1% PBS at room temperature for 15 minutes, washed 3 times with PBS and incubated with 0.1% Triton-X for permeabilisation. Cells are then incubated for 1 hour at room temperature in blocking solution containing 3% Donkey serum / 1% BSA in 0.1% PBS. For primary antibody incubation blocking solution with either <insert antibodies and concentrations> were incubated overnight at 4C. Cells are washed with PBS three times, followed by incubation in blocking solution containing a 1:500 dilution of Alexofluor 488 or 594. Cells are counterstained with DAPI mounting medium and coverslipped. Images analysis occurs via image capture using fluorescence microscope and quantification using Cellprofiler image analysis software.

Definitive baseline CCDR and CSM

The primary caregiver will return with the dog to the UVTHS 14 days after MRI and biopsy, for a physical check and removal of sutures. Owners will complete a modified CCDR with all questions relating to a change in the dogs behaviour altered to reflect a comparison with 3 months previously (rather than 6 months previously), to allow more frequent follow ups post surgery. The dog will be taken for CSM testing.

CONFIDENTIAL

Dog Name **Owner Name** **Date**

**Column score**	1	2	3	4	5		
	Never	Once a month	Once a week	Once a day	>Once a day		Score
How often does your dog pace up and down, walk in circles and/or wander with no direction or purpose?							
How often does your dog stare blankly at the walls or floor?							
How often does your dog get stuck behind objects and is unable to get around?							
How often does your dog fail to recognize familiar people or pets?							
How often does your dog walk into walls or doors?							
How often does your dog walk away while, or avoid, being patted?							

	Never	1-30% of times	31-60% of times	61-99% of times	Always		
How often does your dog have difficulty finding food dropped on the floor?							

	Much less	Slightly less	The same	Slightly more	Much more		
Compared with 3 months ago, does your dog now pace up and down, walk in circles and/or wander with no direction or purpose							
Compared with 3 months ago, does your dog now stare blankly at the walls or floor							
Compared with 3 months ago, does your dog urinate or defecate in an area it has previously kept clean (if your dog has never house-soiled, tick "the same")							
Compared with 3 months ago, does your dog have difficulty finding food dropped on the floor						**x 2**	
Compare with 3 months ago, does your dog fail to recognise familiar people or pets						**x 3**	

	Much more	Slightly more	The same	Slightly less	Much less		
Compared with 3 months ago, is the amount of time your dog spends active							

Baseline Canine Sand Maze

Revised protocol to that published in Salvin et al., 2011 reducing the overall nun learning trials to 12 (six each of 50/50 and buried trials). This reflects our experi the original 18 trials was too long and physically demanding for older dogs and t motivation and learning peaked at the 6th 50/50 or buried trial. Pilot testing in a (n=1) confirmed that the revised protocol was equally applicable. Approximate 3 hours per dog.

Acclimation and motivation trials

- n = 4
- food is placed on the surface at a point half way between two doors around the enclosure. A marker (flag) is place behind the reward.
- Dog enters once through each of the four doors in random order
- Time taken to find reward is recorded, the trial ending when the food is eate
- If the reward is not found in 90 sec, the dog is shown the reward and the tri

Learning trials

- n = 6 each of 50/50 and buried trials
- 50/50 trials
    - Food is placed in the same location as above, with the reward half in only 50% is showing

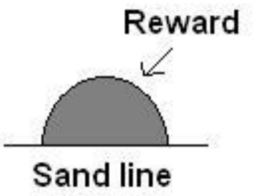

    - Time taken to find reward is recorded, the trial ending when the food
    - If the reward is not found in 90sec, the dog is shown the reward and ends
- Buried trials
    - Four food rewards are buried at a depth of 4cm, again in the sam The rewards are placed 20cm apart in a square formation.

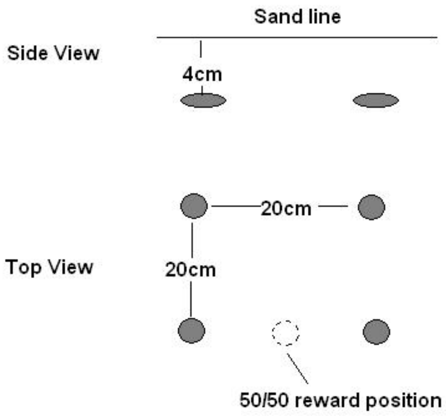

- Time taken to find reward is recorded, the trial ending when the food is eaten. Using the video footage, time taken to cross or enter the annulus (~1m^2 area around reward) is also measured
- If the reward is not found in 90sec, the dog is shown the reward and the trial ends

- 50/50 and buried trials are alternated, starting with a 50/50 trial and ending with a buried trial to give a total of n =12 trials
- The door used to enter the enclosure is randomly assigned but so that each door is used an equal number of times overall. That is - each door is used once in the 50/50 trials with two doors being used twice and once in the buried trials with two door being used twice (the doors not used twice in the 50/50 trials) giving a total of 3 uses overall, but the order they are used in is random.

Retention period
Dogs are given a 1½ hour break in which they are toileted and then allowed to rest quietly

Probe trial

- Four food rewards "dummy" are buried in the same way as the buried trials but the location is rotated a ¼ turn around the sand maze.
- Dogs enter through the door opposite their learned reward location and which requires them to walk past the dummy rewards
- The probe trial lasts for 90secs after which the trial ends and dogs are removed from the enclosure. The video footage is then used to measure the time taken to cross or enter the previously learned annulus.

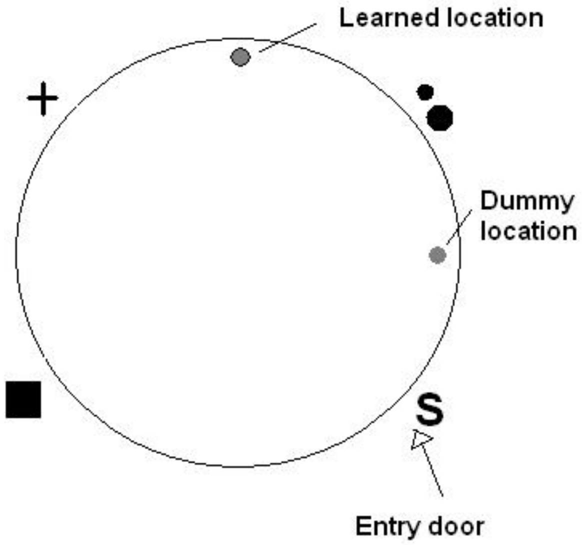

Period of increased physical activity
Owners asked to double their dog's baseline activity levels at home for a two week period prior to implantation surgery. Dogs will wear a pedometer on their collar for the two week period to measure compliance with the protocol.

Implantation surgery
Injection of 250,000 autogenic patient-specific SKN cells suspended in 20microL of phosphate buffered saline via fine needle localisation into each dorsal hippocampi using MR stereotaxic guidance (i.e, 500,000 cells in total; surplus cells will be characterized *in vitro*).

Syringe priming procedure for cell transplantation

SKN cells will be suspended in PBS. This procedure is designed to prime the syringe with vehicle fluid so that dead space in the needle is filled, hence allowing efficient drawing up of cell suspension without air bubbles.

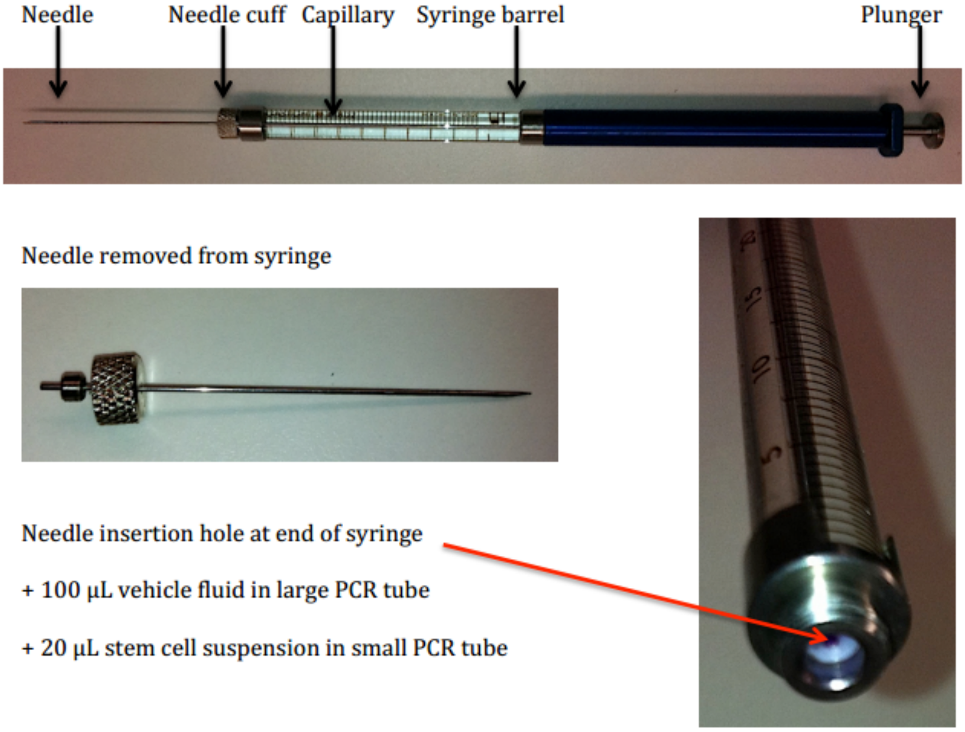

With needle fitted to syringe, draw up 25 µL vehicle fluid. Expel approximately 20 µL fluid, ensuring that syringe stopper is clear visible inside capillary. Undo needle cuff and remove needle from syringe. Needle is now filled with vehicle fluid. Expel all residual fluid from syringe by pushing plunger down to end point. Carefully re-insert back end of needle into insertion hole at end of syringe and tighten needle cuff. Can now draw up stem cell suspension directly without any dead space.

Pre-Op Preparation

Prior to surgery the dog will be kept in a comfortable and warm environment. Premedication will be determined by the attending veterinary anaesthetist.

Anesthesia

Anaesthetic procedures will be determined on a case-by-case basis by the attending veterinary anaesthetist. Nitrous oxide is to be avoided. IV fluid will be administered during surgery and in recovery.

Surgery

Under anaesthesia in the operating room, the dog's head will be fixated using a purpose-built stereotaxic frame, positioned in sternal recumbency. Care will be given to look after all pressure areas. All points of dissection will be determined by the previous MRI. The skin on the dorsal aspect of the head will be shaved and surgically prepared. A linear incision will be made in the coronal plane (from ear to ear), over the sites for the burr hole. The underlying muscle (*m. temporalis*) will be split along the direction of the fibres to minimize pain. Dissection should continue to the skull at which point a 3 mm burr hole can be made

through the skull. Using controlled positioning, a 25G needle can be passed through the brain to the depth required. Once in place, 20 microlitres of SKN/media solution will be injected over 2 – 3 minutes. The path of the needle will be planned to avoid the motor pathways and to comfortably access the hippocampus. This is made easier by the position of the hippocampus in the dorsal part of the lateral ventricle. Once injected the needle can be removed. The burr hole will not require closure due to its small size, but the muscle and skin overlying the burr hole will be closed as deemed appropriate by the surgeons. Recovery from anaesthesia will be under observation of the veterinary anaesthetic team and a dedicated veterinary nurse provided by the trial to ensure the dog is under constant observation overnight.

Analgesia

Intra- and post-operative analgesia will be prescribed as appropriate by the attending anaesthetist clinic veterinarians, and will include the use of S8 drugs as necessary. Non-steroidal anti-inflammatories (NSAIDs) will be administered post-operatively if renal and liver function are not compromised.

Post-op care within hospital

The dog will recover in a post-op unit for a minimum of 24 hours and until awake and drinking fluids. Dietary intake can be given according to the dog's needs. Analgesia will be given regularly for 24 hours. Early mobilization will be encouraged to assess neurological state. Any neurological issues will be monitored closely and, if necessary, imaging will be done to assess if this is due to a complication (e.g. bleeding).

Post-op care at home

The wound should be checked daily and protected from scratching (collar). Oral intake should be tested. There may be some pain related to eating as dissection of the muscles of mastication has occurred. Soft foods should be given for the first week post-operatively to minimize pain.

Recovery

Dogs are allowed 1 week to recover with only light exercise under veterinary direction. Following that owners are again asked to provide their dogs with double their baseline exercise levels for four weeks using the same program used in the period of increased physical exercise (see above). Dogs will wear a pedometer on their collar for the four week period to measure compliance with the protocol, owners will also be asked to continue keeping a diary of walks and play sessions.

5 week follow up

Dogs will undertake a veterinary assessment including blood panel, urinalysis and physical examination to assess their recovery from the procedure. Qualitative feedback from owners will be recorded.

3 month follow up

Dogs will undertake a **veterinary assessment** including blood panel, urinalysis and physical examination. Owners will also be asked to complete the **modified CCDR** rating scale. Dogs will also repeat the **CSM** again.

6 monthly follow up

Dogs will undertake a **veterinary assessment** including blood panel, urinalysis and physical examination. Owners will also be asked to complete the **modified CCDR** rating scale. Dogs will also repeat the **CSM** again.

Brain donation at natural death

Following natural death or a decision to euthanize made by the owner, the dog's brain will be removed, fixed and preserved by embedding in paraffin. Neurohistological analysis of Alzheimer pathology, SKN cell fate and any anomalous cell growth will then occur at AProf Michael Valenzuela's Regenerative Neuroscience Laboratory.

Safety and adverse outcomes

Definition of major adverse events potentially related to the study

- Any noticeable change in behaviour that appears distressing, uncomfortable or painful to the owner
- Any major Central Nervous System event such as a stroke, encephalitis, seizure or loss of consciousness
- Any neurologic deterioration including change in mobility and changes in vision and or hearing
- Any decline in cognitive function or neurologic status as a result of repeated or single general anesthetic
- Severe and ongoing lethargy or tiredness
- Ongoing loss of appetite or weight loss
- Major ongoing change to sleep pattern
- Major derangement of blood biochemistry or urinalysis
- Unexpected death of animal
- Any new intracranial tumour
- Wound infection

Definition of adverse events unrelated to study

As the dogs will be old (≥ 8 years), the incidence or deterioration of the following age-related disorders is unlikely to be related to the intervention:

- Arthritis
- Thyroid disease
- Heart disease
- Renal disease or the development of a non-incranial tumour
- Blindness, deafness and chronic disease

Definition of minor side effects not requiring reporting

- Tiredness or lethargy lasting less than 48 hours after surgery
- Change in appetite lasting less than 48 hours after surgery
- Head or jaw soreness lasting less than 48 hours after surgery
- Scratching at the wound site

Adverse events and trial progression decisions

If at **any stage** during the proposed research a **major adverse event potentially related to the study** occurs to a participating dog, AProf Michael Valenzuela will contact the *Independent Veterinary Safety Review Committee* within 48 hours in order to consider the appropriate course of action.
Actions may include: continuing the study as planned, modification of the protocol, temporarily halting the study until further information is available, or permanently stopping the trial.
If **no major adverse events potentially related to the study** occur during the course of the Safety Study (Study 1), the subsequent Open Label Therapeutic Efficacy Study (Study 2) will begin automatically.

In the even of any **major adverse events potentially related to the study** occurs during the Safety Study (Study 1), the *Independent Veterinary Safety Review Committee* will determine whether the Open Label Therapeutic Efficacy Study (Study 2) can continue as planned, requires modification, or is to be halted.

Study 2: Open Label Therapeutic Efficacy Study

Aim

Primary aim will be to assess potential therapeutic efficacy in N=9 older dogs with CCD. Further assessment of long term safety will be a secondary aim.

Procedures

Will be identical to those proposed for Study 1 (Safety Study), including reporting of safety and adverse outcomes.

Power calculations and sample size

Based on our results of categorical rescue of rodent age-related memory impairment in 50% of animals following SKN transplantation, we project that 50% of CCD dogs will undergo **clinically relevant therapeutic improvement** in the form of:

1. A significant decrease of CCDR rating scores to below 50 points, and
2. A significant decrease of Canine Sand Maze probe trial time-to-annulus to below 15 seconds.

Since CCD is invariably a progressive condition, we have assumed a conservative 10% spontaneous therapeutic recovery rate as our null hypothesis. Assuming a 50% therapeutic recovery rate in treated animals, **a sample size of N=9 yields 89% power** based on the non-parametric Chi-square procedure (controlling alpha = 0.05).

Data analysis

Intention-to-treat non-parametric Chi-square tests will be used to determine if the frequency of non-impaired animals at different follow-up time points based on either CCDR ratings or Canine Sand Maze results are significantly different to our null hypothesis.
In addition, we will calculate effect sizes (Cohen’s *d*) for CCDR and CSM within-subject change scores in order to better estimate samples sizes for future studies using sham-controlled cross-over design and repeated measures ANOVA analysis.

References

Blurton-Jones, M., M. Kitazawa, et al. (2009). "Neural stem cells improve cognition via BDNF in a transgenic model of Alzheimer disease." Proc Natl Acad Sci U S A **106**(32): 13594-13599.

Brookmeyer, R., E. Johnson, et al. (2007). "Forecasting the global burden of Alzheimer's disease." Alzheimers Dement **3**(3): 186-191.

Head, E., R. McCleary, et al. (2000). "Region-specific age at onset of β-amyloid in dogs." Neurobiology of Aging **21**(89): 96.

Hughes, C., L. Berg, et al. (1982). "A New Clinical Scale for the Staging of Dementia." British Journal of Psychiatry **140**: 566-572.

Jorm, A., A. Korten, et al. (1987). "The prevalence of dementia: a quantitative integration of the literature." Acta Psychiatrica Scandinavica **76**: 465-479.

Palop, J. J. and L. Mucke (2010). "Amyloid-beta-induced neuronal dysfunction in Alzheimer's disease: from synapses toward neural networks." Nat Neurosci **13**(7): 812-818.

Qu, T., H. Brannen, et al. (2001). "Human neural stem cells improve cognitive functions of aged brain." Neuroreport **12(6)**: 1127-1132.

Roberts, T., P. **McGreevy**, M **Valenzuela**. (2010). "Human Induced Rotation and Reorganization of the Brain of Domestic Dogs." PLoS One **5**(7): e11946

Roy, N., C. Cleren, et al. (2006). "Functional engraftment of human ES cell-derived dopaminergic neurons enriched by coculture with teleromase-immortalised midbrain astrocytes." Nature Medicine **12**: 1259-1268.

Salvin, H., P. **McGreevy**, et al. (2010). "Underdiagnosis of Canine Cognitive Dysfunction: A cross-sectional survey of older companion dogs." The Veterinary Journal **184**:277-281.

Salvin, H., P. **McGreevy**, et al. (2011). "The Canine Sand Maze (CSM): An appetitive spatial memory paradigm sensitive to age-related change in companion dogs." Journal of the Experimental Analysis of Behaviour. **95:**109-118

Salvin, H. E., P. D. **McGreevy**, et al. (2010). "The canine cognitive dysfunction rating scale (CCDR): A data-driven and ecologically relevant assessment tool." Vet J. **188**:331-336

Scheff, S. W. and D. A. Price (2003). "Synaptic pathology in Alzheimer's disease: a review of ultrastructural studies." Neurobiol Aging **24**(8): 1029-1046.

Studzinski, C. M., J. A. Araujo, et al. (2005). "The canine model of human cognitive aging and dementia: pharmacological validity of the model for assessment of human cognitive-enhancing drugs." Prog Neuropsychopharmacol Biol Psychiatry **29**(3): 489-498.

Tang, J., H. Xu, et al. (2008). "Embryonic stem cell-derived neural precursor cells improve memory dysfunction in Abeta(1-40) injured rats." Neuroscience Research **62**: 86-96.

Valenzuela, M., K. Sidhu, et al. (2007). "Neural stem cell therapy for neuropsychiatric disorders." Acta Neuropsychiatrica **19**: 11-26.

Valenzuela, M. J., S. K. Dean, et al. (2008). "Neural Precursors from Canine Skin: A New Direction for Testing Autologous Cell Replacement in the Brain." Stem Cells and Development **17**(6): 1087-1094.

Yamasaki, T. R., M. Blurton-Jones, et al. (2007). "Neural stem cells improve memory in an inducible mouse model of neuronal loss." Journal of Neuroscience **27**(44): 11925-11933.

THE UNIVERSITY OF SYDNEY

REGENERATIVE NEUROSCIENCE GROUP

Reversal of Canine Cognitive Dysfunction by cell therapy using autologous skin-derived neural precursors

Protocol: Version 1

10 June 2019

Short title: The Dogs + Cells Trial 2.0

Index **2**

Team 4

Independent Veterinary Safety Review Committee 4

Significance 5

Aims 5

Background 5

Design 9

Overview of Procedures Timeline 10

Recruitment 11

Selection criteria 11

Pre-trial screening 12

Trial enrolment 12

Tracking device hardware installation 12

Baseline physical activity 12

Neurological exam 13

MRI, skin biopsy and blood collection for biobanking 13

Definitive baseline CCDR scale 13

Definitive Radial Arm Maze 13

Period of increased physical activity 13

Cell implantation pre-operative procedure 13

Cell implantation surgery 14

Post-operative care 15

Recovery period 16

Period of increased physical activity 16

Follow-up assessments 16

Brain donation at natural death 17

Safety and adverse outcomes 17

Adverse events and trial progression decisions 18

Power and sample size 19

Statistical analysis plan 19

Funding 19

CCDR Scale (pre-treatment) 21

CCDR Scale (post-treatment) 22

CADES questionnaire 23

Dog Lifestyle and Behaviour Questionnaire 24

‘Fitness for Trial’ checklist 30

Body Condition Scoring Chart 31

Radial Arm Maze testing procedure 32

SKN culture 35

SKN quality control 38

Post-operative seizure management plan 40

References 41

Research Team

Chief Investigator:	Prof Michael Valenzuela
Trial Coordinator:	Dr Kaylene Jones
Veterinary Specialists and Academics: UVTHS	Dr Georgina Child, Dr Laurencie Brunel, Prof Paul McGreevy
Veterinary Specialists: ARH	Dr David Simpson & Dr Sarah Goldsmid
Neurosurgeon:	Dr Erica Jacobsen
Neuroimaging:	Prof Michael Valenzuela, Dr Marshall Dalton
Cell culture:	Dr Thomas Duncan, Dr Adam Johnson

Independent Veterinary Safety Review Committee

1. Prof Rosanne Taylor (Veterinarian, School of Veterinary Science, University of Sydney)
2. Dr Matthew O'Donnell (Veterinarian, Northern Illawarra Veterinary Hospital)
3. Dr Gail McDowell (Veterinarian, Cannon and Ball Veterinary Surgeons)

CONTEXT AND SIGNIFICANCE

Dementia currently affects nearly 50 million individuals worldwide, with Alzheimer's Disease the main underlying pathology. It is an incurable, insidious and ultimately terminal condition that produces enormous personal, social and economic burden. The ageing of modern society is set to only further magnify this issue, with forecasts of 131.5 million individuals affected worldwide by 2050 (Alzheimer's Disease International https://www.alz.co.uk/about-dementia). New strategies for treating Alzheimer dementia are hence urgently required, as currently, cholinesterase inhibitor medication merely temporarily halts decline in about 30% of patients for a maximum of one year.

This proposal is based on the assumption that Alzheimer dementia is a disease of synaptic and neuronal loss (Scheff and Price 2003; Palop and Mucke 2010). Neurorestorative cell therapy aims to replace lost synaptic and neuronal populations through the introduction of neural stem cells and neural precursor cells into the diseased brain, and through both chaperone effects and synaptic integration, restore the information processing capabilities of distributed neuronal networks (Duncan and Valenzuela, 2017).

A number of studies have indeed shown that NSC therapy can reverse cognitive deficits in different rodent models of Alzheimer's disease and neuronal loss (Qu, Brannen et al. 2001; Yamasaki, Blurton-Jones et al. 2007; Tang, Xu et al. 2008; Blurton-Jones, Kitazawa et al. 2009). However, none of these studies have used a clinically feasible cell type by deriving cells from a patient-specific tissue source, and hence leaves open the question of possible immune rejection and the need for immune suppression (itself toxic to transplanted cells). Also, studies of cell therapy have been limited by high rates of glial differentiation of cells *in vivo*, despite often showing high rates of neuronal differentiation *in vitro* (Roy, Cleren et al. 2006).

In response, we have developed a neural precursor technique that is patient-specific (being based on adult *canine* skin) and that also produces neuronally-restricted cells both *in vitro* and *in vivo* (as will be detailed below and in our publication – Duncan, Lowe et al, 2017). At the same time, we have developed and validated a system for diagnosing and assessing Canine Cognitive Dysfunction (CCD) in older dogs, a syndrome with many important parallels with human dementia and hence of translational significance to human health (as also detailed below).

SCIENTIFIC AIMS

The overall aim of this study is to continue and complete our Phase I/II clinical trial of autologous canine skin-derived neural precursor cells for the treatment of canine cognitive dysfunction. Specifically, our primary objectives are to:

i) Establish the safety of the treatment, and
ii) Evaluate the therapeutic efficacy of the treatment.

BACKGROUND

Canine Cognitive Dysfunction (CCD) is an important natural model of human Alzheimer's dementia on the basis of clinical, epidemiologic, pathological and pharmacological similarities [recent review paper by external group]. Our group developed the *Canine Cognitive Dysfunction Rating* scale (CCDR) as a diagnostic and severity rating instrument that asks owners about the presence and longitudinal course of amnestic, disorientation, abnormal locomotor, agitation, apathetic and house-soiling behaviours (Salvin, McGreevy et

al. 2010). The CCDR has been independently recommended for use in clinical research (Schutt, Toft et al, 2015), and the clinical signs evaluated have clear parallels with human dementia symptoms (Hughes, Berg et al. 1982). By using our CCDR definition we have found that approximately 10% of dogs aged 8-10 years express the disorder, rising to 20% in dogs aged 14 years and over (Salvin, McGreevy et al. 2010). This age-related exponential increase in prevalence resembles findings from human epidemiological dementia studies (Jorm, Korten et al. 1987). Canine brain ageing is also accompanied by development of Alzheimer pathology such as amyloid plaques (Head, McCleary et al. 2000), and furthermore, the canine response to candidate dementia drugs is more predictive of human clinical outcomes in comparison to rodent studies (Studzinski, Araujo et al. 2005).

For these reasons, we are interested in testing the merits of cell therapy for the reversal of CCD as a necessary translational step prior to possible human clinical trial. Towards this goal, our group has developed a new protocol for generating neurons from adult canine skin (Valenzuela, Dean et al. 2008; Duncan, Lowe et al, 2017). Starting off with a small skin sample, our procedure involves mechanical and enzymatic breakdown of the tissue, induction of primary neurospheres, and propagation in an adherent monolayer system supported by defined growth factors (**Figure 1**). Within three weeks of isolation, we can generate over million skin-derived neural precursors cells for transplantation purposes. Such adult canine *Skin-derived Neuroprecursors* (SKNs) are neuronally-restricted *in vitro*, maturing into a homogenous population of neurons as defined by a panel of neuronal protein markers (e.g., *MAP2*, *Beta-tubulin*) (Valenzuela, et al. 2008).

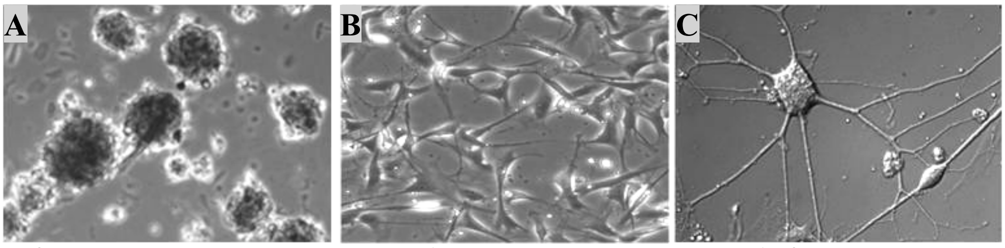

***Figure 1. A)** Canine-skin derived neurospheres (primary neurosphere passage) **B)** Canine skin-derived neuroprecursors (SKNs) in adherent monolayer culture during propagation **C)** Canine SKNs following 28DIV differentiation showing multipolar neurite morphology. Adapted from Duncan et al (in prep).*

One of the major limitations of previous transplantation studies of stem cells into the rodent brain has been a bias to differentiate into glial cells *in vivo* (Roy, Cleren et al. 2006; Blurton-Jones 2009). By contrast, our adult canine SKN cells do not display glial (*GFAP*) mRNA or protein (**Figure 2**), and hence are theoretically incapable of differentiating into glial cells *in vivo*.

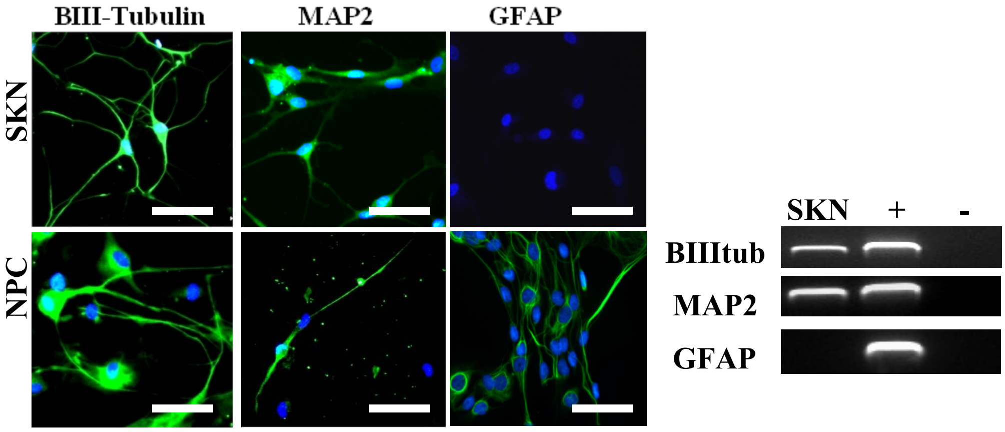

***Figure 2.** Comparative results of canine skin-derived versus canine brain-derived neuroprecursors after 28DIV differentiation. ISKNs express a range of neuronal proteins, but no glial GFAP; brain-derived cells (NPC) express both neuronal proteins as well as glial GFAP. PCR replicated these cell line differences with expression of mature neuronal genes BIIItub and MAP2, and no expression of*

glial cell GFAP gene in differentiated SKNs (+ control was canine brain tissue containing glial cells; - control was water). Adapted from Duncan et al (in prep).

In our pre-clinical rodent trial, we carried out a proof-of-concept cerebral xenotransplantation study of adult *canine* SKNs into an aged *rodent* model of memory dysfunction. Aged rats naturally exhibit dysfunction in place recognition memory, despite preserved object recognition memory, a phenotype highly dependent on synaptic integrity in the hippocampus (Siette, Westbrook et al, 2013). Transplantation of ~200,000 adult canine SKNs bilaterally into the aged rodent hippocampi restores place recognition memory back to performance levels seen in young adults (**Figure 3A**). ***Using a clinically more meaningful outcome measure, 50% of initially memory-impaired older rats categorically change to a non-impaired state 7 weeks after transplantation*** (see **Appendix A** for individual animal results). Furthermore, cells migrate within the hippocampus to engraft into the sub-granular dentate gyrus and CA1 subfields, where they differentiated into mature neurons (**Figure 3B and 3C**).

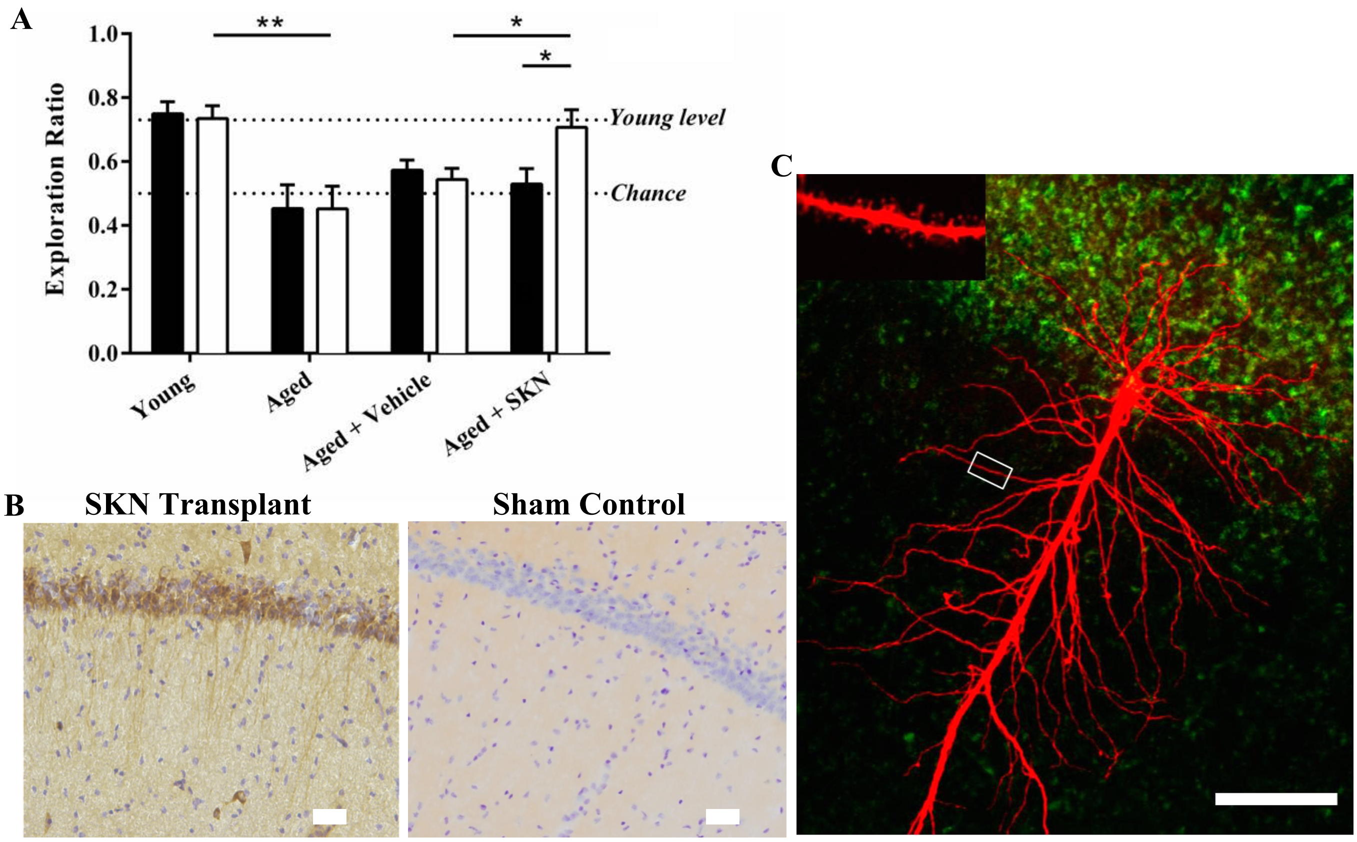

***Figure 3. A)** Aged rats are specifically impaired on the Place Recognition spatial memory test, where a familiar object has been placed in a new location (**p<0.01). SKN transplantation rescued spatial memory in aged rats back to levels seen in young animals. Sham surgery (with acellular media) had no effect (*p<0.05). B)* Transplanted SKNs (DAB stained brown) migrated extensively throughout the hippocampus, particularly populating the *CA1* subregion 10 weeks post-transplantation. (C) In this same region, at higher magnification a transplanted SKN cell labelled with biocytin (red) can be observed replicating the known anatomical architecture of CA1 pyramidal neurons, with a primary apical dendrite extending deep into the *stratum radiatum* and many perpendicularly-aligned secondary branching dendrites. Scale = 50µm. *Adapted from Siette PhD thesis 2012* (Siette J, 2012).

These rodent studies provided us with primary evidence that the intervention may be safe: no transplanted rodents died prematurely, and up until the 8-week post transplantation time point, we did not find evidence of teratoma or tumor formation. See **Appendix A** for individual rodent data.

This proposal therefore aims to translate our preclinical rodent findings to CCD in older dogs, a key bridging step between rodents and humans. Aged dogs with CCD as identified using our CCDR clinical scale will have

a skin sample harvested, SKNs grown and amplified *in vitro*, and then transplanted into the bilateral hippocampi under anaesthesia and MRI guidance. Efficacy will be assessed through repeat CCDR administration, as well as additional memory tests and behavioural tracking technology developed by our group. Safety will be assessed by longitudinal monitoring of general veterinary health and blood and urinary tests.

Prior DOGS+CELLS Trial Outcomes

The DOGS+CELLS trial has been running since 2012 under two previous USYD AEC approvals (2012 no. 5796 and 2016/1028). As summarized below, we have recruited into the trial seven animals, two of which were excluded prior to treatment, five have been treated, three have progressed to the primary endpoint evaluation at 3-months follow-up, and at this time, one animal has a pending endpoint in the near future. For the purpose of this application we assume our most recent patient, Grover, will reach the primary endpoint and therefore our prior trial work will deliver N=4 dogs with primary endpoints.

Name	Treated?	Reached primary endpoints? (3month post op)	Outcome
**Sasha 12YO, F Blue Cattle Dog**	Y	N	SAE neurosurgical complications requiring euthanasia
**Timmy 12YO, M Cocker Spaniel**	Y	Y	Unremarkable surgery and recovery. 2-year stable **clinical reversion of dementia**, client elected euthanasia for blindness.
**Leo 11YO, M Pomerian X Poodle**	Y	Y	Unremarkable surgery and recovery. 3-year + (ongoing) **clinical reversion of dementia**
**Foxie 16YO, F Miniature Fox Terrier**	N	N	Prior to treatment developed a gastric obstruction at home and client-elected euthanasia
**Grace 13YO, F Siberian Husky**	Y	Y	Unremarkable surgery and recovery. **No clinical response** (numerical improvement in CCDR within reliable change index) and client-elected euthanasia
**Macie 10YO, F Border Collie**	N	N	Pre-operative MRI found large pituitary adenoma and excluded from trial.
**Grover 12 YO, M Kelpie**	Y	ongoing	Unremarkable surgery and recovery.

Based on this data, intrahippocampal SKN therapy is proposed to be safe. The only treatment-related SAE was in the first dog patient (Sasha), after which a number of important protocol changes were implemented and no further safety issues have been observed in the subsequent four treated dogs.

Clinically, in the three dogs to reach our primary endpoint (spanning a follow up period of 3-months to 3-years) there has been no evidence of neurological impairment indicative of a brain tumor or rapidly enhancing intracranial mass. Further, in the single dog to reach primary endpoint *and* undergo brain PM

(Timmy), there was no evidence of abnormal cellular growth or proliferation. Hence, there is no evidence that our cell therapy poses a risk of long-term tumorigenesis or teratoma.

In terms of clinical efficacy, we do not yet have sufficient sample size to make definitive conclusions. The natural history of canine dementia is continuous decline to the point of client-elective euthanasia, and indeed all clients to date were considering euthanasia for their animal prior to the trial because of symptom severity. The categorical reversion of two out of three dogs to reach primary endpoint is therefore likely to be clinically meaningful, but the lack of response in the third dog is unexplained and possibly due to poor neuroanatomical targeting. **Testing clinical efficacy in an adequately-powered sample is therefore the over-riding scientific rationale for this application.**

Refinements introduced into this trial

This trial introduces two main technological innovations that serve to increase scientific integrity as well as improve animal welfare.

Firstly, intrahippocampal targeting of the SKN therapy will now occur in the USYD Hybrid Theatre, an advanced animal surgery facility that allows for intra-operative brain imaging, robotic surgical guidance and real-time visual confirmation that the needle tip is inside the hippocampus. Given there is a +/- 2mm tolerance for error in this regard, this is a major advanced compared to our previous trial that relied on a combination of fixation of fiduciary screws into the animal’s skull, customized anatomical calculations and stereotaxic instrumentation. Importantly, our patients will no longer require metallic screws to be implanted into their skull and there will no longer be any uncertainty around anatomical targeting.

Secondly, we introduce *Function Cloud* behavioural monitoring technology, that allows for continuous tracking of the patient’s position and movement trajectories in their home. This will produce objective and high resolution data and permit corroboration of owner’s clinical ratings.

DESIGN

This is a Phase I/II open-label veterinary trial. Following best practice, the design of the trial follows the PREPARE guidelines and results of the study will be reported following ARRIVE recommendations. Following AEC approval, the protocol will also be pre-registered with www.preclinical.eu.

Trial Procedures Timeline

Initial Screening	The dog's primary caregiver will fill out our Canine Cognitive Dysfunction Rating scale (CCDR), as well as a further set of screening questions, either online or through a referring veterinarian, in order to determine if the dog may be suitable for the study. Only dogs over the age of 8 years and scoring >50 on the CCDR will be considered.
Home Visit	The trial coordinator will arrange a home visit to discuss the results of the CCDR, observe the dog's behaviour and gather behavioural and lifestyle data via a standardised questionnaire.
Health Screening	The dog will have a veterinary examination, as well as blood tests and urinalysis performed by their regular veterinarian to further determine their suitability for the trial.
Official Trial Enrollment (0 weeks)	At this point the owner will be informed whether or not the dog is definitively suitable for the study. The trial coordinator will arrange a home visit to complete trial paperwork and to video any behavioural signs associated with CCD. A fitbark tag (pedometer) and a FunctionCloud tag (to track specific CCD behaviours in the home) will be attached to the dogs collar.
Tracking Device Set Up	A member of our research team will arrange a home visit to set up FunctionCloud tracking system hardware at the owners home (approx 1 hr).
Neurological Examination	The patient will attend a consultation with a Veterinary Neurologist at the University Veterinary Teaching Hospital Sydney (UVTHS). The dogs blood pressure will also be measured - in preparation of pre-GA check-list.
Skin sample and MRI (2-4 weeks)	Skin sample and MRI will be carried out under general anaesthetic at the ARH, Homebush. A blood sample will also be taken under GA for the purpose of biobanking for future CCD biomarker analysis. Patient-specific SKN cell line to be generated from skin sample in RNG laboratory.
Suture removal and baseline CCDR	10-14 days after the skin sample is taken, the trial coordinator will arrange a home visit to remove the sutures and to perform a general health check. The owner will be asked to complete the CCDR and CADES questionnaires again for definitive baseline result.
Radial Arm Maze Testing	The dog will be tested in the Radial Arm Maze.
	INCREASED EXERCISE: The dogs activity is increased to double that at baseline from 2 weeks prior to cell implantation.
Brain surgery and cell implantation (6-8 weeks)	Under general anaesthetic, approx. 250,000 SKN cells will be injected into the right and left dorsal hippocampi at the Hybrid Theatre, USYD.
Veterinary checks	The trial coordinator will conduct home visits to carry out post-operative checks on the patient each day, for 3 days following discharge from hospital.
	INCREASED EXERCISE: Following recovery from surgery (7-14 days), the dogs activity will be increased to double that at baseline, until 1 month follow-up.
Suture removal and check-up	10—14 days after the cell implant surgery, the trial coordinator will arrange a home visit to remove the sutures and perform a general health check.
1 month follow up	Full veterinary exam (including haematology, biochemistry and urinalysis). The owner will be asked to complete the CCDR and the CADES questionnaires.
3 month follow up	Full veterinary exam (including haematology, biochemistry and urinalysis). Blood also collected for biobanking. The owner will be asked to complete the CCDR and the CADES questionnaires. The dog will be also be tested in the Radial Arm Maze.
6 month follow up	Full veterinary exam (including haematology, biochemistry and urinalysis). Blood also collected for biobanking. The owner will be asked to complete the CCDR and the CADES questionnaires. The dog will also be tested in the Radial Arm Maze.
Lifetime yearly follow ups	Full veterinary exam (including haematology, biochemistry and urinalysis). The owner will be asked to complete the CCDR and the CADES questionnaires.
Natural death	Owners will be asked to donate their dog's brain upon natural death for histology.

4 weeks (Skin sample and MRI to Brain surgery and cell implantation)

PROCEDURES

Recruitment

1. Use of the Survey Gizmo online Canine Cognitive Dysfunction Rating Scale. http://www.surveygizmo.com/s3/1839821/Canine-Cognitive-Dysfunction-Rating-scale-CCDR.
   This scale is publicly available when searching the internet for information on canine cognitive dysfunction. It is also periodically included in publications released by veterinary organisations including: the Centre for Continuing Veterinary Education, The University of Sydney, the Australian Veterinary Association and the NSW Veterinary Practitioners Board. It is also periodically emailed to Veterinary Clinics in the Greater Sydney region.

2. Involvement of local veterinary clinics. All veterinary clinics in the Greater Sydney region have been contacted by email with information about the Dogs + Cells Trial. These clinics are now periodically updated with the progress of the trial and trial recruitment information. The Trial Coordinator also periodically presents information about the trial at Sydney based veterinary seminars.

3. Promotion of the trial to the public through the print and electronic media. To date articles have appeared in The Age newspaper, the Courier Mail and the Sun Herald regarding the success of two of the trial patients. Podcasts have been produced and aired on Vet Talk TV and the Channel 10 morning show has interviewed the owner of trial participant “Timmy”.

Selection criteria

1. Age ≥ 8 years
2. CCDR score of >50 – to be scored by the dog’s primary carer (ref pg. 21)
3. No severe separation anxiety
4. Veterinary assessment
   a. Normal blood panel including T4, triglycerides and coagulation profile *
   b. Normal urinalysis, including culture and sensitivity *
   c. Reasonable level of mobility
   d. Body condition score ≤4 (not obese) (ref pg. 25)
   e. Reasonable level of vision
5. No untreated medical, neurological or primary behavioural condition that may present with similar clinical signs to those of CCD.
6. Not on high-dose oral corticosteroids.
7. Passes veterinary ‘fitness for trial’ checklist (ref pg. 24)

*Please note that inclusion of dogs with mild abnormalities in their blood panel and/or urinalysis will be considered on an individual basis and at the discretion of the trial Veterinarian. The dogs which will be participating in this trial will be aged dogs (many of which will be 14 years of age or older). It is therefor probable that these dogs may have co-morbidities and age-related changes in their blood panel. If these abnormalities in blood/urine tests are not indicative of a disease process with clinical signs that may affect cognition, and are only mild in nature, then these dogs will be considered as potential candidates for the trial. Any potential risks posed by such blood abnormalities such as mild renal insufficiency will be explained to the owner and they will be fully understanding of any such associated risks.

Dogs with a history of metabolic or endocrine disease must have been stable under treatment for the preceding six month period and must have had normal blood results and no clinical signs of the disease for the duration of this period.

Initial Screening

The dog's primary caregiver will fill out our Canine Cognitive Dysfunction Rating scale (CCDR) as well as a further set of screening questions, either online or through a referring veterinarian, in order to determine if the dog is suitable for the study.

Initial Home Visit

The trial coordinator will arrange to visit the owner's home to discuss the results of the CCDR, observe their dog's behaviour and gather behavioural and lifestyle data via a standardised questionnaire.

Pre-trial Screening Tests

The dog will have a physical examination, as well as blood tests (including a CBC, biochemistry panel with T4, triglycerides and coagulation profile) and a urinalysis (urine to be collected via cystocentesis and culture and sensitivity to be included in analysis) performed by their regular veterinarian to further determine their suitability for the trial.

Trial Enrolment (time point = 0 weeks)

At this point, the owner will be informed whether or not their dog is definitively suitable for the study.

The trial coordinator will arrange to visit the owner's home to video any behavioural signs associated with CCD and to provide the owner with two small tags (to be attached to the dog's collar) to track and measure the dog's movements around the home.

Tracking Device Hardware Installation (0-2 weeks)

One of our researchers will visit the owner's home to place approximately 8 small, temporary beacons to the walls of the home. No permanent change to the owner's home will be involved. The anchors and power points will simply be attached to the walls of the home using double-sided sticky tape or blu-tack.

Baseline physical activity (0-2 weeks)

Obtain baseline levels of physical activity (using the Fitbark pedometre). Owners are asked not to change from their dog's normal routine during this period.

Neurological examination (0-2 weeks)

The primary caregiver and dog will attend a consultation with Dr Georgina Child, Veterinary Neurologist, at the UVTHS for a full neurological examination.

Skin Biopsy, brain imaging (MRI) and blood sample collection

The dog will be admitted to the ARH, Homebush where it will undergo 3 procedures under general anaesthesia:

i) a small (~2x8cm) skin sample will be taken from the periumbilical area of the ventral abdomen and

ii) a MRI brain scan will be performed.

iii) whilst the patient is under anaesthesia for the above procedures, a blood sample will be collected for the purpose of future biomarker analysis.

For these procedures the dog will be in the care of the specialist veterinary surgical and imaging teams. The dog will remain under observation at the ARH Homebush for up to 24 hours and will be discharged with analgesia if necessary.

Skin biopsy

A single skin biopsy will be taken under sterile conditions at the ARH homebush, prior to MRI while under general anaesthesia. The procedure will be undertaken by a specialist veterinary surgeon and requires the removal of approximately 2x8cm full thickness skin sample from the periumbilical area. The wound will be closed with non-absorbable monofilament sutures which will then be covered by a simple dressing. The skin sample will be immediately transferred into a specimen jar in DPBS solution and transported to Prof Valenzuela's Regenerative Neuroscience laboratory at the Brain and Mind Centre.

MRI

Brain imaging will occur at the ARH Homebush. MRI scans are acquired at 1.5 Tesla Siemens Magneton Avanto system. Prior to acquisition of the 3D T1 weighted MR image, a series of scout scans are performed to ensure the alignment of the head within the scanner. Dogs are scanned in sternal recumbency with the head extended and images are acquired in the coronal plane.

1. Localizer
2. MPRAGE-t1-3d-08iso-precontrast
3. t2-de3d_we_cor_320_1.5mm
4. Space-3d-T2-1mm-iso

MRI parameters include: RF dual phase array C2 coil, gradient Echo 3D T1W with TR/TE/Flip of 38/16/75, and FOV 26 x26, matrix 256 x 256, 3D FOV 130, 3D Phases= 128 with the resulting resolution Voxel of 1.02 x1.02x1.02mm with the brain positioned at the isocentre.

The MRI serves multiple purposes:

- To visualize the hippocampus for neurosurgical targeting
- To co-register with CT data from the Hybrid Theatre to permit image-guided micro-injection of therapeutic cells in the hippocampus.

- To rule out a non-degenerative cause of CCD symptoms ie. intracranial neoplasia, hydrocephalus, quadrigeminal cyst etc.

When the dog has fully recovered from anaesthesia, it will be monitored at the ARH for up to 24 hours before being released back into the care of the owner with instructions for managing the dressing and the next part of the project. The patient will be discharged with oral analgesia to be administered at home for 2-3 days following the skin biopsy procedure.

Blood collection for biomarker analysis

Approximately 12ml of blood is collected by jugular venipuncture into standard 6 X polypropylene EDTA test tubes followed by centrifugation. Plasma from 3 tubes is to be split into 500 µL aliquots and frozen and stored at −80 °C. Plasma from 3 tubes is to be split into 500 µL aliquots and frozen and stored in liquid nitrogen. Samples are to remain unthawed until analysis. (Lewczuk, Ermenn et al, 2018).
Blood will also be collected, as per the above protocol, at the 3 month and 6 month follow-ups. Sedation may be used if required, to prevent stress to the patient. (If the patient is relaxed, sedation will not be used).

Suture removal and definitive pre-treatment/baseline CCDR

The Trial Coordinator will conduct a home visit 10-14 days after MRI and biopsy to carry out a physical examination of the dog and remove the sutures. The primary care giver will complete the CCDR and CADES questionnaires which will be considered to be the reference baseline scores prior to treatment. The CCDR result will be used as the definitive baseline endpoint for therapeutic efficacy. The CADES will be used as a secondary measure.

Definitive pre-treatment/baseline Radial Arm Maze (RAM)

The dog will be tested using our Radial Arm Maze at the University of Wollongong's Ecological Research Centre. The Radial Arm Maze takes approx 2 1/2 hours (including a 1 ½ hour rest period). It entails no painful or adverse stimuli; rather it involves testing whether dogs can remember the location of a hidden food treat (see RAM protocol attached p.31)

Period of increased physical activity

Owners are asked to double their dog's baseline activity levels at home for a two week period prior to implantation surgery. We will measure compliance with the protocol using data collected via the Fitbark device attached to the dog's collar.

Prophylactic anti-seizure medication

To reduce the risk of post operative seizures, the dog will receive levetiracetam for 48hrs pre-operatively. This medication will be continued for one week post operatively.

Admission to the UVTHS

The patient will be admitted to the UVTHS at approximately 7:30am on the day of the cell transplant procedure. The patient will be admitted by Dr Laurencie Brunel. A physical examination will be carried out

and an admission check list completed. The patient will then be transported to the Hybrid theatre in the UVTHS patient transport van, accompanied by the trial coordinator.
Please see attached Admission check list.
NB: The patient will be identified (clearly marked on monitoring sheets and cage cards) as being part of the research trial and under the care of Dr Brunel (or another vet with suitable clinical experience and assigned to the case by Dr Brunel) at all times whilst at the UVTHS.

Pre-Operative Preparation and Anaesthesia

Following admission to the Hybrid Theatre, the patient will have an IV catheter placed and IV fluid administration will commence. Pre-operative sedation will be administered (drugs and dosages to be determined by the attending Veterinary Anaesthesia Specialist). The dog will then be anaesthetised and the surgical site prepared. The patient will then be anaesthetized and transferred to the surgical theatre.
NB: Anaesthesia procedures will be determined on a case-by-case basis by the attending Veterinary Anaesthesia Specialist.
Please refer to attached Hybrid Theatre Anaesthesia SOP for further detail.

Peri-operative Medication

Peri-operative analgesia will be prescribed as appropriate by the attending Veterinary Surgeon and will include the use of S8 drugs as necessary. Intravenous antibiotics will be administered peri-operatively. Levetiracetam (anti-seizure medication) will be administered either SC or IV on the morning of the procedure, at the time of pre-operative sedation and repeated every 8 hours until the patient has recovered and oral administration can be re-commenced.

Neurosurgery

The patient will be positioned on the surgery table in sternal recumbency. The head will be put in a fixed position using a canine-specific stereotaxic frame (KOPF model 1504). Care will be taken to ensure that excessive pressure is not applied by the frame to points of contact with the dog's head. A longitudinal, midline incision (approx 6cm in length) will be made using a scalpel blade along the sagittal crest. Dissection will continue to the point of the skull. Two small burr holes (3mm diameter) will then be made through the skull using cranial drill, at the locations indicated by the cross hairs of the C-arm laser, either side of the midline in order to target each hippocampus.

Using controlled positioning, a 25G needle will be passed through the brain to the depth required (as determined by MRI-CT imaging). Once in place, 20 microlitres of SKN cell + media solution will be injected over 2 – 3 minutes. The path of the needle will be planned to avoid the motor pathways and to comfortably access the hippocampus. This is made easier by the location of the hippocampus in the dorsal region of the lateral ventricle. Once the solution is injected, the needle remains stationary for another 3-minutes, and is then removed. The burr hole will not require closure due to its small size. The muscle, sub cutaneous tissue and skin overlying the burr hole will be closed as deemed appropriate by the surgical specialist.

Post Operative Care

The patient will recover from anaesthesia in the Hybrid Theatre surgical recovery area. The patient will remain at the Hybrid Theatre (for a minimum of 24hrs) until their condition is stable and they are suitable to be transferred back to the UVTHS.
During the post-operative recovery period at the Hybrid Theatre, the patient will be under the care of the LAS anaesthesia team during the day and the UVTHS Veterinary monitoring team overnight. Once the patient has recovered from the anaesthetic and his/her condition is suitable for transfer (awake, drinking fluids and mobile with no neurological signs), they will be transferred back to the UVTHS. We will then arrange for the patient to be discharged back into the owner's care when the Specialist Veterinarian considers this appropriate.

If on day two following the surgery, the patient's condition is considered to be unstable and subsequently not yet suitable for discharge, the Specialist Veterinary Surgeon and the Trial Coordinator will contact the owner to discuss his/her condition. A decision will then need to be made by the patient's owner, with consideration of the Veterinary Specialist's advice, as to whether or not their dog is transported from the Hybrid Theatre back to the UVTHS for ongoing care or whether euthanasia is recommended (if the patient's condition is not improving and the prognosis for recovery is considered to be poor).

NB: Dr Laurencie Brunel will be responsible for discharge of the patient from the UVTHS into the owner's care. In the event that Dr Brunel is unavoidably unable to be present for discharge, the patient will be discharged by a UVTHS Veterinarian with suitable clinical experience and thorough knowledge of the patient's history and of the research trial – as determined by Dr Brunel. The trial coordinator will be present for all admission and discharge appointments.

Patient Transport between the UVTHS and the Hybrid Theatre

The patient will be transferred from the UVTHS to the Hybrid Theatre (approx 200m) and back to the UVTHS post operatively in the UVTHS patient transport vehicle, accompanied by the trial coordinator. They will be transported in either a secure transport cage (for smaller dogs) or a secured car harness (for larger dogs).

The Trial Coordinator will ensure all personnel involved in the post operative care of the patient are fully informed as to the trial procedures. The trial coordinator will remain the first point of contact whilst the patient is hospitalised, however treatment decisions will be at the discretion of the attending specialist and veterinary clinicians. The trial coordinator will be responsible for communication with the owner of the patient. If it is necessary for the patient's owner to speak with specialist veterinary staff, the trial coordinator will arrange for this to happen.

*Refer to attached Hybrid Theatre Flow Chart for further details on Hybrid Theatre procedures/patient care

Post-operative Home Care

The wound should be kept clean and dry, checked daily by the owner and protected from scratching (using an Elizabethan collar if required). This will be sent home with the patient at the time of discharge. There may be some pain associated with eating due to dissection of the muscles of mastication during the surgery.

Soft foods should be given for the first week to minimize possible discomfort associated with this. The patient will be discharged with oral pain relief medication. Antibiotics may also be dispensed at the discretion of the veterinary surgeon. The patient will re-commence oral levetiracetam at home for a total of 7 days post-operatively. The trial coordinator will carry out home-based veterinary checks for three days following the patient's discharge from hospital.

Recovery Period

Dogs will receive only light exercise under veterinary direction during the recovery period (until sutures are removed 10-14 days post-op).

Period of Increased Activity

Owners are again asked to double their dog's baseline activity levels at home for a two week period following implantation surgery. We will measure compliance with the protocol using data collected via the Fitbark device attached to the dog's collar.

Follow-up Assessments

1 month follow-up
Dogs will undertake a veterinary assessment at the ARH. This will include a blood panel (CBC and biochemistry), urinalysis and physical examination to assess their recovery from the procedure. Qualitative feedback from owners will be recorded and the primary care giver will complete the **modified CCDR** (The modified CCDR rating scale compares current behaviour to that prior to treatment rather than comparing to behaviour noted 6 months previously as in the baseline CCDR). They will also complete the CADES at this time.

3 month follow-up

The dog will undertake a **veterinary assessment** at the ARH. This will include a blood panel (CBC and biochemistry), urinalysis and physical examination. A blood sample will be also be collected at this point for the purpose of biobanking for future biomarker analysis. The primary care giver will be asked to complete the **modified CCDR** and CADES and again and qualitative feedback from the owners will be recorded. The dog will be re-tested in the RAM.

6 month follow-up

Dogs will undertake a **veterinary assessment** at the ARH including a blood panel (CBC and biochemistry), urinalysis and physical examination. A blood sample will be also be collected at this point for the purpose of biobanking for future biomarker analysis. The primary care giver will also be asked to complete the **modified CCDR** and CADES and qualitative feedback from the owner will be recorded. The dog will repeat the **RAM**.

Ongoing annual follow-ups
Dogs will have a veterinary assessment (at their regular vet) including blood panel, urinalysis and physical examination. The primary caregiver will also be asked to complete the modified CCDR scale and the CADES and qualitative feedback from the owner will be recorded.

Brain donation at natural death

Following natural death or a decision to euthanize made by the owner, the dog's brain will be removed, fixed and preserved by embedding in paraffin. Neuro-histological analysis of Alzheimer's pathology, SKN cell fate and any anomalous cell growth will then occur at Prof Michael Valenzuelas' PC2 Regenerative Neuroscience Laboratory. Refer to Canine Brain Bank Information Statement and Consent Form attached.

SAFETY AND ADVERSE EVENTS

Definition of serious adverse event potentially related to the study

- Any noticeable post-treatment change in behaviour that appears distressing, uncomfortable or painful to the owner
- Any major Central Nervous System event such as a stroke, encephalitis, seizure or loss of consciousness
- Any neurologic deterioration including significant and acute changes in mobility, vision and or hearing
- Severe and ongoing lethargy or tiredness
- Ongoing loss of appetite or weight loss
- Major ongoing change to sleep pattern
- Major derangement of blood biochemistry or urinalysis
- Unexpected death of animal
- Any new intracranial tumour
- Wound infection non-responsive to therapy in greater than 48hrs.

Definition of adverse events unrelated to study

As the dogs will be old (≥ 8 years), the incidence, progression or deterioration of the following age-related disorders is unlikely to be related to the intervention:

- Arthritis
- Thyroid disease
- Heart disease
- Renal disease or the development of a non-incranial tumour
- Blindness, deafness and chronic comorbid disease present prior to recruitment into the study

Definition of non-serious adverse events not requiring reporting

- Tiredness or lethargy lasting less than 48 hours after surgery
- Change in appetite lasting less than 48 hours after surgery
- Head or jaw soreness lasting less than 48 hours after surgery
- Scratching at the wound site
- Infection of the wound site responsive to antibiotic therapy within 48hrs.

Adverse events and trial decision rules

If at **any stage** during the proposed research a **serious adverse event potentially related to the study** occurs to a participating dog, Prof Michael Valenzuela will contact the Animal Ethics Office and the *Independent Veterinary Safety Review Committee* within 48 hours in order to consider the appropriate course of action.

Actions may include: continuing the study as planned, modification of the protocol, temporarily halting the study until further information is available, or permanently stopping the tria

POWER AND SAMPLE SIZE

Animals treated in this trial are to be combined with our previous trial, and so for statistical purposes overall numbers (N) refers to animals across both trials.

The therapeutic effect size (Cohen's d) has been estimated from those n=3 dogs who have reached the primary 3-month endpoint in our previous trial, assessed by within-subject change on the Canine Cognitive Dysfunction Rating Scale (CCDR). This effect size is Cohen's *d* = 1.5.

Assuming this large effect on CCDR and controlling power at 0.8 and alpha at 0.05, then an overall sample of N=6 is required (computed by G*Power, paired t-test model).

Given our previous trial is providing n=4 dogs with 3-month endpoints, the proposed trial is to recruit a further n= 2 dogs with 3-month endpoints.

STATISTICAL ANALYSIS PLAN

Trial outcomes for continuous variable data are to be assessed by within-group paired t-test following a modified intention-to-treat (ITT) plan. For categorical data, the Chi-square statistic will be used. Modified ITT refers to a pre-specified analysis of all animals who survive at-least 4 weeks post therapeutic cell transplant. For missing data, a LOCF imputation method will be applied (Last Observation Carried Forward). For highly non-normal distributions, transformations will be applied.

<u>Co-Primary Outcomes</u>

1. Safety: refers to general veterinary health check, blood tests and urinalysis at 3-months. An acceptable pre-specified frequency of major adverse events related to study is pre-specified as <= 30% of dogs.
2. Therapeutic efficacy: change from baseline on the CCDR at 3-months.

<u>Secondary Outcomes</u>

1. Safety: as above and expanded to include 1-month, 6-months, 12-months and yearly health checks.
2. Therapeutic efficacy: as above and expanded to include 1-month, 6-months, 12-months and yearly.
3. Therapeutic efficacy: change from baseline in CADES behavioural checklist at 1-month, 3-months, 6-months, 12-months and yearly.
4. Radial Arm Maze: change from baseline at 3-months and 6-months.
5. Function Cloud: change in quantified CCD-related behaviours at 1-month and 3-months.
6. Fitbark: change in quantified activity-related behaviours at 1-month and 3-months.

.

Funding and Competing Interests

This trial is funded by the University of Sydney's Commercialisation Development & Industry Partnerships office, the Yulgilbar Alzheimer Research Program, philanthropic gifts from the Jolly and Smith families and Skin2Neuron Pty Ltd.

Skin2Neuron Pty Ltd is a spin off company of the University of Sydney and holds rights over IP developed by Professor Valenzuela and his group related to this trial. As University of Sydney is a potential shareholder of Skin2Neuron Pty Ltd, then any future profits generated would financially benefit the University of Sydney, Professor Valenzuela and members of his team.

Professor Valenzuela has a conflict of interest management plan in place that governs these relationships and interests that has been approved by the Executive Dean of Faculty of Medicine and Health and the Office of General Counsel.

Pre-treatment CCDR Scale

Canine Cognitive Dysfunction Rating (CCDR) Scale

	Never	Once a month	Once a week	Once a day	> Once a day	Score
How often does your dog pace up and down, walk in circles and/or wander with no direction or purpose?	☐=1	☐=2	☐=3	☐=4	☐=5	=
How often does your dog stare blankly at the walls or floor?	☐=1	☐=2	☐=3	☐=4	☐=5	=
How often does your dog get stuck behind objects and is unable to get around?	☐=1	☐=2	☐=3	☐=4	☐=5	=
How often does your dog fail to recognise familiar people or pets?	☐=1	☐=2	☐=3	☐=4	☐=5	=
How often does your dog walk into walls or doors?	☐=1	☐=2	☐=3	☐=4	☐=5	=
How often does your dog walk away while, or avoid, being patted?	☐=1	☐=2	☐=3	☐=4	☐=5	=
	**Never**	**1–30% of the time**	**31–60% of the time**	**61–99% of the time**	**Always**	
How often does your dog have difficulty finding food dropped on the floor?	☐=1	☐=2	☐=3	☐=4	☐=5	=
	**Much less**	**Slightly less**	**The same**	**Slightly more**	**Much More**	
Compared to six months ago, does your dog now pace up and down, walk in circles and/or wander with no direction or purpose?	☐=1	☐=2	☐=3	☐=4	☐=5	=
Compared to six months ago, does your dog now stare blankly at the walls or floor?	☐=1	☐=2	☐=3	☐=4	☐=5	=
Compared to six months ago, does your dog urinate or defecate in an area it has previously kept clean (if your dog has never house-soiled, tick 'the same')?	☐=1	☐=2	☐=3	☐=4	☐=5	=
Compared to six months ago, does your dog have difficulty finding food dropped on the floor?	☐=1	☐=2	☐=3	☐=4	☐=5	Multiply by 2 =
Compared to six months ago, does your dog fail to recognise familiar people or pets?	☐=1	☐=2	☐=3	☐=4	☐=5	Multiply by 3 =
	**Much more**	**Slightly more**	**The same**	**Slightly less**	**Much less**	
Compared to six months ago, is the amount of time your dog spends active?	☐=1	☐=2	☐=3	☐=4	☐=5	=

Dr Kaylene Jones
Trial Coordinator
kaylene.jones@sydney.edu.au

Regenerative Neuroscience Group
Brain and Mind Centre
University of Sydney
94 Mallett Street,
Camperdown, NSW 2050

Your name:

Email:

Pet name:

Age:

Sex:

Breed:

Post-treatment CCDR Scale

Canine Cognitive Dysfunction Rating (CCDR) Scale

	Never	Once a month	Once a week	Once a day	> Once a day	Score
How often does your dog pace up and down, walk in circles and/or wander with no direction or purpose?	☐=1	☐=2	☐=3	☐=4	☐=5	=
How often does your dog stare blankly at the walls or floor?	☐=1	☐=2	☐=3	☐=4	☐=5	=
How often does your dog get stuck behind objects and is unable to get around?	☐=1	☐=2	☐=3	☐=4	☐=5	=
How often does your dog fail to recognise familiar people or pets?	☐=1	☐=2	☐=3	☐=4	☐=5	=
How often does your dog walk into walls or doors?	☐=1	☐=2	☐=3	☐=4	☐=5	=
How often does your dog walk away while, or avoid, being patted?	☐=1	☐=2	☐=3	☐=4	☐=5	=
	**Never**	**1–30% of the time**	**31–60% of the time**	**61–99% of the time**	**Always**	
How often does your dog have difficulty finding food dropped on the floor?	☐=1	☐=2	☐=3	☐=4	☐=5	=
	**Much less**	**Slightly less**	**The same**	**Slightly more**	**Much More**	
Compared to before your dog's treatment, does your dog now pace up and down, walk in circles and/or wander with no direction or purpose?	☐=1	☐=2	☐=3	☐=4	☐=5	=
Compared to before your dog's treatment, does your dog now stare blankly at the walls or floor?	☐=1	☐=2	☐=3	☐=4	☐=5	=
Compared to before your dog's treatment, does your dog urinate or defecate in an area it has previously kept clean (if your dog has never house-soiled, tick 'the same')?	☐=1	☐=2	☐=3	☐=4	☐=5	=
Compared to before your dog's treatment, does your dog have difficulty finding food dropped on the floor?	☐=1	☐=2	☐=3	☐=4	☐=5	Multiply by 2 =
Compared to before your dog's treatment, does your dog fail to recognise familiar people or pets?	☐=1	☐=2	☐=3	☐=4	☐=5	Multiply by 3 =
	**Much more**	**Slightly more**	**The same**	**Slightly less**	**Much less**	
Compared to before your dog's treatment, is the amount of time your dog spends active?	☐=1	☐=2	☐=3	☐=4	☐=5	=

Dr Kaylene Jones
Trial Coordinator
kaylene.jones@sydney.edu.au

Regenerative Neuroscience Group
Brain and Mind Centre
University of Sydney
94 Mallett Street,
Camperdown, NSW 2050

Your name:

Email:

Pet name:

Age:

Sex:

Breed:

REGENERATIVE NEUROSCIENCE GROUP

THE UNIVERSITY OF SYDNEY

CADES Questionnaire

The CADES scale A. Madari et al. / Applied Animal Behaviour Science 171 (2015) 138–145

Canine Dementia Scale

	Points Scored	Totals
**A. SPATIAL ORIENTATION**		
1. Disorientation in a familiar environement (inside/outside)		
2. Failure to recognise familiar people and animals inside or outside the house/apartment		
3. abnormally respond to familiar objects (a chair, a wastebasket)		
4. aimless wandering (motorically restless during the day)		
5. a reduced ability to do a previously learned task.		
(score 0-25)		
**B. SOCIAL INTERACTION**		
6.Changes in interaction with a man/dog, dog/other dog (playing/petting/welcoming)		
7. changes in individual dog behaviour Iexploration behaviour, play, performance)		
8. response to commands and ability to learn new task		
9. irritable		
10. expression of aggression		
(score 0-25)		
**C. SLEEP-WAKE CYCLES**		
11. abnormally responds in night (wandering, vocalization, motorically restless		multipy x 2
12. switch over from insomnia to hypersomnia		multiply x2
(score x 2, 0-20)		
**D. HOUSE SOILING**		
13.eliminate at home at random locations		
14.eliminate in its kennel or sleeping area		
15. changes in signalisation for elimination activity		
16. eliminate indoors after a recent walk outside		
17 eliminate at uncommon locations (grass, concrete)		
(score 0-25)		
Total score (A+B+C+D) 0-95		0

Clinical stage	Score
Normal ageing	0-7
Mild cognitive impairment (8-23)	8- 23
Moderate cognitive impairment (24-44)	24-44
Severe cognitive impairment (45-95)	45-95

SCORING SYSTEM

0 points	Abnormal behaviour never observed
2points	abnormal behaviour of the dog was detected at least once in the last 6 months
3 points	abnormal behaviour appeared at least once per month
4 points	abnormal behaviour was seen 2 to 4 times per months
5 points	abnormal behaviour was observed several times a week

DOG LIFESTYLE AND BEHAVIOUR QUESTIONNAIRE (Pre-trial screening)

Interviewer Date:
Owner's Name:
Dog's Name Age Breed:
Sex If neutered, at what age?
Been to puppy preschool?
Description of the problem behaviours:

VETERINARY HISTORY

Medical history
 Is dog on any medication now for any medical problem?
 Is dog on any medication now for any behavioural problem?
 Has dog been on any medication in the past for any medical problem?
 Has dog been on any medication in the past for any behavioural problem?

Surgical history
Any surgery other than desexing?

HOUSEHOLD

Humans in household (including names and age)
Other animals in household (including names, species, age)
In what sequence were the animals in household acquired?
What is dog's relationship to the other animals in household?
Any major changes in household (humans and animals) since acquiring the dog?

DWELLING

What type of area do owners live in: CITY SUBURB RURAL
Type of home:
Apartment with ………bedroom(s)
House with ………….. bedroom(s)
Other……………………………….
Any major changes (including house moves) since acquiring the dog?

ACQUISITION AND BACKGROUND

At what age was the dog aquired?
What do you know about the animal's parents' behaviour?
Where?
Any history prior to acquisition?

'We would like to record what we can about your animal's early experience and to simplify matters we will ask about it in two parts: before and after four months of age'.

Describe early exposure (<4 months of age) to:
Various novel stimuli
Children
Dogs
Other species

Describe juvenile exposure (> 4 months of age) to:
Various novel stimuli
Children
Dogs
Other species

‘We would like to record what we can about your animal’s problem behaviour and to simplify matters we will ask about it in four parts: when it first occurred, how it has developed since then, what corrective approaches you have tried and how the behaviour appeared the last time it occurred’.

First episode of problem behaviour:
Describe animal’s behaviour
At what age?
Where?
Times of day?
Associated factors
Owner’s response

Development of problem behaviour:
Describe animal’s behaviour
At what age?
How often?
Where?
Times of day?
Associated factors
Owner’s response

Attempted modification:
Types (we need to know the whole list)
Describe animal’s behaviour (we need to know the animal’s responses for the whole list)
Owner’s response
Current status of problem behaviour:
Describe animal’s behaviour
Associated factors
Owner’s response

‘Now we are going on to ask some questions about your animal’s lifestyle and behaviour in general, and how they have changed over time’.

Feeding:
Types
Before or after humans?

Where?
Which person(s)?
Any teeth baring/growling/snapping/biting?
Times of day?
How often?
Giving titbits?
If yes, describe animal's behaviour
Types
How often?
Times of day?
Where?
Which person(s)?
Any teeth baring/growling/snapping/biting?
Giving bones?
If yes, describe animal's behaviour
Types
How often?
Times of day?
Where?
Which person(s)?
Any teeth baring/growling/snapping/biting?
Have any of the above behaviours changed over time? When did the change occur and what was the nature of the change?

Favourite games:
Types
Does your dog obey in 90% of circumstances when asked to retrieve and give toys?
Times of day?
Where?
How often?
Which person(s)?
Any teeth baring/growling/snapping/biting?
Describe any rough games you have with your dog
Have any of the above behaviours changed over time? When did the change occur and what was the nature of the change?

Exercise:
How often?
Types
Hours per week off-lead off your property?
Times of day?
Where?
Which person(s)?
Have any of the above behaviours changed over time? When did the change occur and what was the nature of the change?

Control:
When easiest?
Describe your use of any of the following in dog control:
physical contact / choke chains / food rewards / toys?
When most difficult?
When does dog pull on lead, if at all?
Does your dog comply in 90% of circumstances when asked to lie down on command?
Name the other commands your dog obeys 90% of the time
....
....
Any teeth baring/growling/snapping/biting?
Have any of the above behaviours changed over time? When did the change occur and what was the nature of the change?

Grooming:
Types.
Times of day?
Where?
How often?
Which person(s) ?
Any teeth baring/growling/ snapping/biting?
Have any of the above behaviours changed over time? When did the change occur and what was the nature of the change?

Bathing:
Types.
Times of day?
Where?
How often?
Which person(s) ?
Any teeth baring / growling / snapping /biting?
Have any of the above behaviours changed over time? When did the change occur and what was the nature of the change?

Animal's sleeping areas:
Where?
If your dog gets on to beds/furniture, describe animal's behaviour when told get off
....
....
Any associated teeth baring / growling / snapping / biting?
Have any of the above behaviours changed over time? When did the change occur and what was the nature of the change?

Attention-seeking behaviours:
Types
How often?
Times of day?
Where?
Which person(s)?
Any teeth baring/growling/snapping/biting?
Has this behavior changed over time? When did the change occur and what was the nature of the change?

Describe animal's response to members of the household (adults and children):
Leaving home
Arriving at home

Describe animal's response to other adults (familiar):
Arriving at home
Leaving home

Describe animal's reaction to seeing unfamiliar adults:
Arriving at home
Leaving home
In public

Describe animal's reaction to seeing unfamiliar children:
Arriving at home
Leaving home
In public

Describe animal's reaction to seeing other dogs:
In public.
From inside car and from inside yard.
When other dogs are around, does your dog return to you on command while off your property?

Describe animal's reaction to seeing cats:
In public.

Describe animal's reaction to seeing moving cars/joggers/people on pushbikes/ roller blades/skateboards:
In public.
Anything else?
Describe animal's behaviour.
How often?
Times of day?
Have any of the above behaviours changed over time? When did the change occur and what was the nature of the change?

QUESTIONS SPECIFIC TO THE DOGS+CELLS TRIAL:

Which signs of CCD, listed on the CCDR are most distressing to you, or affect your bond with your dog the most?
Do you consider any of the signs to be currently untenable?
In what order did the signs appear, and at what time?
Having discussed the trial protocol and proof-of-concept, in an ideal world, what are your expectations for your dog as a result of participating?
Realistically, what are your expectations?
What changes would be minimally relevant to you (what is the minimal change that you would consider to be a successful outcome)?

How would you prefer you dog to be identified during and after his/her participation in the trial, to those working with him/her and in any publications or presentations? (ie. 'Deefa', 'Deefa D.', 'FemaleNBoxer', Dog #001)

Veterinary 'Fitness for Trial' Checklist

Owner's name:______________________________________

Dog's name:____

Dog's age:____

Medical history

Does this dog have any diseases/illnesses that may preclude it from inclusion in this study?

No ☐ Yes ☐ Details:________________________________

Has this dog previously had any adverse reactions to anesthetics or surgery?

No ☐ Yes ☐ Details:________________________________

Does this dog have any diseases/illnesses that may increase the risk of a surgical procedure?

No ☐ Yes ☐ Details:________________________________

Current medical status

Does this dog have an abnormal T4 level?

No ☐ Yes ☐ Details:________________________________

Does this dog have any other abnormal blood results?

No ☐ Yes ☐ Details:________________________________

Does this dog have any abnormal urinalysis results?

No ☐ Yes ☐ Details:________________________________

If yes to any of the above, are these results indicative of any disease/illness that may cause cognitive impairment of CCD-like symptoms?

NA ☐ No ☐ Yes ☐ Details:________________________________

Please rate this dog's level of eyesight:

Very bad ☐ Bad ☐ Borderline ☐ Reasonably good ☐ Normal ☐

Please rate this dog's degree of movement/ability to get around:

Very bad ☐ Bad ☐ Borderline ☐ Reasonably good ☐ Normal ☐

Using the body condition score chart provided, what body condition score is this dog?

1. Very thin ☐ 2. Thin ☐ 3. Ideal ☐ 4. Overweight ☐ 5. Obese ☐

Suitability for inclusion in this study:

Do you believe this dog is physically fit enough to undertake anesthesia and brain surgery?

No ☐ Yes ☐ Details:____

____

____

Body Condition Scoring Chart

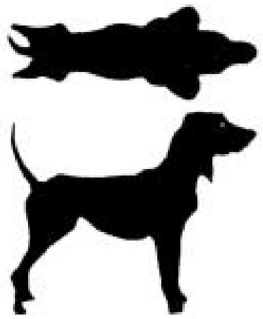

1 Very Thin

Ribs	Easily felt with no fat cover
Tail base	Bones are raised with no tissue between the skin and bone
Side view	Severe abdominal tuck
Overhead	Accentuated hourglass shape

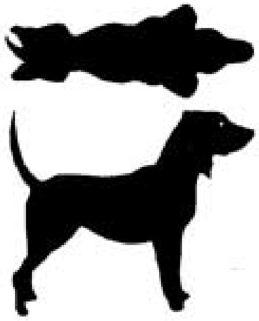

2 Thin

Ribs	Easily felt with no fat cover
Tail base	Bones are raised with minimal tissue between the skin and bone
Side view	Abdominal tuck
Overhead	Marked hourglass shape

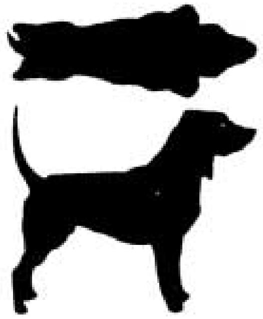

3 Ideal

Ribs	Easily felt with slight fat cover
Tail base	Smooth contour but bones can be felt under a thin layer of fat
Side view	Abdominal tuck
Overhead	Well proportioned lumbar waist

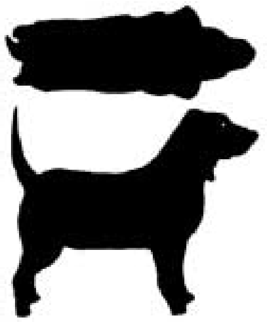

4 Overweight

Ribs	Difficult to feel with moderate fat cover
Tail base	Some thickening but bones can be felt under a moderate layer of fat
Side view	No abdominal tuck or waist
Overhead	Back is slightly broadened

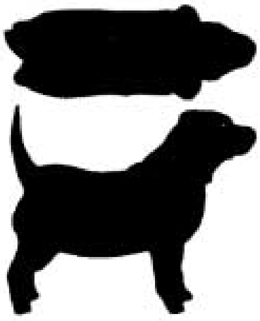

5 Obese

Ribs	Difficult to feel under thick fat cover
Tail base	Thickened and difficult to feel under a prominent layer of fat
Side view	Fat hangs from the abdomen and there is no waist
Overhead	Markedly broadened

Radial Arm Maze

Location – Ecological Research Centre, The University of Wollongong

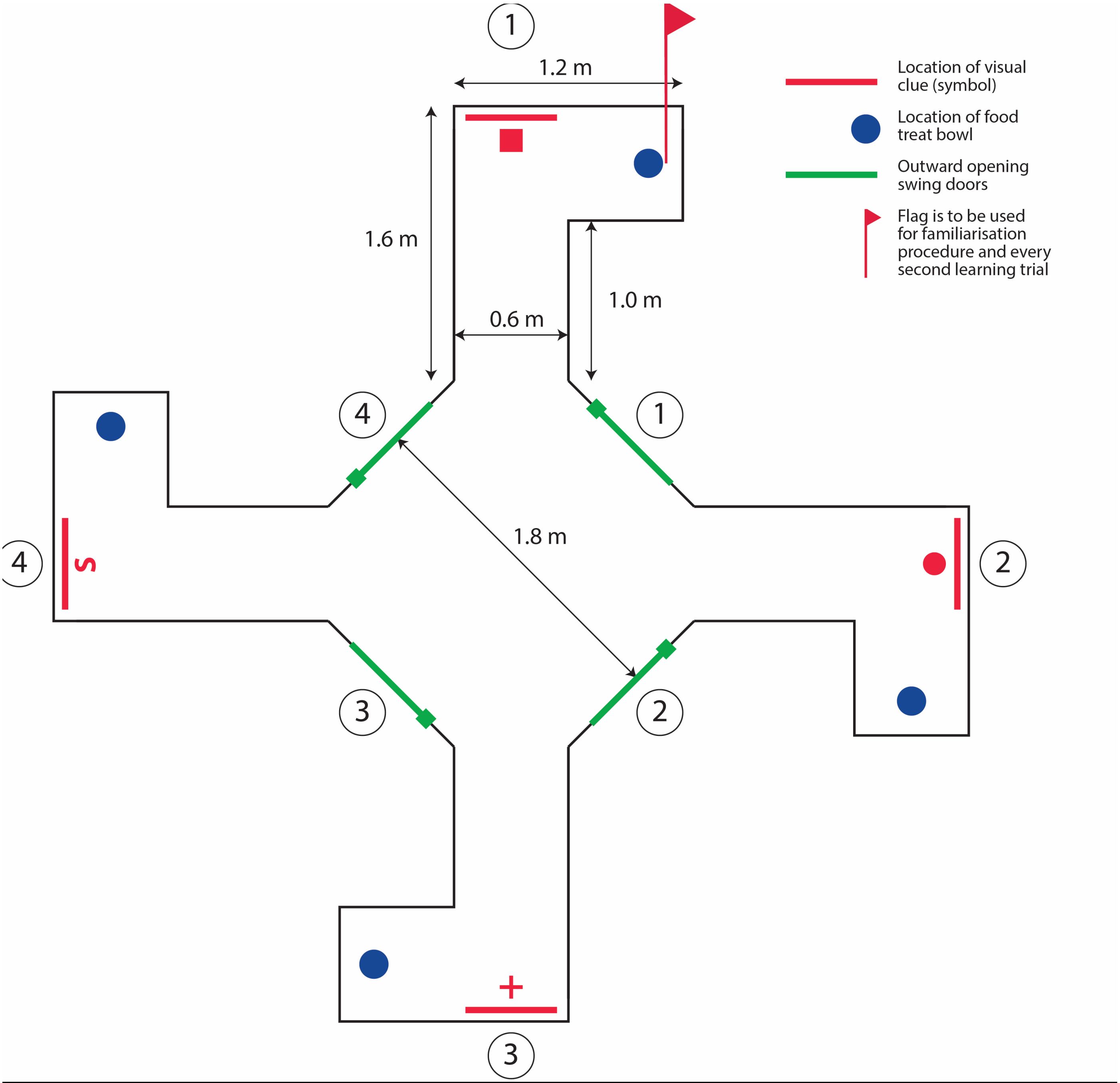

Radial Arm Maze

Radial Arm Maze Testing Procedure

- The maze is damp mopped thoroughly with an odour neutraliser - ensuring that the surface is free of any debris and that the signs are clearly visible.

- Plastic food bowls are placed at the end of each arm. Each bowl consists of 2 layers - the bottom layer containing 3 food treats. The top layer of the bowl is perforated to allow the odour from the underlying treats to permeate each bowl.

  This will serve as an odour cue control.

- Place 1 x liver treat in the bowl at the end of arm 1. This bowl will be marked by a flag on a long pole such that it is visible to the dog from all points within the maze.

Acclimatisation

The dog enters the arena and is allowed to freely explore the maze for 5 minutes in the absence of any (available) treat or flag.

Familiarisation Procedure

1. Dog enters the maze 4 times, once through each door. The order of the door entry will be a) door 1 b) door 3 c) door 2 d) door 4
2. The dog is allowed 90 seconds each time to find the reward which is marked by a flag at the end of arm 1.
3. The dog is allowed to eat one reward and is then removed from the maze, preferably by calling the dog out through the nearest door.
4. If the dog fails to find the reward, it is shown the location, allowed to eat the treat, then removed.

5. The maze should be damp mopped between each familiarisation session and the treats replaced if needed.
6. If the dog fails to learn the location of the flagged reward by the 4th trial it is eliminated from further testing.

Learning Trial Procedures

- There are 8 learning trials
- The flag is used for every 2nd learning trial i.e. L1,L3,L5 and L7
- There is no flag at the reward location for L2,L4,L6 and L8.

1. The dog enters the maze through the doors such that it enters the maze twice through each door but the pattern of entry is randomised - the randomisation using random.org has resulted in a door entry sequence of i) door 3, ii) door 2, iii) door 3, iv) door 4, v) door 1, vi) door 1, vii) door 4, viii) door 2.

   The same sequence can be used for each dog as they are only in the maze for the one series of tests, however if for any reason the dog is retested - the door sequence, reward and probe locations must be changed.

2. After each trial, the reward should be replaced and the maze mopped to eliminate any scent trail from the dog or the investigator.
3. The dog should be removed from the maze after it has eaten the reward.
4. If the dog fails to locate the reward within the 90 seconds it should be shown the position of the reward by calling the dog to the reward area and allowing the dog to eat the reward.
5. At the completion of the 8 learning trials, the dog is removed from the maze, given water, taken to the toilet and allowed to rest for a 90 minute period. This is the retention period.

Probe Trial

- In this trial, a 'dummy' reward is placed in the bowl at the end of arm 4. This is a 1/4 turn around the maze. The purpose of this is to ensure that the dog has not been sniffing out the treats.
- No rewards are placed at the learned reward zone (arm 1) for the probe trial.
- The maze is mopped after the rewards are placed to ensure that the investigator does not leave a scent trail to the rewards.
- The dog enters the maze once only. The dog should enter through door no. 2, such that it is an equal distance away from both the learned reward location and the dummy/probe location.

- The dog is allowed 90 seconds in the maze - it is then given a reward and removed from the maze.
- The video footage is then used to measure the time taken to reach the previously learned annulus.

SKN culture: Cell Culture Protocols

Isolation protocol (Modified from Valenzuela, 2008)

Upon arrival at the Regenerative neuroscience laboratory, tissue is washed with Antibiotic-Antimycotic in DPBS, chopped into 1-2mm pieces, and incubated in 15ml of 0.1% Trypsin at 37C /5% CO_2 for 40 minutes. At the end of the incubation period, solution is aspirated and 5mL of 0.1% DNAse 1 added for 1 minute at room temperature. Following a final aspiration, tissue is transferred to a glass petri dish and mechanically chopped until the pieces form a paste-like consistency. Suspension is placed on mesh strainer over a collection vessel and tissue fragments macerated against the mesh with a glass pestle. The cell suspension collected is then poured through a 40um cell strainer, centrifuged at 350 x g for 10 minutes, resuspend in fresh DMEM/F12 3:1 supplemented with 1% Antibiotic-Antimycotic, 2% B27 nutrient mix, and growth factors egf and fgf2 at 20ng/mL and 40ng/mL respectively, hereafter referred to as complete media. A cell count is performed using a haemocytometer and $1x10^6$ cells are added per well of a 6 well plate in a total volume of 5mL of complete medium per well. Incubating at 37C / 5% CO_2, neurospheres should start to form within 3-4 days. Culture is ready to be transferred to adherent culture when the majority of spheres are >50um in diameter.

Transferring to adherent culture

Coating of culture vessels with 0.1% Gelatin should occur at least 1 hour prior starting the transfer procedure. Collect sphere suspension via centrifugation. Discard the supernatant and add 1mL of pre-warmed TryPLE select. Incubate at 37C /5% CO_2 for 5 minutes, followed by manual trituration 200 times. Neutralise TryPLE using DMEM/F12 3:1, centrifuge at 350 x g for 10 minutes, count and seed cells at a density of 500 000 cells / T25 flask. Complete media change occurs every 3 days.

Passaging/ Continual culture

Flasks are ready for passage when 80% confluence is achieved. Coating of culture vessels with 0.1% Gelatin should occur at least 1 hour prior to passage time. Discard spent medium from the cell culture flask and add 2mL of TryPLE. Incubate for 2 minutes at 37C/5%CO_2, gently tap sides of flask to dislodge cells, check under microscope to ensure majority of cells are detached. Neutralise TryPLE using DMEM/F12 3:1, centrifuge at 350 x g for 10 minutes, discard supernatant, resuspend, count and seed cells in complete media at a density of 250 000 cells / T25 flask.

EdU labelling of cells

Cells will be treated with EdU, a thymidine analog that is incorporated into the DNA of dividing cells. Later, in post mortem analysis of brain tissue, this EdU molecule can then be fluorescently labeled in order to track the transplanted cells.

Thymidine analogs have been used extensively for the analysis of cellular DNA synthesis, tracking of transplanted cells, and identification of tissue resident stem cells (Lin et al 2009; Santos-Ferreira et al 2016; Ning et al 2013). BrdU (a similar analog to EdU) has been used for several decades in over 20,000 studies where it has been shown to be both safe and effective in its cell labelling capacity (Cavanagh et al 2011). Cell labelling of EdU in culture can be easily performed using the Click-It® reaction (Invitrogen). In addition, the detection of EdU labelled cells requires no harsh treatment or immunological reaction as is the case with detection of BrdU labelled cells. (Lin et al 2009).

Cells will be treated with EdU in the adherent cell culture phase, following a standard published protocol (Lin et al 2009). Briefly, this entails exposure of the cells to EdU for 48-72 hours. The cells are then cultured for a further 3 days in fresh media, followed by 5 washes to ensure that there is no residual EdU remaining in the media prior to harvesting for transplantation.

Loading into tube for transplant

As per passaging protocol above. Following centrifugation, resuspend in 500ul of DMEM, perform a count and add 500 000 cells to a PCR tube. Keep a small aliquot aside for viability and EdU expression testing. Spin the PCR tube for 5 minutes at 350 x g and resuspend in 15ul of PBS, transport immediately to transplantation location.

SKN culture: Timeline

Canine SKN EdU proliferation experiments conducted in 2009-2010 show decreased proliferation rates following the third passage (Figure 1). Passage data from 12 canine SKN isolations indicated an average time of approximately 22 days to reach the second passage (Figure 2). The optimal time for transplant should be post isolation day 28. This will maximise cell numbers and allow for appropriate quality control checks to occur.

Figure 1:

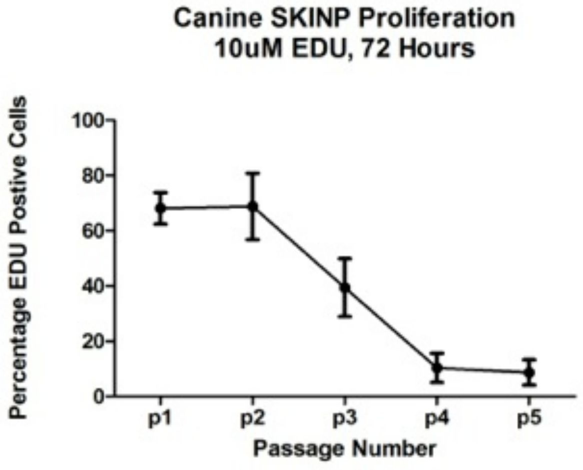

Figure 2:

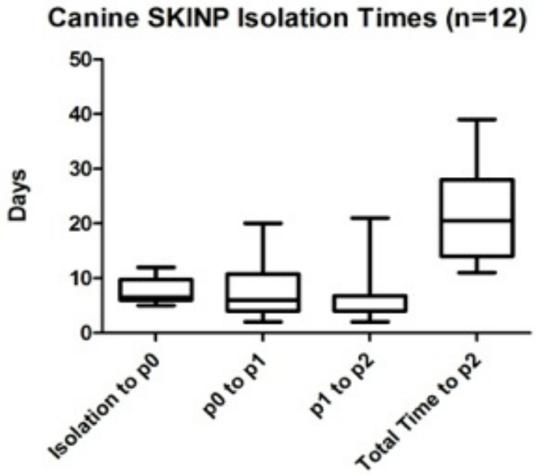

	Day 7	Day 14	Day 21	Day 28
	**P0**	**P1**	**P2**	**P3 / Transplant**
**Predicted Cell No**	-	1.25×10^6	2×10^6	1.5×10^6
**No of T25 Flasks**	2	4 (1×10^6)	3 (7.5×10^5)	
**Cryopreservation**		1 vial (2.5×10^5)	1 vial (2.5×10^5)	
**Staining**			1×10^5	
**EDU Assay**			6×10^4	
**Trypan Blue Assay**				2×10^3

SKN culture: Quality Control Criteria / Summary Checklist

**Criterion**		
**Stage 1: Isolation**	**Yes**	**No**
Timing: Time between surgery and start SKN isolation < 2hrs?		
Size: Surface area (length x width) > 2 cm^2?		
**Stage 2: Neurosphere Culture (Day 5 Check)**	**Yes**	**No**
Contamination: All seeded wells contaminant free since isolation?		
Sphere Size 50-100um?		
**Stage 3: Adherent Culture (p0 pre-passage 1 check)**	**Yes**	**No**
Contamination: All seeded flasks contaminant free since isolation Day 5 Check ?		
Adherent culture >80% confluent?		
Absence of fibroblastic cells?		
Cell count < 500 000 / flask		
Time from P0 to P1 greater than 10 days?		
**Stage 4: Quantitative Gene Expression Profile (Following Passage 2) (Figure3)**	**Yes**	**No**
Relative expression of p75 gene greater than fibroblast control?		
**Stage 6: Protein Expression Immunocytochemistry (Following Passage 2) (Figure 4)**	**Yes**	**No**
Percentage of Nestin positive cells > 70%?		
Percentage of βIII-tubulin positive cells > 80%?		
**Stage 7: EDU Assay (Following Passage 2)**	**Yes**	**No**
Percentage EDU positive cells greater than 20%?		
**Stage 8: Trypan Blue Exclusion Assay (Transplant Day)**	**Yes**	**No**
Percentage of Live cells >85%?		

SKN culture: Quality Control Protocols

Trypan Blue Staining

In an eppendorf tube add 30ul of cell suspension to 30ul of Trypan blue dye. Using a haemocytometer count first the number of dead cells (blue stain) followed by the total number of cells. Record and calculate percentage viability. Ensure count is performed immediately following addition of Trypan blue.

EDU Staining

Adherent cultures are treated with 10um 5-ethynyl-2'-deoxyuridine (EDU, Invitrogen) for 72 hours. Cells are fixed with 4% paraformaldehyde and visualised according to manufacturer's instructions. Counterstaining is performed with 1:5000 Hoecsht 33342 (Invitrogen) and coverslipped. Quantification is performed using CellProfiler cell analysis software.

Immunocytochemical Analysis (Adherent Cultures)

Cells for analysis are fixed with 4% paraformaldehyde (Sigma-Aldrich) in 0.1% PBS at room temperature for 15 minutes, washed 3 times with PBS and incubated with 0.1% Triton-X for permeabilisation. Cells are then incubated for 1 hour at room temperature in blocking solution containing 3% Donkey serum / 1% BSA in 0.1% PBS. For primary antibody incubation blocking solution with either <insert antibodies and concentrations> were incubated overnight at 4C. Cells are washed with PBS three times, followed by incubation in blocking solution containing a 1:500 dilution of Alexofluor 488 or 594. Cells are counterstained with DAPI mounting medium and coverslipped. Images analysis occurs via image capture using fluorescence microscope and quantification using Cellprofiler image analysis softwar

Therapeutic cell dose, target and delivery

Delivery of 200,000-250,000 autologous SKN cells suspended in 20 microL carrier media of phosphate buffered saline by fine needle injection into each bilateral dorsal hippocampi using MR-CT guidance (i.e, ~500,000 cells in total; surplus cells will be characterized *in vitro*).

Syringe priming procedure for cell transplantation

SKN cells will be suspended in PBS. This procedure is designed to prime the syringe with vehicle fluid so that dead space in the needle is filled, hence allowing efficient drawing up of cell suspension without air bubbles.

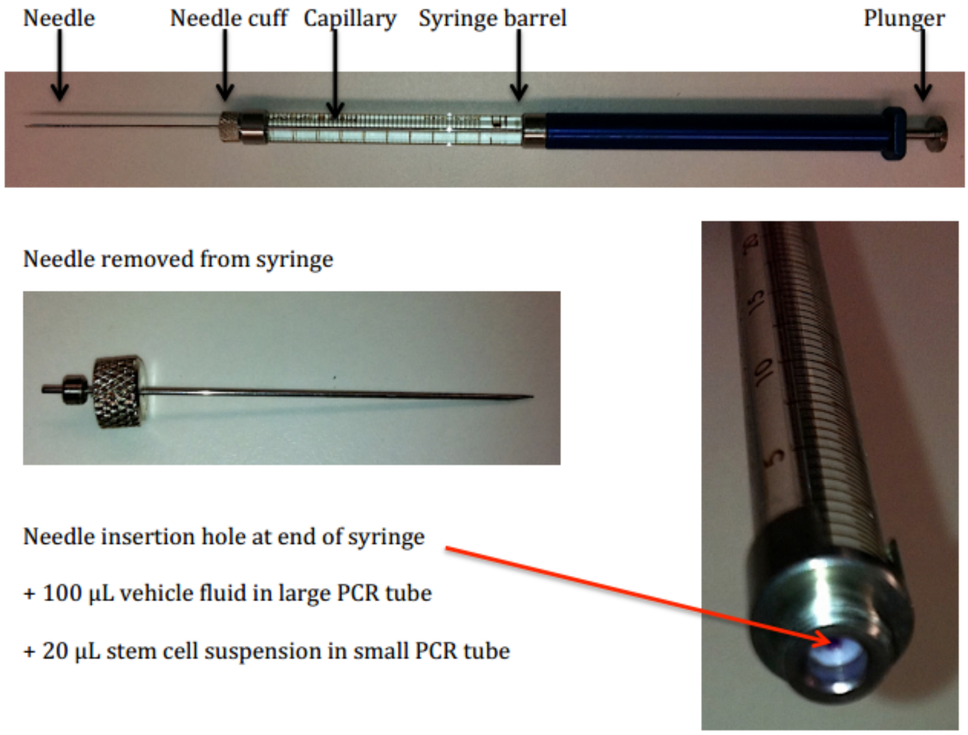

With needle fitted to syringe, draw up 25 µL vehicle fluid. Expel approximately 20 µL fluid, ensuring that syringe stopper is clear visible inside capillary. Undo needle cuff and remove needle from syringe. Needle is now filled with vehicle fluid. Expel all residual fluid from syringe by pushing plunger down to end point. Carefully re-insert back end of needle into insertion hole at end of syringe and tighten needle cuff. Can now draw up stem cell suspension directly without any dead space.

Management of seizures - DOGS+CELLS Trial – Dr Georgina Child, May 2019

Pre-treatment with levetiracetam 20mg/kg q8 hours PO. Start treatment 24 hours (or more) prior to procedure. There is sedation associated with this drug compared with other anticonvulsants in my experience and has a rapid onset of action. It can cause ataxia and sedation. It is renally excreted so dose should be at lower end for dogs with renal insufficiency. Toxicity has not been reported with this drug in dogs to my knowledge. Post operatively I would continue the drug for at 1 week. There should not be a problem discontinuing abruptly.

On the day of the cell transplant surgery – injectable dose of levetiracetam to be given sc 20mg/kg (diluted 1:2 with sterile saline) q8 hours. Once oral medication can be given postoperatively, resume administration orally. Levetiracetam can be given at the same time as the premed the morning of the procedure. Premed selection and anaesthetic induction agent up to the attending anaesthetist/veterinary specialist.

- All dogs are to have a patent IV catheter post operatively until discharge

- If seizures are seen post operatively.

1/ Diazepam should be given slowly IV only if a seizure continues for more than 90 secs. Dose 0.5- 1mg/kg. Max 20mg per dog. As these dogs are old I would give initial dose of 0.5mg/kg.

Midazolam may be given instead of diazepam. It isn't a better anticonvulsant but can be give SC if needed.

Diazepam has a peak concentration 2 mins after IV injection. Note Diazepam can cause hyperexcitability/agitation. No more than 3 doses of Diazepam should be used in 24 hours. I would not recommend using more than 2 doses in older dogs. Midazolam (0.05-0.3mg/kg) IV or IM can be used instead but is longer acting.

The depressive cardiac and respiratory effects of benzodiazepines increase with repeated doses and are much longer lasting than anticonvulsant effects. In my experience larger cumulative doses of benzodiazepines are also more likely to be associated with difficulties swallowing and risk of aspiration. Tolerance to the anticonvulsant effects of benzodiazepines also develops relatively quickly

2/ Also give Levetiracetam (40 -60mg/kg) after diazepam. It does not need to be diluted. This drug can be given slowly IV or can be given SC if dog stops seizuring after 1 dose of diazepam IV. Use with diazepam may potentiate the anticonvulsant effects of both. Diazepam has a short duration of action and Levetiracetam has a longer duration. Continue treatment at 20mg/kg every 8 hours (IV, subcutaneously or orally) for at least 48 hours after seizures have stopped. For any dog with renal insufficiency I would use 10mg/kg q8 hours for at least 48 hours.

For dogs where seizure stops without treatment give levetiracetam only - IV, IM or SC at a dose 30mg- 40 /kg and continue 20mg/kg dose orally for at least 48 hours.

3/ If seizures are not controlled with 2 doses of diazepam and levetiracetam then the dog should be given propofol to effect (bolus 0.5-2mg/kg). CRI propofol 0.02-0.5mg/kg/ minute (max 5mg/kg/hour) should be started if ongoing seizure activity is seen. Dog should be intubated if unable to swallow. Oxygen should be provided via ET tube or nasal tube, blood pressure monitored and IV fluids started at maintenance rate if not already provided.

Levetiracetam should be continued q8 hours. Propofol infusion should be stopped every 30mins and recommenced if ongoing seizure activity.

If treatment with propofol is required start phenobarbitone 5mg/kg IV q 6-12 hours (maximum 20mg/kg/24 hours). It will take at least 30mins for phenobarb to have an effect and some time to reach a steady serum level. When seizure free for 24 hours reduce dose of phenobarbitone to 3-4mg/kg q12 hours.

Treatment with anticonvulsants (levetiracetam and or phenobarbitone) after 48 hours should determined on a case by case basis if indicated.

References

Blurton-Jones, M., M. Kitazawa, et al. (2009). "Neural stem cells improve cognition via BDNF in a transgenic model of Alzheimer disease." Proc Natl Acad Sci U S A **106**(32): 13594-13599.

Brookmeyer, R., E. Johnson, et al. (2007). "Forecasting the global burden of Alzheimer's disease." Alzheimers Dement **3**(3): 186-191.

Cavanagh, B.L., Walker, T, et al. (2011). "Thymidine analogues for tracking DNA synthesos." Molecules 16(9): 7980-7993.

Duncan T, Valenzuela M (2017) Alzheimer’s disease, dementia and stem cell therapy. Stem cell research and therapy. 2017;8:111

Duncan T, Lowe A, Sidhu K, Lin R, Sytnyk V, **Valenzuela M** (2017) Replicable Expansion and Differentiationof Neural Precursors from Adult Canine Skin. Stem Cell Reports ISSCR 9(2):557-570

Head, E., R. McCleary, et al. (2000). "Region-specific age at onset of β-amyloid in dogs." Neurobiology of Aging **21**(89): 96.

Hughes, C., L. Berg, et al. (1982). "A New Clinical Scale for the Staging of Dementia." British Journal of Psychiatry **140**: 566-572.

Jorm, A., A. Korten, et al. (1987). "The prevalence of dementia: a quantitative integration of the literature." Acta Psychiatrica Scandinavica **76**: 465-479.

Lewczuk, P., Ermen, N. et al (2018) "Plasma neurofilament light as a potential biomarker of neurodegeneration in Alzheimer's disease" Alzheimer's Research and Therapy 10:71

Lin , G., Huang, Y, et al. (2009). "Labeling and Tracking of Mesenchymal Stem Cells with EdU." Cytotherapy 11(7): 864-873

Ning, H., Albersen, M. et al. (2013). "Effects of EdU Labeling on Mesenchymal Stem Cells." Cytotherapy 15(1):57-63.

Palop, J. J. and L. Mucke (2010). "Amyloid-beta-induced neuronal dysfunction in Alzheimer's disease: from synapses toward neural networks." Nat Neurosci **13**(7): 812-818.

Qu, T., H. Brannen, et al. (2001). "Human neural stem cells improve cognitive functions of aged brain." Neuroreport **12(6)**: 1127-1132.

Roberts, T., P. **McGreevy**, M **Valenzuela**. (2010). "Human Induced Rotation and Reorganization of the Brain of Domestic Dogs." PLoS One **5**(7): e11946

Roy, N., C. Cleren, et al. (2006). "Functional engraftment of human ES cell-derived dopaminergic neurons enriched by coculture with teleromase-immortalised midbrain astrocytes." Nature Medicine **12**: 1259-1268.

Santos-Ferreira, T., Llonch, S, et al. "Retinal transplantation of photoreceptors results in donor-host cytoplasmic exchange." Nature Communications DOI:10.1038/ncomms13028

Salvin, H., P. **McGreevy**, et al. (2010). "Underdiagnosis of Canine Cognitive Dysfunction: A cross-sectional survey of older companion dogs." The Veterinary Journal **184**:277-281.

Salvin, H., P. **McGreevy**, et al. (2011). "The Canine Sand Maze (CSM): An appetitive spatial memory paradigm sensitive to age-related change in companion dogs." Journal of the Experimental Analysis of Behaviour. **95:**109-118

Salvin, H. E., P. D. **McGreevy**, et al. (2010). "The canine cognitive dysfunction rating scale (CCDR): A data-driven and ecologically relevant assessment tool." Vet J. **188**:331-336

Scheff, S. W. and D. A. Price (2003). "Synaptic pathology in Alzheimer's disease: a review of ultrastructural studies." Neurobiol Aging **24**(8): 1029-1046.

Schutt T, Toft N, Berendt M. (2015) "A comparison of 2 screening questionnaires for clinical assessment of canine cognitive dysfunction". Journal of Veterinary Behaviour: Clinical Applications and Research 10(6) p452-458

Siette J, Westbrook RF, Cotman C, Sidhuk K, Zhu W, Sachdev P, **Valenzuela MJ** (2013). "Age-specific effects of voluntary exercise on memory and the older brain" Biological Psychiatry 73(5): 435-42

Siette J (2012) Exercise and Cell Therapy: Restorative Mechanisms for Reversing Age-Related Memory Decline. B. Psychology (Hons.) Submitted for the degree of Doctor of Philosophy. School of Psychology Faculty of Science, University of New South Wales.

Studzinski, C. M., J. A. Araujo, et al. (2005). "The canine model of human cognitive aging and dementia: pharmacological validity of the model for assessment of human cognitive-enhancing drugs." Prog Neuropsychopharmacol Biol Psychiatry **29**(3): 489-498.

Tang, J., H. Xu, et al. (2008). "Embryonic stem cell-derived neural precursor cells improve memory dysfunction in Abeta(1-40) injured rats." Neuroscience Research **62**: 86-96.

Valenzuela, M., K. Sidhu, et al. (2007). "Neural stem cell therapy for neuropsychiatric disorders." Acta Neuropsychiatrica **19**: 11-26.

Valenzuela, M. J., S. K. Dean, et al. (2008). "Neural Precursors from Canine Skin: A New Direction for Testing Autologous Cell Replacement in the Brain." Stem Cells and Development **17**(6): 1087-1094.

Yamasaki, T. R., M. Blurton-Jones, et al. (2007). "Neural stem cells improve memory in an inducible mouse model of neuronal loss." Journal of Neuroscience **27**(44): 11925-11933.
